# Systematic review and meta-analysis to identify meropenem exposures and pharmacological targets predictive of clinical outcomes

**DOI:** 10.1093/jac/dkag230

**Published:** 2026-07-14

**Authors:** Tim Luxton, Robert West, Natalie King, Richard Gould, Christoph Wälti, Jonathan A T Sandoe

**Affiliations:** School of Electronics and Electrical Engineering, University of Leeds, Leeds, UK; Leeds Institute of Health Sciences, University of Leeds, Leeds, UK; Leeds Institute of Health Sciences, University of Leeds, Leeds, UK; Anaesthesia and Intensive Care Medicine, Leeds Teaching Hospitals Trust, Leeds, UK; School of Electronics and Electrical Engineering, University of Leeds, Leeds, UK; Leeds Institute of Medical Research, University of Leeds, Leeds, UK

## Abstract

**Background:**

Meropenem is a broad-spectrum carbapenem reserved for severe infections. Pharmacokinetic variability has been observed in meropenem patients, and its optimal pharmacological target remains unclear. This meta-analysis evaluated meropenem exposure, target attainment, and their association with clinical outcomes to clarify effective pharmacological targets.

**Methods:**

Following PRISMA (PROSPERO CRD42024499652), studies were identified that measured meropenem concentrations in patient samples and reported clinical outcomes. Studies were synthesized and predictors for clinical outcomes were identified by meta-analysis. Patient-level data, provided by authors, were analysed by multivariate logistic regression to identify predictors for clinical outcomes.

**Results:**

In total, 42 studies were included, comprising 2264 patients. Most studies had a high risk of bias in this context. Pooled clinical cure was 70.0% and 30-day mortality 22.2%. Meropenem concentration was not associated with clinical cure (OR 1.01 per mg/L, 95% CI 0.97–1.05, *P* = 0.702), whereas attaining 100%ƒT > MIC was marginally associated with cure (OR 1.02 per 1%, 1.00–1.04, *P* = 0.0552). Higher meropenem exposure was associated with increased 30-day mortality (OR 1.064 per mg/L, 1.008–1.124, *P* = 0.025). In patient-level analysis (six studies, 295 patients), meropenem exposure, SOFA score and age were each independently associated with mortality.

**Discussion:**

Higher meropenem concentrations were associated with increased mortality across both cohort and patient-level analyses, challenging the assumption that universally higher dosing benefits all patients. However, residual confounding by illness severity could not be excluded, thus this finding should be considered hypothesis generating. Attaining 100%ƒT > MIC may improve cure while avoiding excessive concentrations may reduce harm, further research into meropenem personalized dosing is required.

## Introduction

Antimicrobial resistance (AMR) is a worsening global threat; in 2019, almost 5 million deaths were associated with AMR, and 1.25 million deaths were directly attributable to AMR.^1^ AMR has emerged for almost all antibiotics shortly after their introduction into clinical practice, and one of the drivers of AMR is inappropriate dosing of antibiotics.^2^ The World Health Organization (WHO) designate some antibiotics as ‘Reserve’, to be used for severe infections as a ‘last-resort’, to slow the emergence of AMR.^3^ Meropenem is a broad-spectrum beta-lactam antibiotic that has been classified as a ‘Reserve’ antibiotic. Despite this classification, AMR to carbapenems, including meropenem, has been observed in all the ‘ESKAPE’ pathogens, which are associated with high mortality.^4–8^ Meropenem and other beta-lactams are typically administered to patients at a ‘one-size-fits-all’ standard dose, however, large variability in the pharmacokinetics of meropenem and other beta-lactams, particularly in critically ill patients, has been observed.^9,10^ This approach to dosing results in some patients being underdosed, which will exacerbate the emergence of resistance, and others being overdosed, increasing the likelihood of meropenem toxicity. Meropenem toxicity can present as neurotoxicity, seizures, kidney failure, hallucinations or confusion,^11^ and has been reported in up to 50% of meropenem-treated patients with trough concentrations >64 mg/L.^11^

Therapeutic drug monitoring (TDM), or personalized dosing, is the practice of measuring drug concentrations and adjusting dosing to optimize concentrations. Personalized dosing of meropenem is a complex intervention, and many factors can affect its success.^12^ The amount of meropenem in a sample needs to be accurately measured; the current gold-standard method to achieve this is HPLC-MS/MS.^12^

A previous systematic review investigating the clinical impact of carbapenem TDM,^12^ identified that numerous target concentrations were being used, ranging from 60% ƒT > MIC to 100% ƒT > 4-10xMIC. With measured MICs ranging from 0.125 to 32 mg/L, the difference in meropenem exposure between reports could therefore be very large. Although the systematic review could not synthesize reports, it demonstrated that an optimal concentration window for meropenem based on clinical data had not yet been established.

### Rationale and objectives

The rationale behind this systematic review and meta-analysis was to identify reports that had measured and reported meropenem concentrations and/or target indices as well as associated patient clinical outcomes. By synthesizing these reports, it was proposed that an optimal concentration range for meropenem could be identified. Thus, the question this systematic review and meta-analysis set out to answer was ‘what meropenem tissue/blood concentrations or pharmacokinetic/pharmacodynamic indices result in improved clinical outcomes for patients?’.

## Methods

This systematic review was reported according to the Preferred Reporting Items for Systematic Reviews and Meta-Analyses (PRISMA) guidelines.^13^ PROSPERO registration: CRD42024499652.

### Definitions

#### Meropenem administration definitions

Intermittent bolus dosing: short (30-minute) infusions of meropenem given at defined intervals. Extended infusions: 2–3-hour long infusions of meropenem given at defined intervals. Continuous infusions: infusions that run all the time e.g. an 8-hour long infusion given every 8 hours.

#### Meropenem measures

ƒT > MIC: the amount of time unbound meropenem concentrations are above the MIC. *C*_min_: trough concentrations. *C*_ss_: steady-state concentrations. Meropenem exposure was defined as either the *C*_min_ or *C*_ss_, to standardize a measure of the amount of meropenem a patient received across all administration routes. C/MIC was defined as either *C*_min_/MIC or *C*_ss_/MIC ratio, depending on meropenem administration method, and was calculated using the median MIC (2 mg/L) used across all cohorts.

#### Outcome definitions

All-cause mortality: death from any cause during the study follow-up period. 30-day mortality: death from any cause from treatment initiation. In-hospital/ICU mortality, death from any cause while in hospital/ICU for the index admission. Length of stay on ICU/in hospital, total number of days spent on ICU/in hospital during the index admission. Clinical cure: the resolution of signs and symptoms of infection. Acute kidney injury: a rapid decline in renal function, defined using KDIGO or RIFLE criteria (rise in serum creatinine ≥1.5× baseline or ≥26.5 µmol/L within 48 hours, or urine output <0.5 mL/kg/hour for ≥6 hours). Toxicity/adverse events: any harmful or unintended effect attributed to meropenem, including neurotoxicity, hepatotoxicity or gastrointestinal effects, as defined by individual studies. Duration of treatment: total number of days a patient receives meropenem. Readmission: unplanned return to hospital within study defined period. Emergence of AMR: development of resistance to meropenem or other antimicrobials during or following treatment, detected microbiologically.

#### Eligibility criteria

Population: all patients being treated with meropenem for a suspected or confirmed infection were included. Patients treated with meropenem and co-administered with another antibiotic were included. Patients treated with antibiotics other than meropenem were excluded. Intervention/exposure: meropenem treatment where the amount of meropenem in a patient sample had been measured at any time during the course of the treatment. Patients/reports were excluded if meropenem was not measured. Comparators/controls: comparisons of different methods of meropenem administration (i.e. intermittent bolus dosing or continuous infusion); comparisons of different meropenem doses; or comparisons of TDM versus standard care. Outcomes: a number of clinical outcomes were included: mortality (all cause, in-hospital, in-ICU, 30-day), in-hospital stay, length of time on ICU, clinical cure (the resolution of signs and symptoms of infection), acute kidney injury on therapy, toxicity or adverse effects, duration of treatment, readmission, emergence of AMR. Reports that measured meropenem levels but did not report an outcome were excluded. Reports that measured meropenem levels and reported combined outcomes for a number of antibiotics (e.g. beta-lactam antibiotics) were excluded. If this was the case, authors were contacted to provide data of only meropenem patients, if data were not received the report was excluded. Report characteristics: randomized controlled trials, non-randomized trials (cohort studies, quasi-experimental studies) and observational (prospective and retrospective) reports were included. Case reports, reviews, conference abstracts, reports not written in English, animal reports, *in vitro* reports and simulation studies were excluded.

### Information sources and search strategy

Studies were identified from several databases: Cochrane, Dimensions, EMBASE, International Pharmaceutical Abstracts, MEDLINE, Scopus and Web of Science. Searches of databases were carried out from the database inception to 25 March 2025. See Supplementary Material 3 (available as Supplementary data at *JAC* Online) for search terms and search strategy.

### Selection process

The removal of duplicate reports was carried out on EndNote 21 (Clarivate Analytics). The title and abstract screen and the full-text screen were carried out using Covidence systematic review software (Veritas Health Innovation). Title and abstracts followed by full texts of identified reports were screened independently by four researchers (T.L., R.G., C.W. and J.S.). In the title and abstract screen and full-text screen, each report was screened by at least two researchers; a third researcher would independently screen reports in cases in which there was a disagreement.

### Data collection process and data items

Data collection was carried out using Covidence systematic review software (Veritas Health Innovation). A data extraction form was used to collect data from each study. Studies that used analysed data from the same trial were identified and linked. Data were collected by one researcher (T.L.). Data collected were as follows: report data: title, aim of report, report IDs of other reports of this study, lead author contact details, country of study, study funding, potential conflicts of interest, study start date and end date, whether the study was prospective or retrospective; population and setting data: population description, setting, inclusion and exclusion criteria, methods of recruiting patients, total number of patients, groups and numbers per group, baseline imbalances, withdrawals and exclusions, baseline population characteristics (sex, age, race/ethnicity, weight, BMI, comorbidities, APACHE II score, SOFA score, creatinine clearance, creatinine level, pathogens, MIC and whether the MIC used was microbiologically determined or a surrogate MIC, target index, target meropenem concentration, concomitant antibiotics, co-therapies). If a potentially eligible study included aggregated results for other antibiotics as well as meropenem, the authors were contacted to request data specifically for patients treated with meropenem. If data were missing it was assumed that data had not been collected. Intervention data: meropenem dosing and concentration data (meropenem dose, delivery method, dose duration and frequency, whether TDM was used, whether dose adjustments were carried out, meropenem trough level, peak level, Cmin/MIC, ƒT > MIC, other indices reported). If any data surrounding the intervention were missing, it was assumed it had not been collected. Outcome data: all-cause mortality, 30-day mortality, in-hospital mortality, in-hospital stay, length of stay on ICU, clinical cure (that is resolution of signs and symptoms of infection), acute kidney injury, toxicity/adverse events, duration of treatment, readmission, emergence of AMR, other outcomes reported by a report. It was assumed that a particular outcome was not recorded if outcome data were missing.

### Risk of bias assessment

Risk of bias assessment of reports was carried out using Covidence systematic review software (Veritas Health Innovation) and assessed by one researcher (T.L.). The risk of bias assessment tool used depended on the study type. For randomized controlled trials, the Revised Cochrane Risk of Bias Tool for Randomised Trials (RoB2) assessment was used.^14^ For non-randomized intervention trials, the Risk of Bias in Non-Randomised Studies (ROBINS-I) assessment tool was used.^15^ For observational studies, the Risk of Bias in Non-Randomised—of Exposure (ROBINS-E) assessment tool was used.^16^

### Statistical analysis

All analyses were carried out using R and R Markdown, using the metafor (v.4.8-0), meta (v.8.2-1) and mice (v.3.19.0) packages used for meta-analytic models.^17,18^

### Clinical outcome pooled proportions—study-level analysis

For each clinical outcome, descriptive pooled proportions were calculated at the study level, combining cohorts within the same study. Event counts and sample sizes were extracted per study, logit-transformed (escalc, measure = ‘PLO’; metafor), pooled using a random-effects model and back-transformed to proportions for reporting.

### Data preparation and effect size calculation

Study-level data were extracted by cohort. To assess the impact of meropenem exposure, trough (*C*_min_) and steady-state (*C*_ss_) concentrations were used as meropenem concentration. Effect sizes were calculated as logit-transformed proportions using escalc() (measure = ‘PLO’) from the metafor package, with all estimates back-transformed to proportions for reporting.

### Pooled meta-analysis

Pooled estimates for clinical cure (*k* = 34) and 30-day mortality (*k* = 16) were obtained using multivariate random-effects models [rma.mv()] with a two-level nested structure (random = ∼ 1 | Study/Cohort) to adjust for clustering of cohorts within studies, estimated by restricted maximum likelihood, which provides less-biased estimates of the between-study variance compared with full maximum likelihood.^19^

### Heterogeneity

Heterogeneity was quantified using Cochran's *Q* and an *I*^2^ analogue calculated as Σσ^2^/(Σσ^2^ + mean sampling variance).^20^

### Publication bias and sensitivity analysis

Small-study effects were assessed using an adapted Egger’s test, adding the standard error of each effect size as a moderator to the rma.mv() model.^21^ Funnel plots were generated using funnel(). Leave-one-out sensitivity analysis was performed for both meta-analyses, and for each analysis the impact on outcome was recorded. Further leave-one-out sensitivity analyses were performed on the 100% ƒT > MIC–clinical cure and concentration–mortality meta-regressions. For each iteration the 100% ƒT > MIC or concentration coefficient (i.e. OR per 1 mg/L) and its *P* value were recorded to confirm the association's stability. The metafor package in R was used for analyses.

### Meta-regression

Meropenem concentration was entered as a continuous moderator. For mortality outcome, meropenem concentration was mean-centred and both linear and quadratic terms were fitted to test for a nonlinear exposure–mortality relationship, compared using the Akaike information criterion and Bayesian information criterion. Stratified meta-regression analyses were carried out to identify whether there were differences in mortality in patients given meropenem by different routes of administration, and differences in mortality in patients who had sub-therapeutic, therapeutic or supra-therapeutic meropenem exposures. A separate exploratory meta-regression for clinical cure used 100% ƒT > MIC as a continuous moderator (*k* = 8). Cohorts with ƒT > MIC data were also stratified into tertiles, with stratum-specific pooled estimates obtained using a no-intercept categorical moderator model.

### Descriptive analyses

Differences in SOFA score between administration groups of studies that reported a 30-day mortality outcome were analysed with an ANOVA test, after confirming normality with a Shapiro–Wilk test and equality of variance with Bartlett’s test.

### Patient-level analysis

Patient-level data were analysed to investigate the relationship between meropenem exposure and mortality. Continuous variables are presented as median (interquartile range, IQR) and categorical variables as frequency (percentage). For all analyses, paediatric patients were excluded due to differences in pharmacology, particularly in very young patients (median age 1-year). Mortality data were synthesized from studies reporting 28-day mortality,^22,23^ 30-day mortality,^24,25^ ICU or hospital mortality^26^ or mortality with no other descriptors.^27,28^

The association between meropenem and mortality was assessed using multivariable logistic regression. Variables were selected *a priori* on the basis of clinical relevance and existing literature, including meropenem exposure (*C*_min_ or *C*_ss_), route of administration, sex, age, weight, serum creatinine level as an indicator for renal function, SOFA score and whether a microbiologically determined MIC or a surrogate MIC was used in treatment. It was assumed that serum creatinine levels were measured at baseline, eGFR rates were calculated using the Cockcroft–Gault formula,^29^ but were not included in the multivariate analysis as they were derived from the serum creatinine concentration and inaccuracies of eGFR values in critically ill patients.^30^

Missing data were handled using multiple imputation by chained equations, implemented using the mice package in R. Five imputed datasets were generated using predictive mean matching, and results were pooled across imputed datasets using Rubin’s Rules.^31^ The extent and pattern of missing data were assessed before imputation using missing data pattern analysis.

Results of the multivariable logistic regression are presented as OR with 95% CI. Statistical significance was defined as *P* < 0.05.

## Results

### Study selection

**Figure 1.**
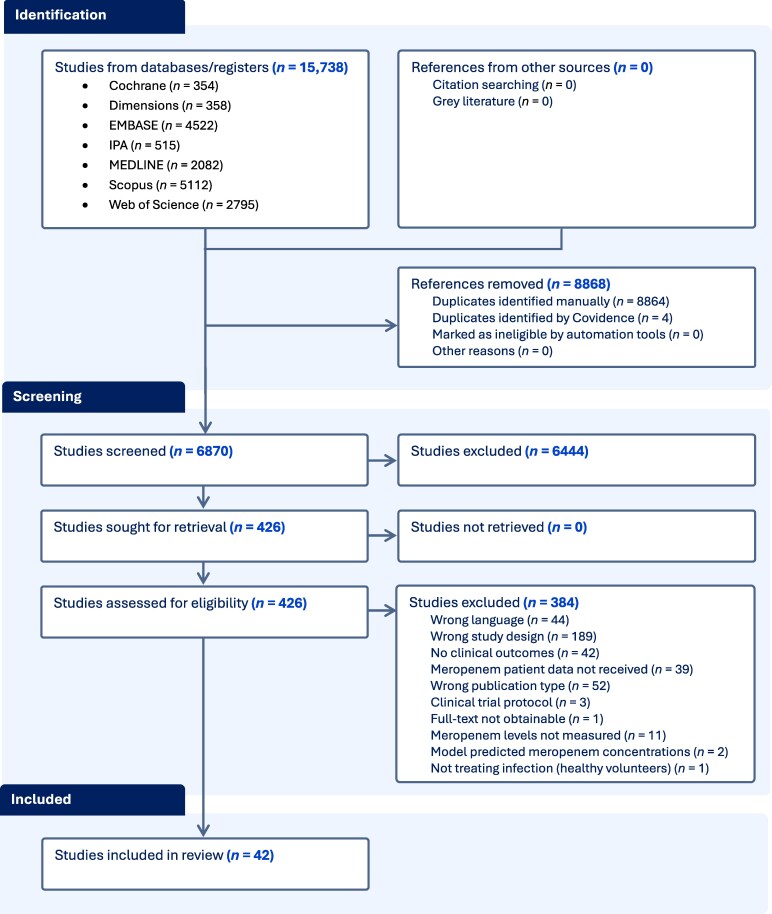
Flow diagram of database search and screening process in accordance to the PRISMA guidelines. Screening carried out in Covidence, figure generated by Covidence.^13.^

Figure 1 displays the search and screening process, as per PRISMA guidelines. After de-duplication, the search returned 6870 studies, of which 6444 were excluded during the title and abstract screen. A further 384 reports were excluded after screening full texts. A number of reports at first appeared to fulfil the inclusion criteria however were excluded as these texts included patients treated with other antibiotics, including meropenem, and reported combined patient demographic data, combined patient outcomes or both. Authors were contacted requesting anonymized patient data for just patients treated with meropenem.^32–70^

### Study characteristics

Descriptive data of the included studies are presented in Table 1. The characteristics of individual studies are presented in Supplementary Material 1^22–28,71–105^: 42 studies were included with 59 different patient cohorts, including 2264 patients. Nineteen studies were retrospective data analyses,^22,25–28,71,72,76,77,80,82,83,86,88,91,92,95,96,98^ and 23 were prospective studies.^23,24,73–75,78,79,81,84,85,87,89,90,93,94,97,99–105^ Meropenem was administered by intermittent bolus dosing in 27 cohorts, by extended infusion in 14 cohorts, and continuous infusion in 18 cohorts. Meropenem concentrations differed according to the route of administration: Where intermittent bolus dosing was used, the median meropenem trough concentration was 2.35 mg/L (IQR: 0.70–4.90 mg/L); for extended infusions, the median meropenem trough concentration was 3.31 mg/L (IQR: 1.75–6.39 mg/L); for continuous infusions, the median meropenem steady-state concentration was 18.85 mg/L (IQR: 14.9–22.05 mg/L) [Kruskal–Wallis, *χ*^2^: 30.5, degrees of freedom (df): 2, *P* < 0.001]. A microbiologically determined MIC was used in 21 cohorts, a surrogate MIC was used in 23 cohorts with 17 of those using a 2 mg/L MIC value and a combination of microbiologically determined or a surrogate MIC in nine cohorts (i.e. a surrogate MIC was used unless a microbiologically determined MIC was available); MICs were not reported in six cohorts. Several different outcomes were reported across studies and scoping study-level pooled proportions were calculated (Table 2), no study specifically investigated acute kidney injury as an outcome. Per-study effect estimates are reported for each outcome in Supplementary Material 2.

**Table 1.** Synthesized characteristics of studies included in the systematic review and meta-analysis

Variable	Synthesized summary
Population category	Critical care patients (*n* = 1361, 60.1%); Critical care paediatric patients (*n* = 328, 14.5%); Patients with febrile neutropenia (*n* = 141, 6.2%); General hospital patients (*n* = 117, 5.2%); Post-neurosurgical meningitis patients (*n* = 82, 3.6%); Elderly patients (*n* = 71, 3.1%); Cystic fibrosis children with acute pulmonary exacerbations (*n* = 40, 1.8%); Critical care patients with BSIs or VAP (*n* = 32, 1.4%); Transplant patients (*n* = 29, 1.3%); Paediatric patients who underwent haematopoietic stem cell transplantation (*n* = 21, 0.9%); Patients with low body weight (*n* = 20, 0.9%); Burns patients (*n* = 16, 0.7%); Patients with G-BSIs (*n* = 6, 0.3%).
Total patients (*n*)	2264
Male sex, *n* (%)	1362 (60.2)
Age (years)	57.8 (IQR: 44.0–64.7)
Weight (kg)	64.0 (IQR: 52.6–72.0)
BMI	25.5 (IQR: 23.2–27.5)
Meropenem dose (g/day)	3 (IQR: 3–3.2)
Administration method	Extended infusion, Continuous infusion, Intermittent bolus
MIC method	Combination, Microbiological, Surrogate
Surrogate MIC value (mg/L)	2 (IQR: 2–2)
Microbiological MIC value (mg/L)	0.45 (IQR: 0.13–4.56)
*C*_min_ (mg/L)	3.30 (IQR: 0.97–6.85)
*C*_ss_ (mg/L)	18.85 (IQR: 14.9–22.05)
100% ƒT > MIC target achievement (% of patients)	55 (IQR: 47.75–83.75)
C/MIC	3.35 (IQR: 1.12–7.05)
Study outcome measures	30-day mortality (*n* = 14), All-cause mortality (*n* = 7), Duration of treatment (*n* = 18), Clinical cure (*n* = 24), Emergence of AMR (*n* = 2), ICU mortality (*n* = 7), In-hospital mortality (*n* = 6), In-hospital stay (*n* = 6), Length of stay on ICU (*n* = 9), Readmission (*n* = 2), Toxicity/adverse events (*n* = 7)

BSI = bloodstream infection; VAP = ventilator-associated pneumonia.

**Table 2.** Clinical outcomes reported alongside number of studies reporting outcome, number of patients, number of events and study-level pooled proportions or pooled estimates of each outcome

Outcome	Studies (*n*)	Patients (*n*)	Events (*n*)	Pooled proportion (95% CI)	Pooled estimate (95% CI)
30-day mortality	14	812	221	23% (16%–32%)	—
All-cause mortality	7	224	46	19% (10%–34%)	—
Duration of treatment (days)^a^	18	1147	—	—	10 (9–12)
Clinical cure	24	1186	790	69% (63%–75%)	—
Emergence of AMR	2	56	2	5% (1%–16%)	—
In-hospital mortality	6	462	142	29% (18%–43%)	—
In-ICU mortality	7	428	105	27% (18%–37%)	—
Length of stay in hospital (days)^a^	6	358	—	—	29 (15–54)
Length of stay on ICU (days)^a^	9	786	—	—	18 (13–24)
Readmission	2	166	14	9% (5%–15%)	—
Toxicity/adverse events	7	439	47	8% (1%–43%)	—

^a^Continuous outcome reported as pooled mean (95% CI).

### Risk of bias in studies

Risk of bias was assessed by different tools according to their study type, there were four RCTs^81,94,99,103^ assessed using the ROB2 tool (Figure S1), one non-randomized intervention study^71^ assessed using the ROBINS-I tool (Figure S2) and 37 observational exposure studies^22–28,72–80,82–93,95–98,100–102,104,105^ that were assessed using the ROBINS-E tool (Figure S3). In general, studies had a high risk of bias, typically because most studies did not have a clinical outcome as their primary outcome, which resulted in a study design that had a high risk of bias when assessing the study’s clinical outcome findings. For example, studies examining meropenem exposure focused on identifying factors associated with target attainment rather than clinical outcomes and did not report analyses examining predictors of mortality or other clinical endpoints. Therefore, these studies were recorded as having a very high risk of bias as the effects of confounding factors on target attainment were assessed rather than those on clinical outcomes.

### Results of syntheses

Cohort-level syntheses of clinical cure (*n* = 1186) and 30-day mortality (*n* = 812) were carried out.

### Clinical cure of infection

**Figure 2.**
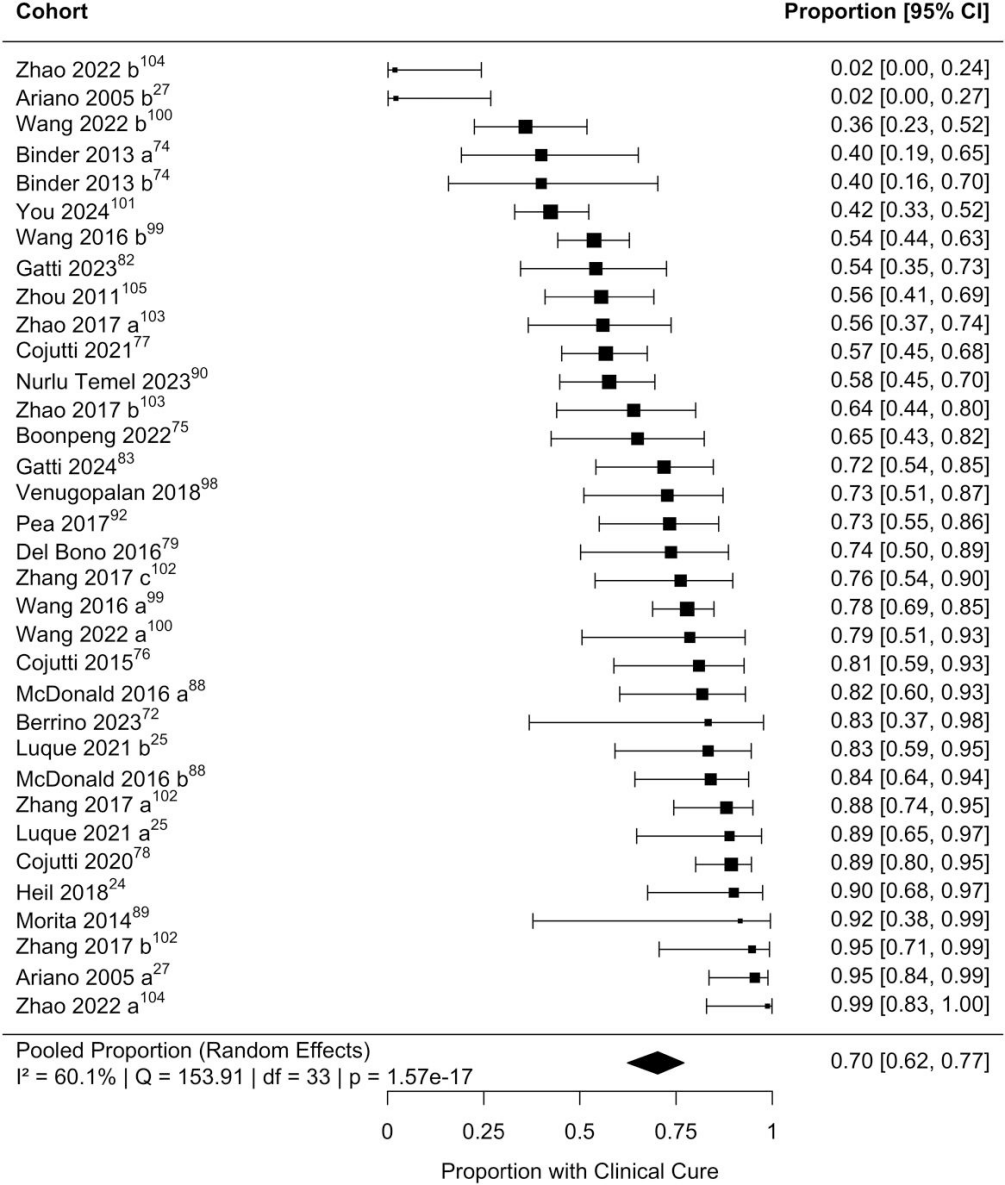
Forest plot of meta-analysis of studies reporting clinical cure, that is the resolution of signs and symptoms of infection. Point estimates and 95% CI for each cohort are displayed.

Clinical cure was reported as an outcome in 24 studies across 34 different patient cohorts and included 1186 patients (Table S1). Summary estimates and CIs for each cohort are presented in Figure 2. The pooled proportion of patients who were clinically cured was 70.0% (95% CI: 0.62–0.77). There was substantial heterogeneity across cohorts (*I*^2^: 60.1%, *Q*: 153.91, df: 33, *P* < 0.001).

The pooled proportion estimate was robust to the removal of any individual cohort, demonstrated by a ‘leave-one-out’ analysis (Figure S4a). The funnel plot for this analysis shows rightward asymmetry (Figure S4b); however, evidence of publication bias or small-study effects was not statistically significant. Estimates ranged from 68% to 71% across iterations. This showed that no one cohort influenced the overall result to a large extent and indicates the stability of the synthesized estimate.

Meropenem concentration, either trough concentration (*C*_min_) or steady-state concentration (*C*_ss_), depending on administration method (intermittent versus continuous infusion), was used as a continuous moderator in a meta-regression analysis that showed there was no relationship between meropenem concentration and clinical cure (OR: 1.01, 95% CI: 0.97–1.05, *P* = 0.702) (Figure S5).

**Figure 3.**
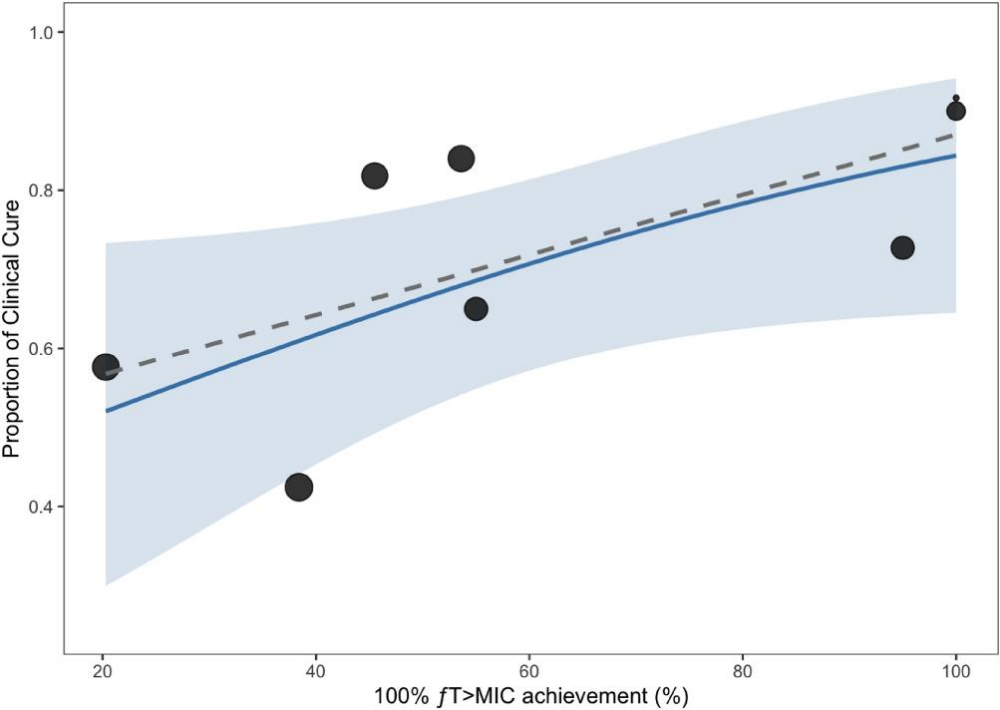
Meta-regression of clinical cure meta-analysis moderated by the percentage of patients achieving 100% ƒT > MIC. Bubbles represent individual cohorts. Size of bubbles indicates weighting of each cohort, adjusted for clustering within studies. Blue solid line and light blue ribbon: Linear meta-regression curve and 95% CI (OR 1.02, 95% CI: 1.00–1.04, *P* = 0.0552). Grey dotted line shows the unweighted ordinary least squares fit, not accounting for study weights or random effects, for reference only.

The percentage of patients achieving the pharmacological target of 100% ƒT > MIC was also used as a continuous moderator and showed a trend between achieving 100% ƒT > MIC and clinical cure (OR 1.02 per 1% increase, 95% CI: 1.00–1.04, *P* = 0.0552) (Figure 3). A leave-one-out sensitivity analysis, in which the meta-regression was refitted with each cohort removed in turn, showed that the positive association between 100% ƒT > MIC achievement and clinical cure was consistent in direction across all iterations (odds ratio 1.02–1.03 per 1%). However, as only eight cohorts reported both 100% ƒT > MIC and clinical cure, this analysis is underpowered to reliably assess the stability of statistical significance, which varied from *P* = 0.032 and *P* = 0.195, and should be interpreted as supporting the consistency of the direction of the association rather than its robustness. This trend was also seen in the stratified analysis (Figure S6) where cohorts were divided into three strata with an equal number of cohorts in each stratum, low 100% ƒT > MIC achievement (<48.2%), moderate achievement (48.2%–81.7%) and high achievement (>81.7%). The clinical cure meta-analysis was also stratified by route of administration, intermittent bolus dosing, extended infusion and continuous infusion. The stratification showed that administration route had no impact on the proportion of patients with clinical cure (Figure S7).

### 30-day mortality

**Figure 4.**
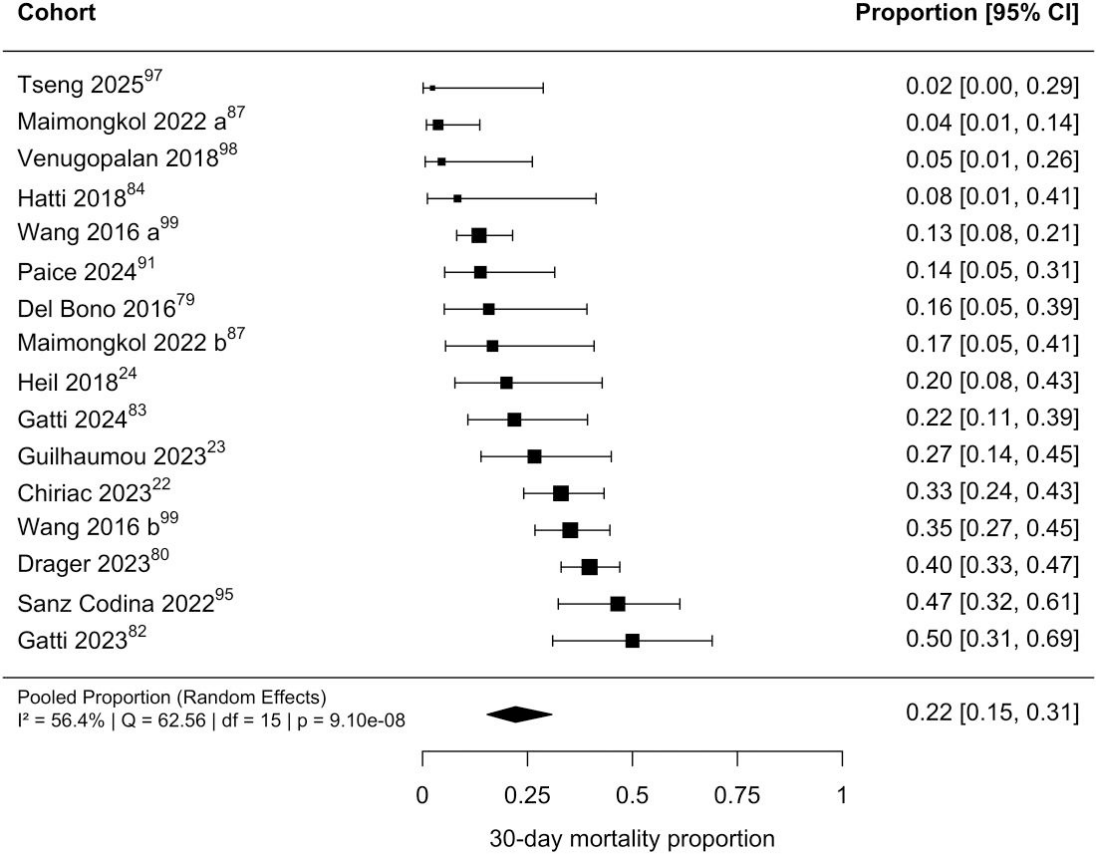
Forest plot of meta-analysis of studies reporting 30-day mortality. Point estimates and 95% CI for each cohort are displayed.

There were 14 studies with 16 cohorts that reported 30-day mortality, including 812 patients. Summary estimates and CIs for each cohort are presented in Figure 4; baseline demographics are presented in Table S2. The pooled proportion of 30-day mortality was 22.2% (95% CI: 15.4%–30.8%). There was substantial heterogeneity across cohorts (*I*^2^: 56.4%, Q: 62.56, df: 15, *P* < 0.001).

The pooled 30-day mortality estimate was robust to the leave-one-out sensitivity analysis and gave an estimate range of 21%–24% (Figure S8a). The extended infusion arm of Maimongkol *et al*.^87^ had the most influence on the pooled estimate, as seen with the largest shift in the leave-one-out analysis. A statistically significant funnel plot asymmetry (*b*: −2.77, 95% CI: −4.11−1.43, *P* < 0.0001) was observed from Egger’s test (Figure S8b), with 30-day mortality in smaller cohorts reported systematically lower than in larger cohorts. This may indicate publication bias, or this may be explained by the fact that larger studies had higher rates of mortality. The three studies with the lowest number of patients that died after 30-days, had patient populations treated in the general hospital (*n* = 54), while for the rest of the studies patients were treated in the ICU (*n* = 758). These findings should be considered when interpreting the pooled estimate of 30-day mortality.

**Figure 5.**
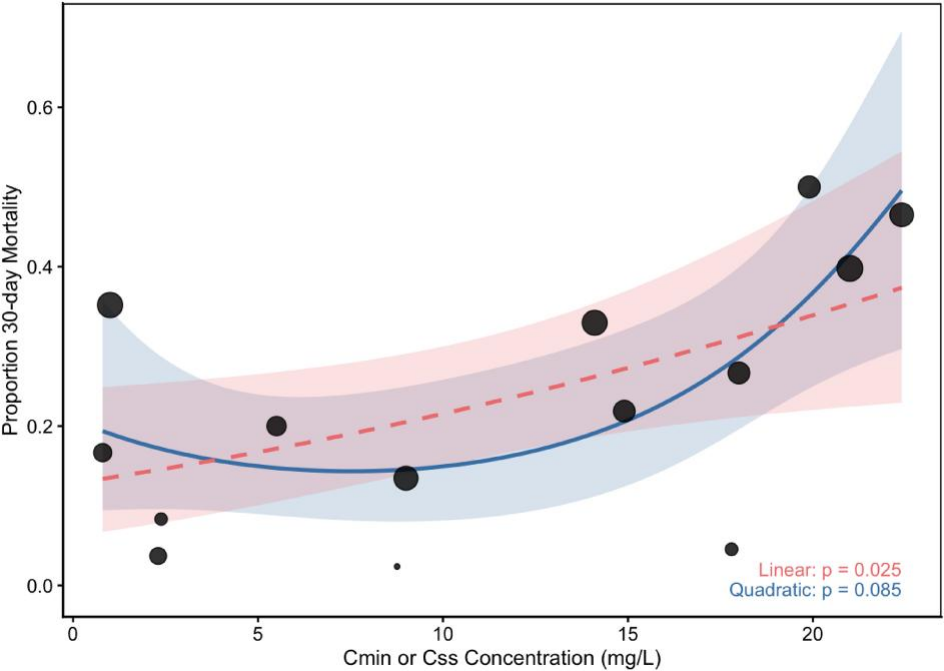
Bubble plot of 30-day mortality meta-regression moderated by meropenem exposure (*C*_min_ or *C*_ss_). Bubbles represent individual cohorts. Size of bubbles indicates weighting of each cohort, adjusted for clustering within studies. Red dotted line and pink ribbon denotes a linear meta-regression curve fit. Blue solid line and light blue ribbon denotes a quadratic meta-regression curve fit.

Figure 5 displays exploratory analyses of the effect of meropenem exposure on 30-day mortality, with a linear and quadratic meta-regression of meropenem exposure plotted against 30-day mortality. The linear relationship gave an OR of 1.064 per mg/L of meropenem increased (OR: 1.064, 95% CI: 1.008–1.124, *P* = 0.025), suggesting the more meropenem the higher the probability of 30-day mortality. A quadratic curve was also fitted to the data as there is a visual ‘U-shape’ to the data (OR: 1.008, 95% CI: 0.999–1.017, *P* = 0.085). Further leave-one-out sensitivity analyses were carried out exploring the impact that cohorts had on the slopes of the linear and quadratic relationships. For the linear association (Figure S9), the direction of the slope was entirely unchanged, and the strength of the association also changed very little with ORs ranging between 1.05 and 1.11 per mg/L of meropenem. The statistical significance remained in 10/14 cases, when Drager^80^, Gatti 2023^82^, Maimongkol^87^ or Sanz Codina^95^ was removed the *P* value rose just above the 0.05 threshold (to between 0.07 and 0.09). The quadratic slope was not robust and was not significant in the majority of cases (Figure S10). These sensitivity analyses show that the association between meropenem concentration and 30-day mortality is consistent and not an artefact from one study. However, it does not demonstrate causation.

Figure S11 shows a 30-day mortality meta-regression using meropenem route of administration as a categorical moderator. Pooled 30-day mortality estimates differed between groups, continuous infusion gave a 19% mortality (95% CI: 0.09–0.36), extended infusion gave an 11% mortality (95% CI: 0.05–0.24), and intermittent bolus dosing gave a 29% mortality (95% CI: 0.20–0.41). Relative to continuous infusion, extended infusion resulted in a significantly lower mortality (*P* = 0.029); there was no difference in mortality when continuous infusion was compared to intermittent bolus dosing (*P* = 0.292). Of studies that had 30-day mortality data and SOFA score data, there was no difference in condition severity between administration groups (*P* = 0.32).

Some studies aimed to meet a concentration ‘window’, higher than sub-therapeutic concentrations and lower than supra-therapeutic concentrations, using a surrogate 2 mg/L MIC value.^22,23,25,76–78,84^ The target of 100% ƒT > 4-10x MIC was tested to see whether there were differences in mortality; meropenem exposure was stratified into sub-therapeutic (<8 mg/L), therapeutic (8–20 mg/L) and supra-therapeutic (>20 mg/L). Pooled 30-day mortality estimates identified 30-day mortality was significantly higher (*P* < 0.001) in supra-therapeutic group (43%, 95% CI 0.21–0.69), compared with the therapeutic group (22%, 95% CI 0.13–0.36) and sub-therapeutic group (16%, 95% CI 0.08–0.31).

Owing to limitations in the dataset, it was not possible to include other moderators to the model, such as severity of illness, source of infection, susceptibility or renal function. Any further moderation of the meta-regression would result in saturation of the model and be underpowered.

### Patient-level analysis

A number of studies included data for meropenem-treated patients aggregated with other antibiotics. All authors of included papers and papers that could be included with meropenem patient data were contacted requesting patient-level data. Data were kindly provided by several authors for seven studies, involving a total of 312 patients.^22–28^

Table 3 summarizes the patient-level data received from authors. Most patients were adult critically ill patients, febrile neutropenic patients and transplant patients; one study included 17 paediatric patients: these patients were excluded from the analyses, leaving 295 patients in the analysis. The extent and pattern of missing data was plotted (Figure S12). The only clinical outcome available for all studies was mortality. In a complete case analysis, 165 observations would be excluded, therefore data were imputed by multiple imputation, imputed values were checked visually by a strip plot (Figure S13).

**Table 3.** Patient-level analysis: Summary statistics of studies which provided patient-level data for analysis

Study	*n*	Patient Type	Administration	Male Sex—*n* (%)	Age—Median (IQR)	Weight (kg)—Median (IQR)	Serum Creatinine (mg/dL)—Median (IQR)	eGFR (mL/min)—Median (IQR)	SOFA—Median (IQR)	APACHE—Median (IQR)	Susceptibility	MIC (mg/L)—Median (IQR)	Concentration (mg/L)—Median (IQR)	C/MIC—Median (IQR)	Cure—*n* (%)	Death—*n* (%)
Ariano 2005^27^	66	Febrile Neutropenia	Intermittent Bolus	34 (51.5%)	38.5 (23.8)	62.5 (13.6)	0.7 (0.2)	105.9 (50.1)	NA (NA)	NA (NA)	Measured	0.5 (0)	0.02 (0.04)	0 (0.1)	44 (66.7%)	6 (9.1%)
Chiriac 2023^22^	91	Critical care	Continuous Infusion	60 (65.9%)	73 (18)	80 (20)	1.3 (1.7)	45.3 (53.1)	8 (7)	22 (10)	Surrogate	2 (0)	13.8 (5.3)	6.9 (2.7)	0 (NaN%)	30 (33%)
Guilhaumou 2023^23^	29	Critical care	Continuous Infusion	17 (58.6%)	64 (22)	73.5 (18.5)	1.1 (0.8)	51.2 (85.7)	NA (NA)	NA (NA)	Surrogate	2 (0)	18.7 (12.8)	9.3 (6.4)	0 (NaN%)	7 (24.1%)
Heil 2018^24^	20	Critical care	Extended Infusion	10 (50%)	55.5 (24.8)	89.7 (43)	0.7 (0.9)	78.9 (90.9)	4 (2.5)	15 (8)	Measured	0.25 (0.62)	5.5 (13.2)	13 (24.1)	18 (90%)	4 (20%)
Luque 2021^25^	18	Critical care	Continuous Infusion, Extended Infusion	10 (55.6%)	61.5 (19.5)	45 (8.5)	0.7 (1)	58.4 (54.9)	4 (4.8)	15.5 (8.2)	Measured and Surrogate	2 (1.38)	11.6 (20.1)	7.5 (18.2)	16 (88.9%)	3 (16.7%)
Ragonnet 2024^28^	17	Paediatrics	Continuous Infusion, Intermittent Bolus	11 (64.7%)	1 (4.8)	6.3 (8)	4 (3.4)	3.2 (12)	NA (NA)	NA (NA)	Surrogate	2 (0)	15.2 (19.1)	7.6 (9.6)	0 (NaN%)	6 (35.3%)
Taccone 2021^26^	71	Transplant	Intermittent Bolus	38 (53.5%)	55 (19.5)	60 (19)	1.1 (0.9)	55.6 (48.7)	9 (3)	14 (7)	Surrogate	2 (0)	11.5 (22.5)	5.8 (11.3)	0 (NaN%)	18 (25.4%)

Measured = microbiological MIC; Surrogate = surrogate MIC; CNA = not available; NaN = not a number (no patients with outcome recorded).

Exploratory unadjusted logistic regression analyses were carried out on potential predictors of mortality on the imputed data, meropenem exposure, C/MIC, administration route, whether a microbiologically determined MIC or a surrogate MIC was used, baseline serum creatinine, SOFA score and age (Table S3).

Figure S14 shows a statistically significant association between mortality and meropenem concentration with an OR of 1.04 per mg/L increase of meropenem (OR: 1.04, 95% CI: 1.02–1.06, *P* < 0.001), associating higher meropenem concentrations with a higher probability of mortality. This association was less pronounced when taking into account susceptibility using a 2 mg/L surrogate MIC value (OR: 1.02, 95% CI: 1.00–1.03, *P* = 0.051). Continuously infused patients had a higher mortality proportion when compared with intermittent bolus dosing and extended infusion patients (Figure S15a), this may be due to the fact sicker patients will tend to receive continuous infusions rather than bolus dosing or extended infusions.

A univariate regression looking at the impact of a measured versus surrogate MIC on mortality (Figure S15b) showed that when a patient has an infection where the susceptibility has been confirmed microbiologically, rather than using a surrogate MIC value (i.e. a worst-case scenario clinical breakpoint, 2 mg/L), mortality was significantly less.

Table 4 shows the results of the multivariate logistic regression for mortality. As a large number of MIC values were surrogate clinical breakpoints, MIC was not included in the model to avoid introducing bias. Variables derived from others, such as BMI, eGFR or C/MIC, were not included to avoid multicollinearity. After adjustment, three variables were statistically significantly associated with mortality: SOFA score (OR: 1.24 per point, 95% CI: 1.05–1.46, *P* = 0.017), meropenem *C*_min_ or *C*_ss_ (OR: 1.02 per mg/L, 95% CI: 1–1.04, *P* = 0.033) and age (OR: 1.03 per year, 95% CI: 1–1.05, *P* = 0.034). The receiver operating characteristic curve (Figure S16a, AUC = 0.819, 95% CI: 0.766–0.872) demonstrates that the model distinguished survivors from non-survivors well. Scattering in the predicted probability scatter plot (Figure S16b) is very wide at all concentration ranges indicating that while meropenem concentration may be associated with mortality, there are other, unknown, interacting variables.

**Table 4.** Patient-level analysis: Multivariate logistic regression for mortality (multiply imputed data, *m* = 5)

Meropenem exposure				
Variable	OR	95% CI	*P* value	df
*C*_min_ or *C*_ss_ (mg/L)	**1.02** (per mg/L)	**1–1.04**	0.033 *	**281**.**9**
Extended infusion	4.38	0.54–35.37	0.157	23.5
Continuous infusion	1.38	0.44–4.3	0.561	23.1
Sex	1.17	0.6–2.27	0.65	219.5
Age (years)	**1.03** (per year)	**1–1.05**	0.034 *	**230**.**9**
Weight (kg)	0.99 (per kg)	0.98–1.01	0.578	193.0
Serum creatinine (mg/dL)	0.95 (per mg/dL)	0.73–1.26	0.736	54.3
SOFA score	**1.24** (per point)	**1.05–1.46**	0.017 *	**8**.**9**
Measured versus surrogate MIC	1.89	0.23–15.56	0.517	9.8

df reflect Rubin’s rules pooling across *m* = 5 imputed datasets. Reference categories: Intermittent Bolus (Administration route), Male (Sex), Measured MIC (Susceptibility to Meropenem). *P **<** **0.05**.

## Discussion

This systematic review selected studies in which meropenem concentrations were measured during treatment of infection, and patient clinical outcomes were reported. The meta-analysis showed a marginally significant trend towards higher rates of clinical cure associated with achieving the pharmacological target of 100% ƒT > MIC, but there was no association between meropenem concentrations (either *C*_min_ or *C*_ss_) and clinical cure. Both meta-analysis and multivariate logistic regression of patient-level data identified a statistically significant relationship between meropenem concentrations and mortality.

The lack of a detectible association between clinical cure and meropenem concentration but a marginal association between clinical cure and the achievement of 100% ƒT > MIC suggests that meeting a MIC moderated target index mattered more for treating infection than simply achieving higher meropenem levels. While there are limitations to this analysis, this finding supports the use of a 100% ƒT > MIC target when carrying out meropenem TDM.

The finding of increased mortality with increased meropenem levels was unexpected and prompted scrutiny. The relationship between meropenem exposure and mortality visually appeared to be U-shaped with higher mortality at both lower and higher meropenem levels, a pattern also reported in patients treated with piperacillin.^106^ It is plausible that increased mortality at low concentrations could arise from under-treatment and increased mortality at higher levels could be driven by toxicity. A leave-one-out sensitivity analysis of the U-shaped fit showed that the U-shaped relationship was not robust. However, the leave-one-out analysis of the linear relationship, where higher meropenem levels were associated with higher mortality, was robust in both magnitude and direction, the OR varied very little and reached statistical significance in 10/14 analyses (Figure S9). The analysis of the relationship between mortality and meropenem levels may be confounded by severity of illness, worse renal function in sicker patients (allowing meropenem to accumulate) or another confounding variable. However, in the patient-level analysis, the mortality-meropenem association was also observed. Importantly, in the multivariate analysis, serum creatinine was included as a potential confounding factor and the relationship between higher meropenem levels and higher mortality persisted. The validity of this analysis was supported by the finding of increased mortality in patients with higher SOFA scores and increased age. SOFA score had a greater effect on mortality than meropenem concentration or age, yet there may still be an effect due to meropenem concentration. Meropenem concentrations have previously been associated with neurotoxicity, with trough levels of 64.2 mg/L being associated with neurotoxicity and 44.5 mg/L being associated with nephrotoxicity in 50% of ICU patients.^50^ From available data, it cannot be ruled out that higher mortality is caused by confounding of condition severity, or another confounding factor, nor can it be ruled out that higher meropenem levels are contributing towards higher mortality; therefore, this finding should be considered hypothesis generating.

A recent meta-analysis including 1075 patients found all-cause mortality was not significantly different between patients treated with continuous versus intermittent meropenem infusions (RR = 0.89; 95% CI, 0.75–1.04; *P* = 0.15; *χ*^2^ = 5.75; *I*^2^ = 30%),^107^ while a previous meta-analysis of prolonged infusion (extended infusion or continuous infusion) versus intermittent bolus dosing of meropenem including 951 patients found a significant reduction in mortality associated with prolonged infusion (risk ratio 0.66, 95% CI 0.50–0.88).^108^ In a multicentre, randomized controlled trial of continuous versus intermittent beta-lactam infusions, a subgroup analysis of meropenem-treated patients found no statistically significant impact on 90-day mortality.^109^

The most recent systematic review and the recent large BLING III trial suggest that extended meropenem infusions are not associated with improved mortality compared with bolus dosing.^107,109^ Taken together with the findings of this meta-analysis and patient-level analysis, target attainment of 100% ƒT > MIC may improve clinical cure but excessive meropenem concentrations may also be an independent predictor of mortality: therefore, more precise dosing may be necessary to ensure safety while treating infection. This interpretation needs to be taken with caution given the limitations in the data and more studies are needed to validate the observed relationship between increasing meropenem concentration and higher mortality.

In terms of other outcomes, a previous meta-analysis found continuous infusion was associated with a statistically significant reduction in days on ICU, higher ORs of cure and reduced duration of meropenem treatment compared with intermittent infusion.^107^ It may be that personalized meropenem dosing, rather than increasing meropenem exposure for everyone, might improve the outcome of an infection episode.

### Limitations

There was high heterogeneity between studies, a high risk of bias for the patient centred outcomes and small sample sizes in many studies. For several clinical outcomes of interest, there were insufficient data to enable meaningful meta-analyses. Several trials that included potentially eligible patients could not be included in this analysis because data were presented for patients treated with more than one beta-lactam, without providing data specifically for meropenem-treated patients.

Owing to limitations in the data, it was not possible to carry out meaningful meta-analyses on all outcomes, and inclusion of confounding factors was not possible due to the format of the data; for example, recording of renal function did not allow separation of chronic kidney disease from acute kidney injury at the time of acute illness as only a single baseline creatinine was provided. Cohorts managed in different ways within one study cannot be considered independent datasets, so analysis of the clinical cure outcome included fitting of a random-effects model and were weighted to account for clustering of cohorts within studies. The CIs in each cohort of the leave-one out analysis of pooled estimates for treatment effectiveness were wide, indicating high uncertainty in individual results, which may be a result of the small sample size in each cohort. Both meta-regressions used data averaged across cohorts rather than individual patients (ecological bias), so a pattern seen across cohorts may not hold for individual patients. The link between higher meropenem levels and mortality could therefore be explained by differences between cohorts, such as illness severity, rather than by meropenem levels itself. There were too few cohorts to allow for reliable adjustment of further variables, as doing so would have made the model unreliable; this should be considered when interpreting the findings. A patient-level analysis was carried out to provide individual level evidence, less susceptible to the ecological bias of the meta-analysis.

In the patient-level analysis, different mortality measures were combined, 28-day, 30-day, ICU or hospital, and mortality with no other descriptors. However, a positive association between increasing SOFA score and increasing age and mortality is expected and validates the patient-level model to a certain extent. Data provided on renal function meant it was not usually possible to separate acute kidney injury in the acute setting compared with underlying chronic kidney disease; rather than ignore renal function we included serum creatinine levels as a continuing variable. We assumed that the measured serum creatinine levels from authors’ data tables were measured at baseline. The data for children could not be analysed separately as there were too few patients to enable analysis. Children were excluded from the adult analysis given important differences in pharmacokinetics and dosing.

Missing data, i.e. values not reported and assumed not collected, were handled using multiple imputation by chained equations, which assumes data are missing at random. If data were missing not at random, for example, if sicker patients were less likely to have pharmacokinetic measurements recorded, this assumption may be violated and residual bias cannot be excluded. The extent of missing data was reported in Figure S12. Of the variables included in the multivariate analysis, few had missing data. Of the 295 patients included in the analysis, SOFA score had data for 196 patients (66%), serum creatinine had data for 276 patients (94%) and data for patient weight was available for 294 patients (99.7%); datasets were complete for all other variables included in the multivariate analysis. Given the limited size of the dataset, these analyses are considered exploratory.

In both meta-analyses and the patient-level analysis, ‘meropenem exposure’ included both trough and steady-state concentrations, which needs to be considered when interpreting these results. Being a time-dependent drug, combining meropenem exposure on the minimum maintained concentration gives a lowest drug concentration measure.

### Conclusion

This systematic review and meta-analysis identified a marginally significant association between achievement of the 100% ƒT > MIC target and clinical cure, while higher meropenem exposure was not associated with cure but was associated with increased 30-day mortality. The finding of increased mortality with higher meropenem levels was strengthened by its observation in both the cohort-level meta-regression and our multivariable (adjusted) patient-level analysis. Given the high heterogeneity, high risk of bias and limited data, residual confounding by condition severity or other unmeasured factors cannot be ruled out. Therefore, this finding should be considered hypothesis generating. Adequately powered prospective studies should be considered to investigate further. Taken together, these results challenge the assumption that universally higher meropenem dosing is beneficial for patients and suggest that attaining 100% ƒT > MIC while avoiding excessive concentrations may be beneficial. The role of specific therapeutic targets and TDM in meropenem treatment requires further research.

## Supplementary Material

dkag230_Supplementary_Data

## Supplementary data

Figures S1–S16 and Tables S1 to S3 are available as Supplementary data at *JAC* Online.
