# Supplementary material for: Systematic review and meta-analysis to identify meropenem exposures and pharmacological targets predictive of clinical outcomes
Source: J Antimicrob Chemother. 2026 Jul 14;81(8):dkag230. doi: 10.1093/jac/dkag230 (PMC13364777; doi:10.1093/jac/dkag230)
Supplement: dkag230_Supplementary_Data [file dkag230_supplementary_data.docx]

**Systematic review and meta-analysis to identify meropenem exposures and pharmacological targets predictive of clinical outcomes.**

Supplementary materials contents

- Supplementary material 1 – Study characteristics; **page 1 – 4.**
- Supplementary material 2 – a-k) Forest plots of per study effect estimates for each clinical outcome assessed; **page 5 – 8.**
- Figure S1: Quality assessment of included randomised controlled trials using the Risk of Bias in Randomised Trials (RoB 2) tool; **page 9.**
- Figure S2: Quality assessment of non-randomised trials using the Risk of Bias in Non-randomised Intervention Studies (ROBINS-I) tool; **page 9.**
- Figure S3: Quality assessment of observational or exposure studies using the Risk of Bias in Non-Interventional Studies – Exposure (ROBINS-E) tool; **page 10.**
- Table S1 – Baseline demographics of cohorts reporting a clinical cure outcome; **page 11 – 12.**
- Figure S4 - a) Leave-one-out sensitivity analysis of clinical cure (effective treatment) meta-analysis and impact on pooled proportion. Red dotted line – pooled proportion when all studies included. Blue points and bars - Point estimates and 95% CI. b) Funnel plot with 95% pseudo-confidence intervals. Dots represent individual cohorts; **page 13.**
- Figure S5 - meta-regression of clinical cure meta-analysis moderated by meropenem exposure. Bubbles represent individual cohorts. Size of bubbles indicates weighting of each cohort, adjusted for clustering within studies. Blue solid line and light blue ribbon: Linear meta-regression curve and 95% CI. Grey dotted line: unweighted ordinary least squares (OLS) fit, not accounting for study weights or random effects, shown for reference only; **page 14**.
- Figure S6 – Forest plot of meta-regression of clinical cure meta-analysis moderated by stratified percentage of patients achieving 100% ƒT>MIC. 100% ƒT>MIC stratified into low achievement (< 48.2%), moderate achievement (48.2 – 81.7%), and high achievement (>81.7%); **page 15.**
- Figure S7 - Proportion of patients achieving clinical cure (effective treatment) by meropenem administration route. Points represent individual cohort estimates; large, filled circles indicate pooled estimates with 95% confidence intervals; **page 16.**
- Table S2 – Baseline demographics of cohorts reporting a 30-day mortality outcome; **page 17.**
- Figure S8 - a) Leave-one-out sensitivity analysis of 30-day mortality meta-analysis and impact on pooled proportion. Red dotted line – pooled proportion when all studies included. Blue points and bars - Point estimates and 95% CI. b) Funnel plot with 95% pseudo-confidence intervals. Dots represent individual cohorts; **page 18.**
- Figure S9 - a) Leave-one-out sensitivity analysis of the linear concentration-mortality slope odds ratio. b) Leave-one-out sensitivity analysis of the linear concentration-mortality slope p-value; **page 19.**
- Figure S10 - a) Leave-one-out sensitivity analysis of the quadratic concentration-mortality slope. b) Leave-one-out sensitivity analysis of the quadratic concentration-mortality slope p-value; **page 19.**
- Figure S11 – Forest plot of cohorts and proportion of 30-day mortality in patients, stratified by meropenem administration route; **page 20.**
- Figure S12 - Patient level analysis: Missing data pattern prior to multiple imputation. Blue cells indicate observed data; pink cells indicate missing values. Numbers on the right indicate the count of missing variables per row pattern; numbers at the bottom indicate the total number of missing observations per variable; **Page 21.**
- Figure S13 - Patient level analysis: Stripplot of observed and imputed values across five imputed datasets. Blue points represent observed values; pink points represent imputed values. Imputation number 1–5 reflects each of the five imputed datasets; column 0 represents the original observed data. Distributional similarity between observed and imputed values supports the plausibility of the imputation; **Page 21.**
- Table S3 – Patient level analysis: Unadjusted associations with 30-day mortality (multiply imputed data, m=5); **Page 22.**
- Figure S14 – Patient level analysis: a) plot of unadjusted logistic regression and 95% CI of the association between meropenem exposure (Cmin or Css) and probability of mortality. b) and c) box plot of survivors and non-survivors plotted by meropenem concentration. d) and e) density plot of meropenem exposure by probability of mortality; **Page 22.**
- Figure S15 - Patient level analysis: Unadjusted logistic regression of a) administration route and b) whether a microbiologically determined or a surrogate MIC was used for treatment. **Page 23.**
- Figure S16 a) ROC curve of multivariate logistic regression. b) Predicted Probability plot of mortality. **Page 23.**
- Supplementary material 3 – Search strategy. **Page 24 – 31.**

Supplementary material 1 – Study Characteristics

| Study ID | Population category | n | Group | Male sex, (n) | Age | Weight (kg) | BMI | Daily Dose (g) | Administration | Surrogate / measured MIC | Surrogate MIC (mg/L) | Microbiological MIC (mg/L) | Cmin (mg/L) | Css (mg/L) | 100% ƒT>MIC (%) | Outcome 1 | Outcome 2 | Outcome 3 | Outcome 4 | Outcome 5 | Outcome 6 |
| --- | --- | --- | --- | --- | --- | --- | --- | --- | --- | --- | --- | --- | --- | --- | --- | --- | --- | --- | --- | --- | --- |
| Aldaz 2021 | Critical care patients | 77 | Cohort A (TDM) | 47 | 64.44 | 72.01 | 25.54 | 3 | Extended Infusion | Combination | 1.00 | 0.25 | 6.69 |  |  | In-hospital mortality | In-hospital stay | Length of stay on ICU | Readmission |  |  |
| Aldaz 2021 | Critical care patients | 77 | Cohort B (standard care) | 46 | 68.68 | 68.96 | 24.54 | 3 | Extended Infusion | Microbiological | 1.00 | 0.25 |  |  |  | In-hospital mortality | In-hospital stay | Length of stay on ICU | Readmission |  |  |
| Ariano 2005 | Patients with febrile neutropenia | 44 | Responders | 24 | 32 | 62.5 |  | 3 | Intermittent bolus | Surrogate | 0.13 | 0.50 | 0.02 |  |  | Effective treatment | Duration of treatment |  |  |  |  |
| Ariano 2005 | Patients with febrile neutropenia | 22 | Non-responders | 10 | 46 | 62.5 |  | 3 | Intermittent bolus | Surrogate | 0.50 | 3.20 | 0.02 |  |  | Effective treatment | Duration of treatment |  |  |  |  |
| Berrino 2023 | Patients with G- BSIs | 6 |  | 3 | 73.5 |  |  | 2 | Continuous infusion | Microbiological |  | 0.13 |  |  |  | Effective treatment |  |  |  |  |  |
| Bilgrami 2010 | Critical care patients | 10 |  | 6 | 57 | 70 |  | 3 | Intermittent bolus |  |  |  | 7.63 |  |  | All cause mortality |  |  |  |  |  |
| Binder 2013 | Critical care patients | 15 | 15 ICU patients | 10 | 59 | 82.3 |  | 3 | Intermittent bolus | Surrogate | 2.00 |  | 5.20 |  |  | Effective treatment |  |  |  |  |  |
| Binder 2013 | Cystic fibrosis children with acute pulmonary exacerbations | 10 | 10 with haematologic malignancies | 5 | 52 | 72 |  | 3 | Intermittent bolus | Surrogate | 2.00 |  | 1.10 |  |  | Effective treatment |  |  |  |  |  |
| Boonpeng 2022 | Critical care patients | 20 |  | 12 | 63 | 61.5 | 22.9 | 3 | Intermittent bolus | Microbiological |  | 0.02 |  |  | 55.00 | ICU mortality | Effective treatment |  |  |  |  |
| Chiriac 2023 | Critical care patients | 91 |  |  | 73 | 80 | 27.1 | 3 | Continuous infusion | Surrogate | 2.00 |  |  | 14.10 |  | In-hospital mortality | Duration of treatment |  |  |  |  |
| Cojutti 2015 | Paediatric patients who underwent haematopoietic stem cell transplantation | 21 |  | 13 | 9.6 | 36.1 |  | 3.3 | Continuous infusion | Surrogate | 2.00 |  |  | 29.80 |  | Effective treatment | Duration of treatment |  |  |  |  |
| Cojutti 2020 | Patients with febrile neutropenia | 75 |  | 47 | 58 | 77 |  | 3 | Continuous infusion | Surrogate | 2.00 |  |  | 12.70 |  | All cause mortality | Effective treatment | Duration of treatment |  |  |  |
| Cojutti 2021 | Critical care patients | 74 |  | 52 | 60.1 | 79 | 26 | 3 | Continuous infusion | Combination | 2.00 |  |  | 14.10 |  | Effective treatment | Duration of treatment |  |  |  |  |
| Del Bono 2016 | Critical care patients | 19 |  | 12 | 62 |  | 26 | 6 | Extended Infusion | Microbiological |  | 512.00 |  |  |  | 30-day mortality | Effective treatment |  |  |  |  |
| Drager 2023 | Critical care patients | 186 |  | 136 | 66 | 80 | 26 | 3 | Continuous infusion | Surrogate | 2.00 |  |  | 21.00 |  | 30-day mortality | Duration of treatment |  |  |  |  |
| Fournier 2018 | Burns patients | 10 | TDM | 8 | 48 |  |  | 3 | Intermittent bolus | Surrogate | 2.00 | 0.07 | 4.14 |  | 87.50 | All cause mortality |  |  |  |  |  |
| Fournier 2018 | Burns patients | 6 | Standard care | 6 | 31 |  |  | 3 | Intermittent bolus | Surrogate | 2.00 | 0.06 | 2.35 |  | 80.00 | All cause mortality |  |  |  |  |  |
| Gatti 2023 | Critical care patients | 24 |  | 15 | 68 | 67.5 | 24 | 0.5 | Continuous infusion | Microbiological |  | 0.12 |  | 19.90 |  | 30-day mortality | Effective treatment | Duration of treatment | Emergence of AMR |  |  |
| Gatti 2024 | Critical care patients with BSIs or VAP | 32 |  | 24 | 71.5 | 80 | 27.6 | 6 | Continuous infusion | Microbiological |  | 0.12 |  | 14.90 |  | 30-day mortality | ICU mortality | Effective treatment | Emergence of AMR |  |  |
| Guilhaumou 2023 | Critical care patients | 30 |  | 18 | 61 | 74 | 25.39 | 3 | Continuous infusion | Surrogate | 2.00 |  |  | 18.00 |  | 30-day mortality | Length of stay on ICU | Duration of treatment |  |  |  |
| Hatti 2018 | Elderly patients | 12 |  | 7 | 75 |  | 22.9 | 1.5 | Extended Infusion | Surrogate | 2.00 |  | 2.38 |  |  | 30-day mortality | In-hospital stay | Readmission |  |  |  |
| Heil 2018 | Critical care patients | 20 |  | 10 | 55.5 | 89.7 | 33.4 | 3 | Extended Infusion | Microbiological |  | 0.25 | 5.50 |  | 100.00 | 30-day mortality | In-hospital stay | Length of stay on ICU | Effective treatment |  |  |
| Kitzes-Cohen 2002 | Critical care patients | 8 | Group I: Patients with CrCl higher than 50 ml/min | 5 | 73.6 |  |  | 3 | Intermittent bolus | Microbiological |  | 1.25 | 3.30 |  | 50.00 | All cause mortality |  |  |  |  |  |
| Kitzes-Cohen 2002 | Critical care patients | 6 | Group II: Patients with CrCl lower than 50 ml/min | 4 | 72.8 |  |  | 2 | Intermittent bolus | Microbiological |  | 0.41 | 3.40 |  | 66.70 | All cause mortality |  |  |  |  |  |
| Kuti 2018 | Cystic fibrosis children with acute pulmonary exacerbations | 30 |  | 6 | 14 | 46.2 |  | 1.8 | Extended Infusion | Microbiological |  | 1.50 |  |  |  | Duration of treatment |  |  |  |  |  |
| Luque 2021 | Patients with low body weight | 18 | Low Body Weight | 10 | 61.5 | 45 | 17.3 | 3.9 | Continuous infusion | Combination | 2.00 |  |  | 19.90 |  | Effective treatment |  |  |  |  |  |
| Luque 2021 | Critical care patients | 18 | Normal Body Weight | 10 | 56.5 | 84 | 29.8 | 7.4 | Continuous infusion | Combination | 2.00 |  |  | 22.40 |  | Effective treatment |  |  |  |  |  |
| Maimongkol 2022 | Critical care paediatric patients | 54 | Extended infusion | 25 | 1 | 8.8 |  | 0.9 | Extended Infusion | Surrogate | 2.00 |  | 2.30 |  | 51.00 | 30-day mortality | In-hospital stay | Toxicity / adverse events | Duration of treatment |  |  |
| Maimongkol 2022 | Critical care paediatric patients | 18 | Intermittent bolus | 7 | 0.92 | 5.2 |  | 0.6 | Intermittent bolus | Surrogate | 2.00 |  | 0.80 |  | 28.00 | 30-day mortality | In-hospital stay | Toxicity / adverse events | Duration of treatment |  |  |
| McDonald 2016 | Critical care patients | 22 | Licensed dose | 10 | 49.2 |  | 27.2 | 3.6 | Intermittent bolus | Combination |  |  |  |  | 45.50 | In-hospital mortality | In-hospital stay | Length of stay on ICU | Effective treatment | Toxicity / adverse events | Duration of treatment |
| McDonald 2016 | Critical care patients | 25 | High dose | 12 | 44 |  | 27.6 | 5.1 | Intermittent bolus | Combination |  |  |  |  | 53.60 | In-hospital mortality | In-hospital stay | Length of stay on ICU | Effective treatment | Toxicity / adverse events | Duration of treatment |
| Morita 2014 | General hospital patients | 5 |  | 2 | 64 |  |  | 6 | Intermittent bolus | Microbiological |  | 0.06 | 2.23 |  | 100.00 | All cause mortality | Effective treatment | Toxicity / adverse events | Duration of treatment |  |  |
| Nurlu Temel 2023 | Elderly patients | 59 |  | 40 | 73.2 |  |  | 1 | Extended Infusion | Surrogate | 8.00 |  | < 8 mg/L |  | 20.30 | All cause mortality | Effective treatment | Duration of treatment |  |  |  |
| Paice 2024 | Critical care paediatric patients | 29 |  |  | 4 | 16.3 |  | 0.978 | Intermittent bolus | Surrogate | 1.00 |  |  |  |  | 30-day mortality |  |  |  |  |  |
| Pea 2017 | Critical care patients | 30 |  | 21 | 62.5 | 69.5 |  | 4.8 | Continuous infusion | Microbiological |  | 32.00 |  | 47.75 |  | Effective treatment | Toxicity / adverse events | Duration of treatment |  |  |  |
| Petersson 2021 | Critical care patients | 98 |  | 30 | 62 | 83.7 |  | 1 | Intermittent bolus | Surrogate | 2.00 |  | 8.00 |  |  | ICU mortality | In-hospital mortality |  |  |  |  |
| Ragonnet 2024 | Critical care paediatric patients | 103 | Intermittent infusion | 62 | 4.7 | 12.8 |  | 4.6 | Intermittent bolus | Surrogate | 2.00 |  |  |  |  | ICU mortality |  |  |  |  |  |
| Ragonnet 2024 | Critical care paediatric patients | 71 | Continuous infusions | 40 | 4.3 | 13.3 |  | 4.8 | Continuous infusion | Surrogate | 2.00 |  |  |  |  | ICU mortality |  |  |  |  |  |
| Razzazzadeh 2022 | General hospital patients | 15 | 1 g, 3-h infusion | 10 | 44.4 | 64 |  | 3 | Extended Infusion | Microbiological |  | 6.25 | 0.94 |  |  | All cause mortality | Length of stay on ICU |  |  |  |  |
| Razzazzadeh 2022 | General hospital patients | 15 | 2 g, 3-h infusion | 9 | 40.07 | 67 |  | 6 | Extended Infusion | Microbiological |  | 6.50 | 1.56 |  |  | All cause mortality | Length of stay on ICU |  |  |  |  |
| Razzazzadeh 2022 | General hospital patients | 15 | 1 g, 6-h infusion | 10 | 35.87 | 62.33 |  | 3 | Extended Infusion | Microbiological |  | 7.00 | 4.24 |  |  | All cause mortality | Length of stay on ICU |  |  |  |  |
| Sanz Codina 2022 | Critical care patients | 43 |  | 26 | 59 |  | 30.7 |  | Continuous infusion | Microbiological |  |  |  | 22.40 |  | 30-day mortality | In-hospital mortality |  |  |  |  |
| Schmid 2023 | Critical care patients | 25 |  | 17 | 55.4 |  |  | 3 | Continuous infusion | Combination | 2.00 | 0.13 |  | 14.90 |  | ICU mortality |  |  |  |  |  |
| Taccone 2021 | Transplant patients | 29 |  | 14 | 51 | 59 | 22.4 | 3 | Intermittent bolus | Surrogate | 2.00 |  | 4.60 |  |  | ICU mortality | In-hospital mortality | Length of stay on ICU |  |  |  |
| Tseng 2025 | Patients with low body weight (LBW) | 20 |  | 11 | 65.02 | 41.8 |  | 2.6 | Intermittent bolus | Surrogate | 2.00 |  | 8.76 |  | 70.00 | 30-day mortality | Toxicity / adverse events |  |  |  |  |
| Venugopalan 2018 | General hospital patients | 22 |  | 16 | 44 | 69 | 24 | 6 | Continuous infusion | Combination | 2.00 | 2.00 |  | 17.80 | 95.00 | 30-day mortality | Effective treatment | Duration of treatment |  |  |  |
| Wang 2016 | Critical care patients | 104 | Continuous infusion | 63 | 60.4 |  |  | 2.4 | Continuous infusion |  |  |  | 9.00 |  |  | 30-day mortality | Length of stay on ICU | Effective treatment | Toxicity / adverse events | Duration of treatment |  |
| Wang 2016 | Critical care patients | 108 | Intermittent bolus | 70 | 57.8 |  |  | 3 | Intermittent bolus |  |  |  | 1.00 |  |  | 30-day mortality | Length of stay on ICU | Effective treatment | Toxicity / adverse events | Duration of treatment |  |
| Wang 2022 | Critical care paediatric patients | 14 | ƒT>MIC ≥5.6 h | 10 | 2.17 | 12.67 |  | 0.75 | Intermittent bolus | Surrogate | 1.00 |  |  |  |  | In-hospital stay | Effective treatment | Toxicity / adverse events | Duration of treatment |  |  |
| Wang 2022 | Critical care paediatric patients | 39 | ƒT>MIC <5.6 h | 21 | 2.17 | 12.67 |  | 0.75 | Intermittent bolus | Surrogate | 1.00 |  |  |  |  | In-hospital stay | Effective treatment | Toxicity / adverse events | Duration of treatment |  |  |
| You 2024 | Critical care patients | 99 |  | 65 | 56 |  |  | 3 | Intermittent bolus | Combination | 1.00 | 14.83 | 7.00 |  | 38.40 | Length of stay on ICU | Effective treatment |  |  |  |  |
| Zhang 2017 | Post-neurosurgical meningitis patients | 42 | 1g q8h | 29 | 42.4 | 64.8 | 22.7 | 3 | Intermittent bolus |  |  |  | 0.30 |  |  | Effective treatment |  |  |  |  |  |
| Zhang 2017 | Post-neurosurgical meningitis patients | 19 | 1g q6h | 8 | 44.5 | 63.4 | 22.7 | 4 | Intermittent bolus |  |  |  | 0.40 |  |  | Effective treatment |  |  |  |  |  |
| Zhang 2017 | Post-neurosurgical meningitis patients | 21 | 2g q8h | 12 | 44.3 | 67.7 | 24.3 | 6 | Extended Infusion |  |  |  | 0.50 |  |  | Effective treatment |  |  |  |  |  |
| Zhao 2017 | Critical care patients | 25 | Intermittent bolus | 11 | 67 | 63.8 |  | 3 | Intermittent bolus | Microbiological |  | 0.25 | 0.60 |  |  | ICU mortality | Length of stay on ICU | Effective treatment | Duration of treatment |  |  |
| Zhao 2017 | Critical care patients | 25 | Continuous infusion | 10 | 68 | 60.5 |  | 3 | Continuous infusion | Microbiological |  | 0.25 | 11.40 |  |  | ICU mortality | Length of stay on ICU | Effective treatment | Duration of treatment |  |  |
| Zhao 2022 | Critical care patients | 39 | Treatment success | 30 | 64.9 | 61.1 |  | 3 | Extended Infusion | Microbiological |  | 2.00 | 8.53 |  |  | Effective treatment |  |  |  |  |  |
| Zhao 2022 | Critical care patients | 25 | Treatment Failure | 17 | 64 | 64 |  | 3 | Extended Infusion | Microbiological |  | 8.00 | 10.07 |  |  | Effective treatment |  |  |  |  |  |
| Zhou 2011 | General hospital patients | 45 |  | 29 | 76.2 | 62.2 |  | 3 | Intermittent bolus | Microbiological |  | 4.00 |  |  |  | Effective treatment |  |  |  |  |  |

Supplementary material 2: a-k) Forest plots of per study effect estimates for each clinical outcome assessed.

1.
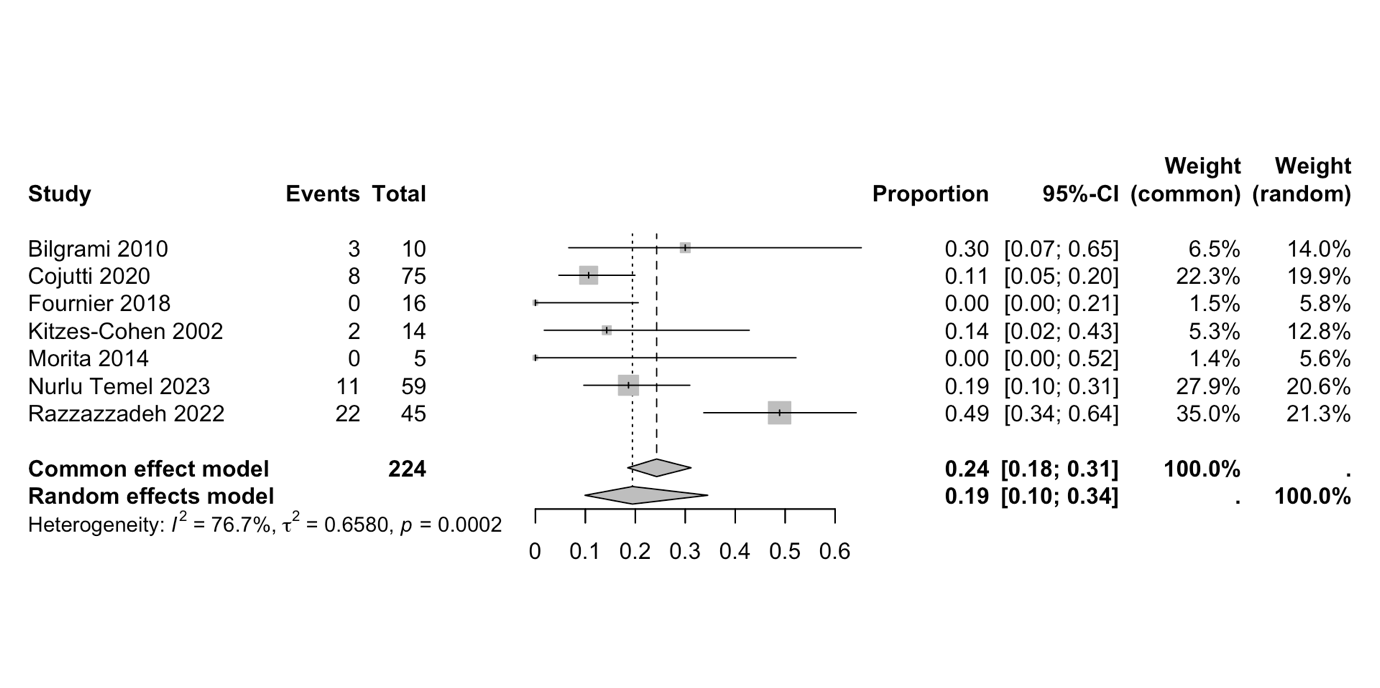
All-cause mortality
2.
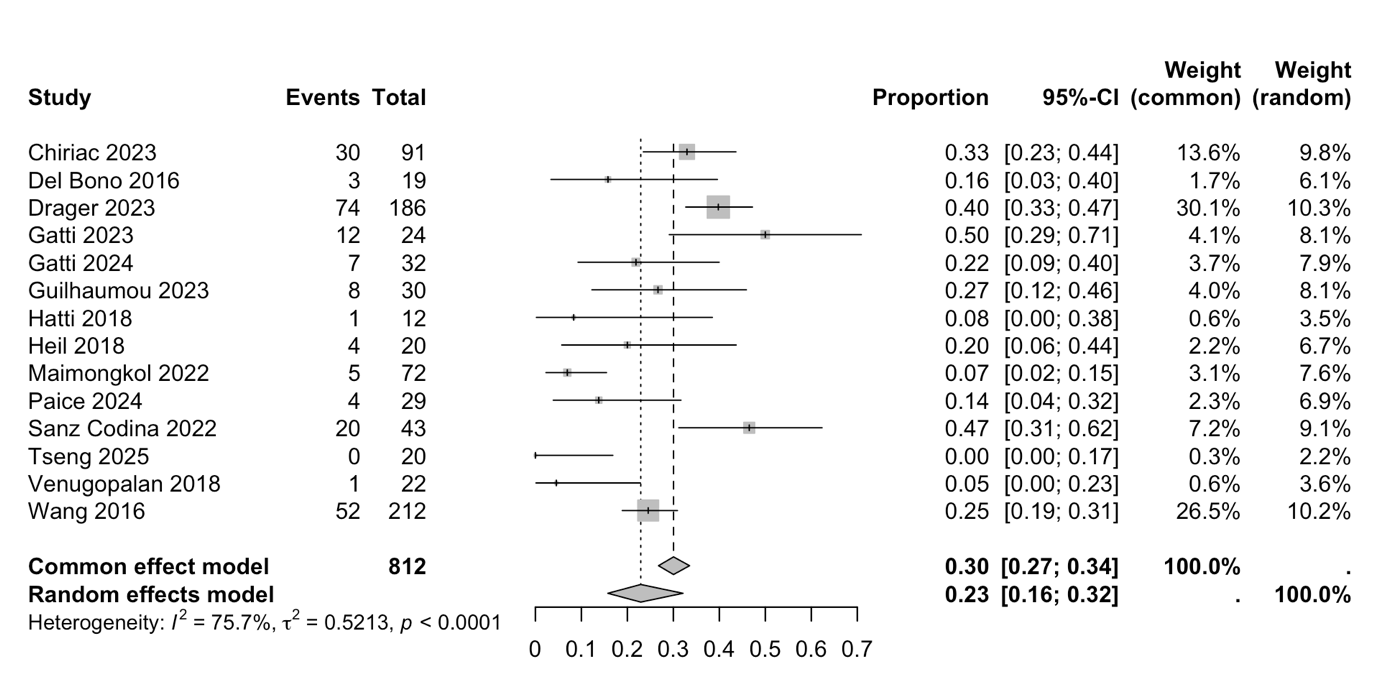
30-day mortality
3. ICU mortality


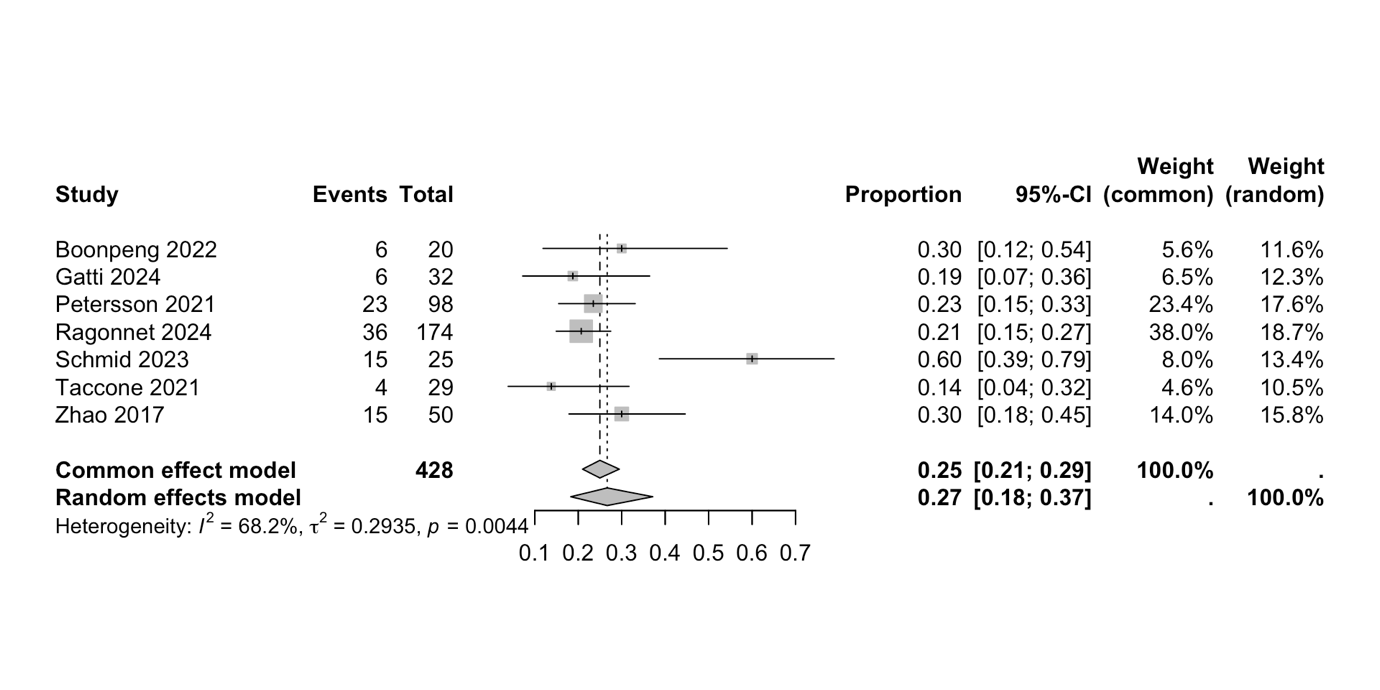


1. Hospital mortality


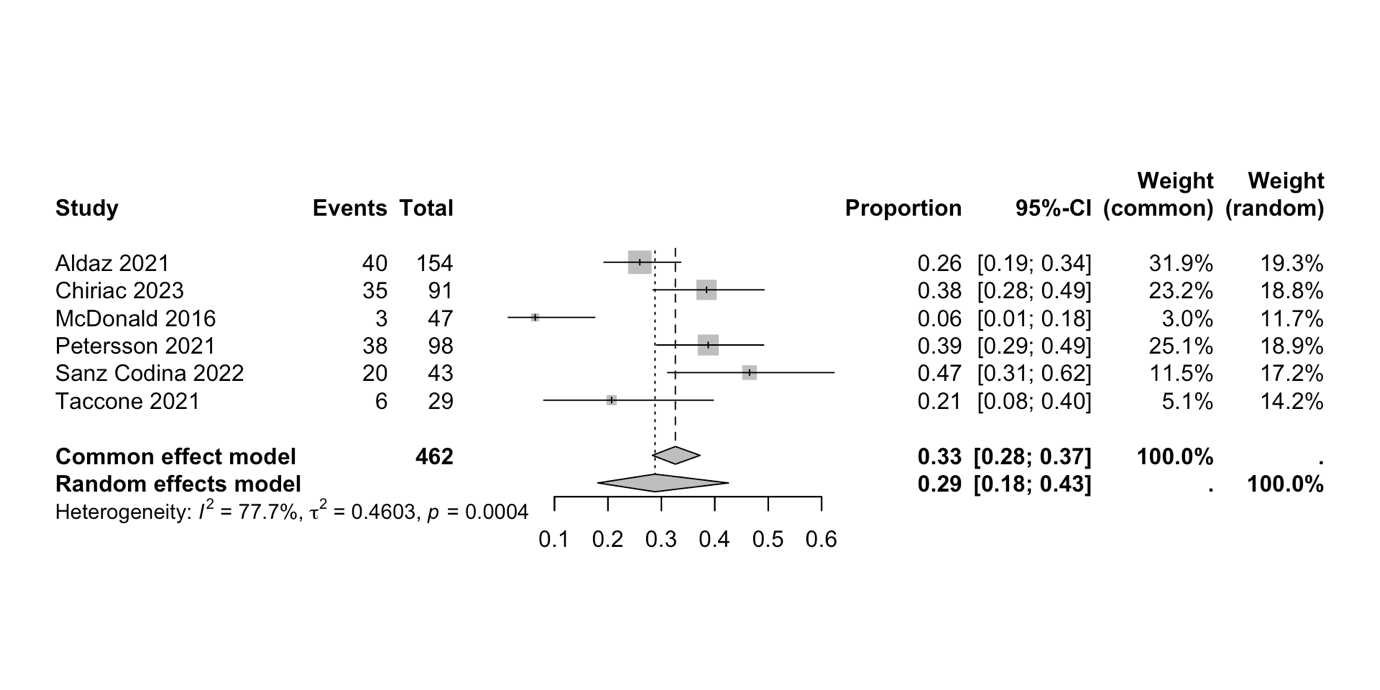


1.
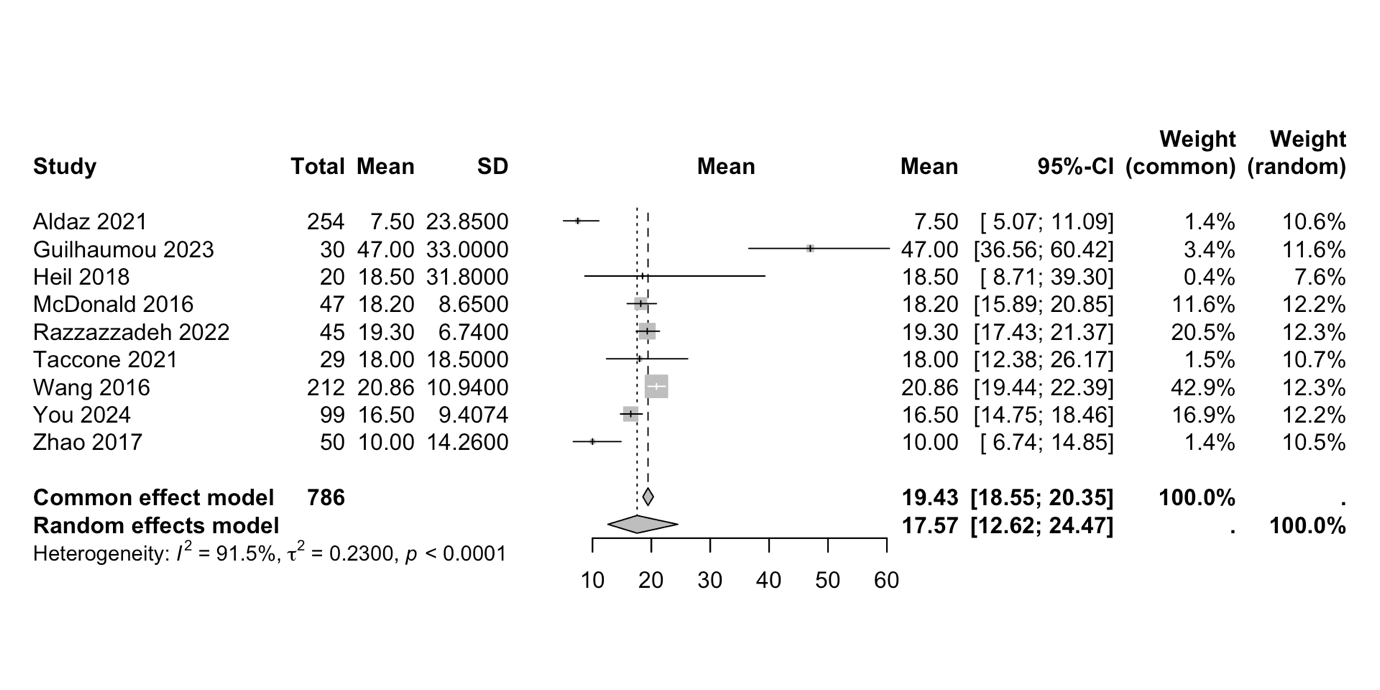
Length of stay on ICU
2. In-hospital stay


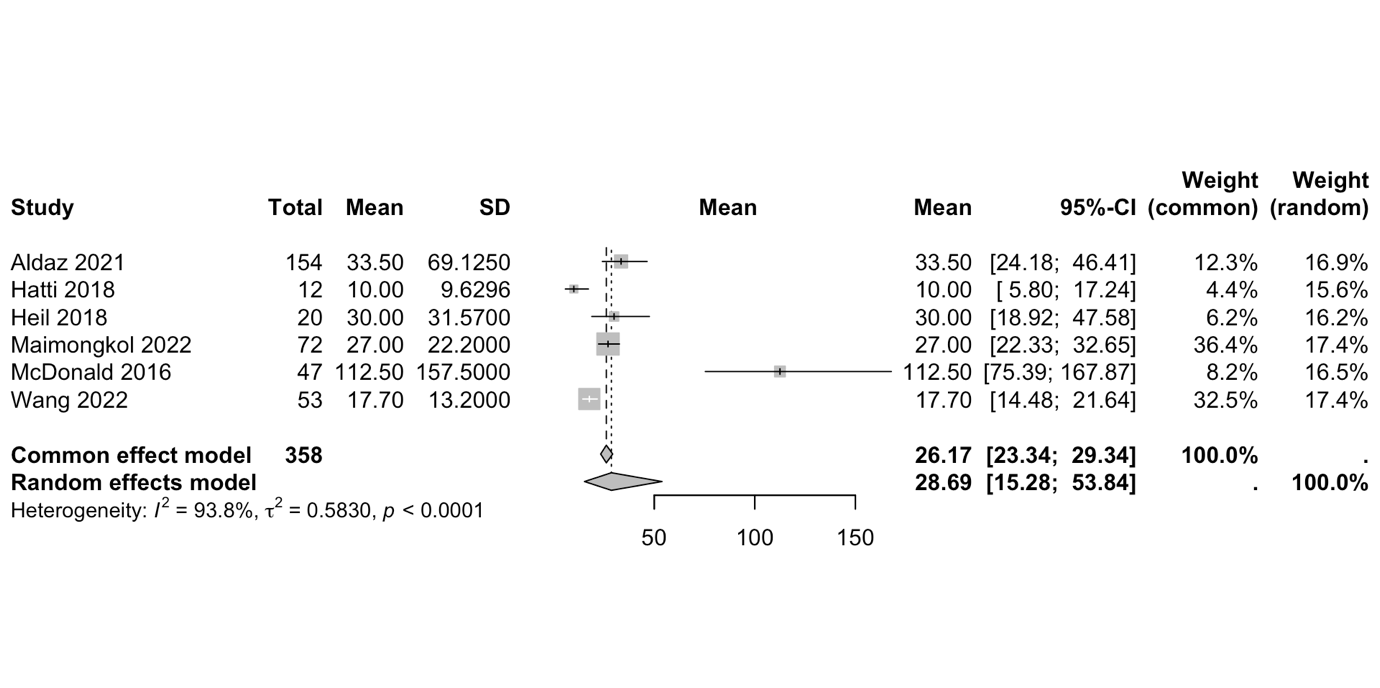


1. Clinical cure


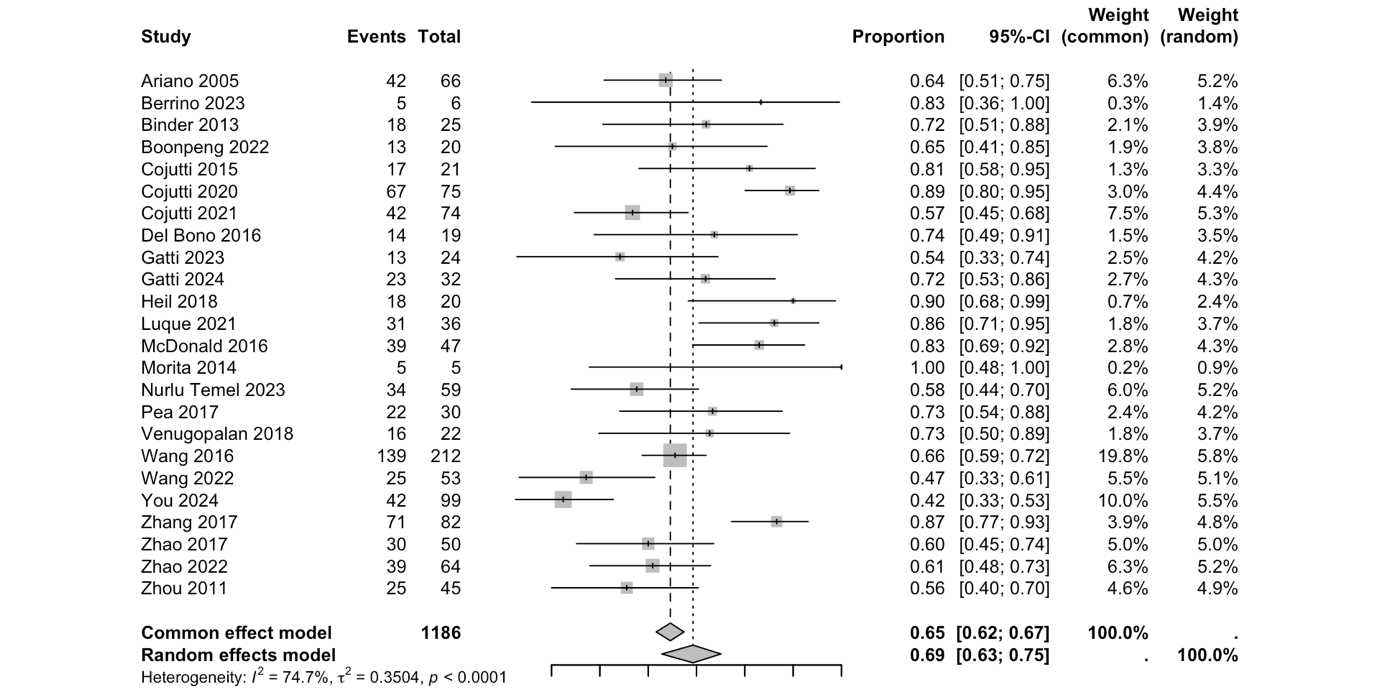


1. Toxicity / adverse events


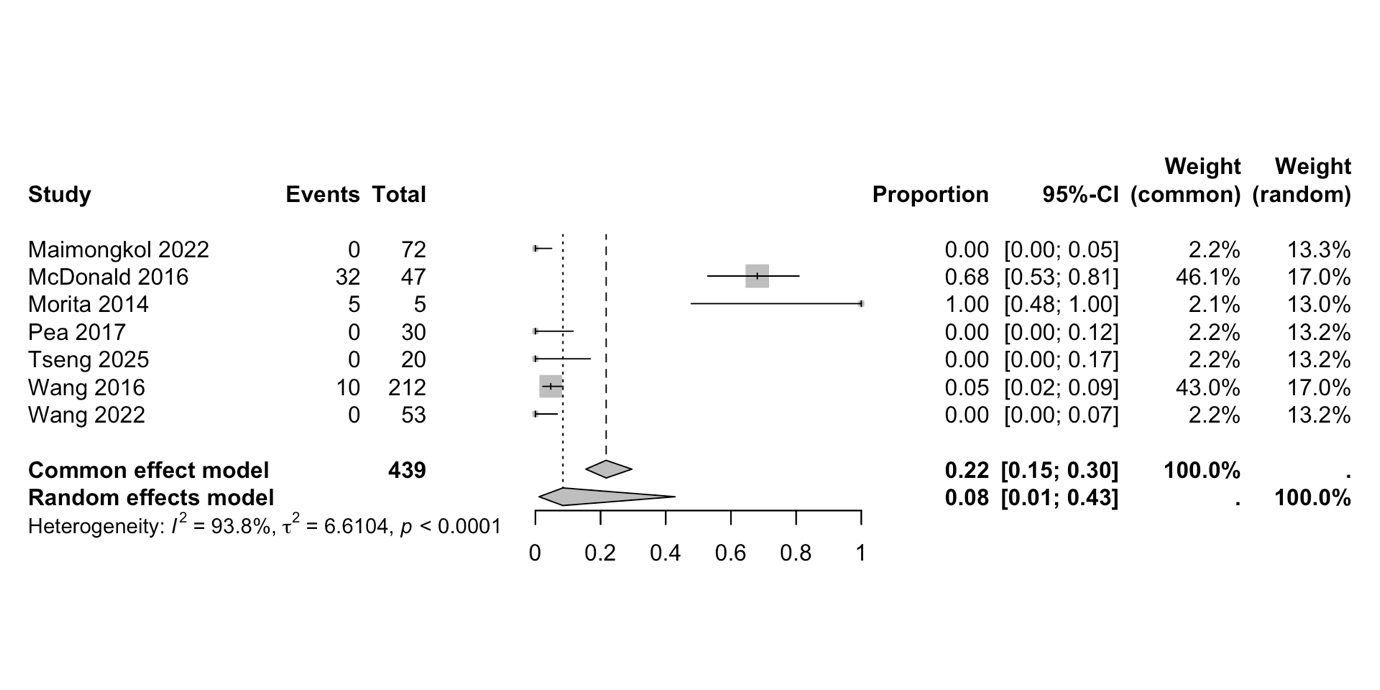


1.
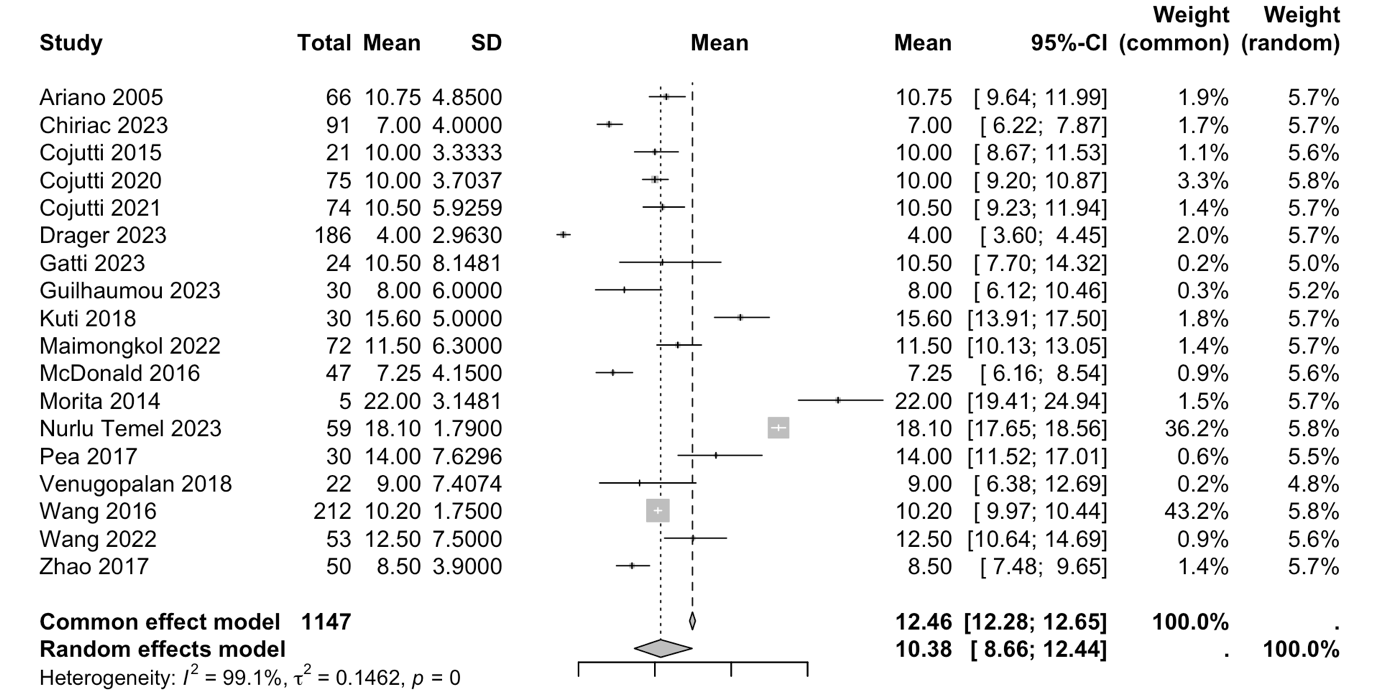
Duration of treatment
2. Readmission


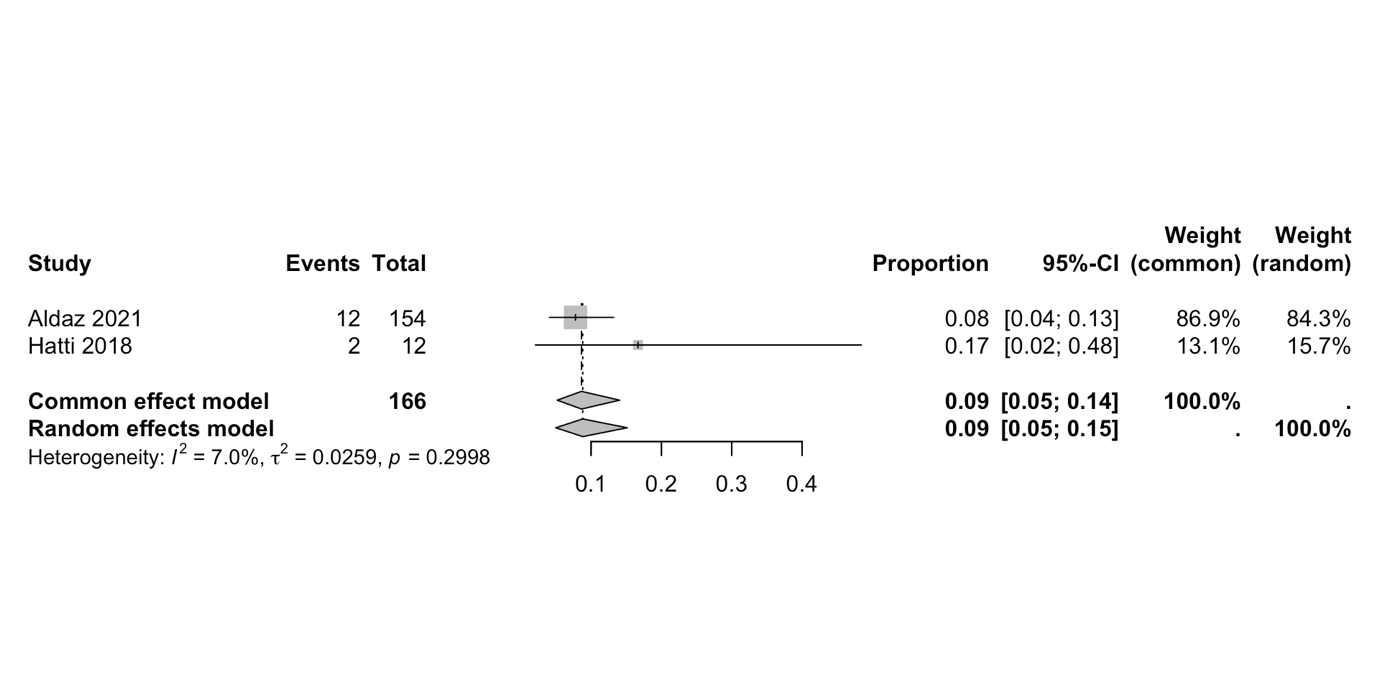


1. Emergence of AMR


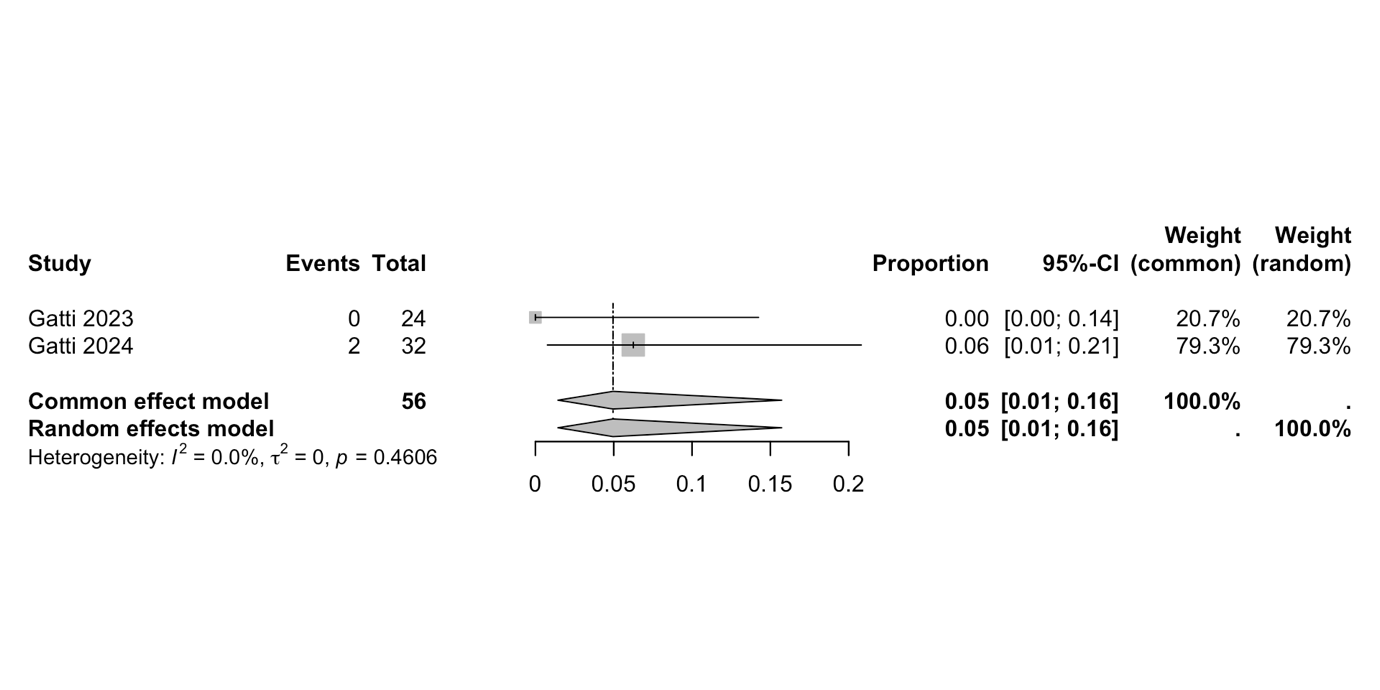


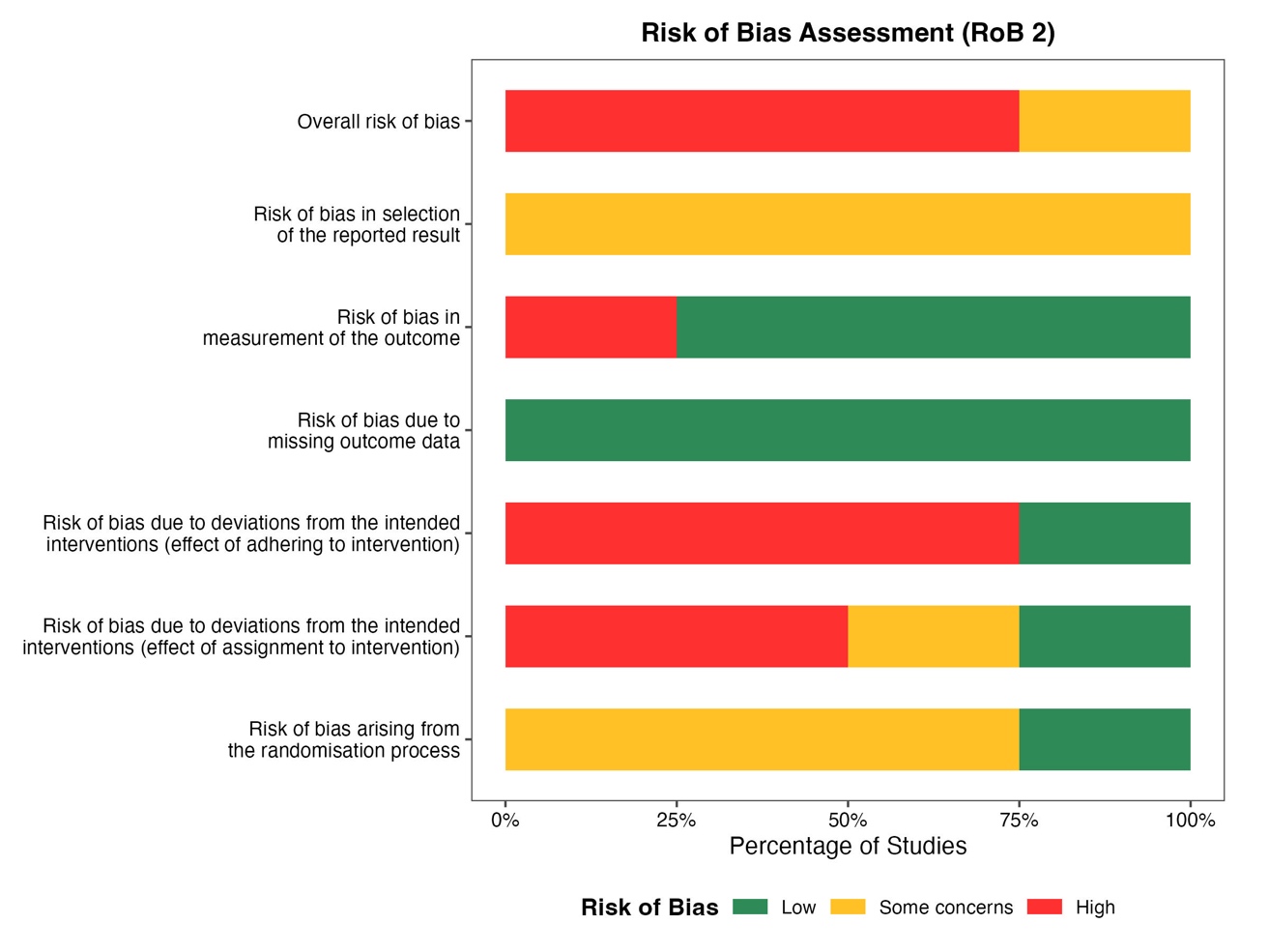


Figure S1: Quality assessment of included randomised controlled trials using the Risk of Bias in Randomised Trials (RoB 2) tool.


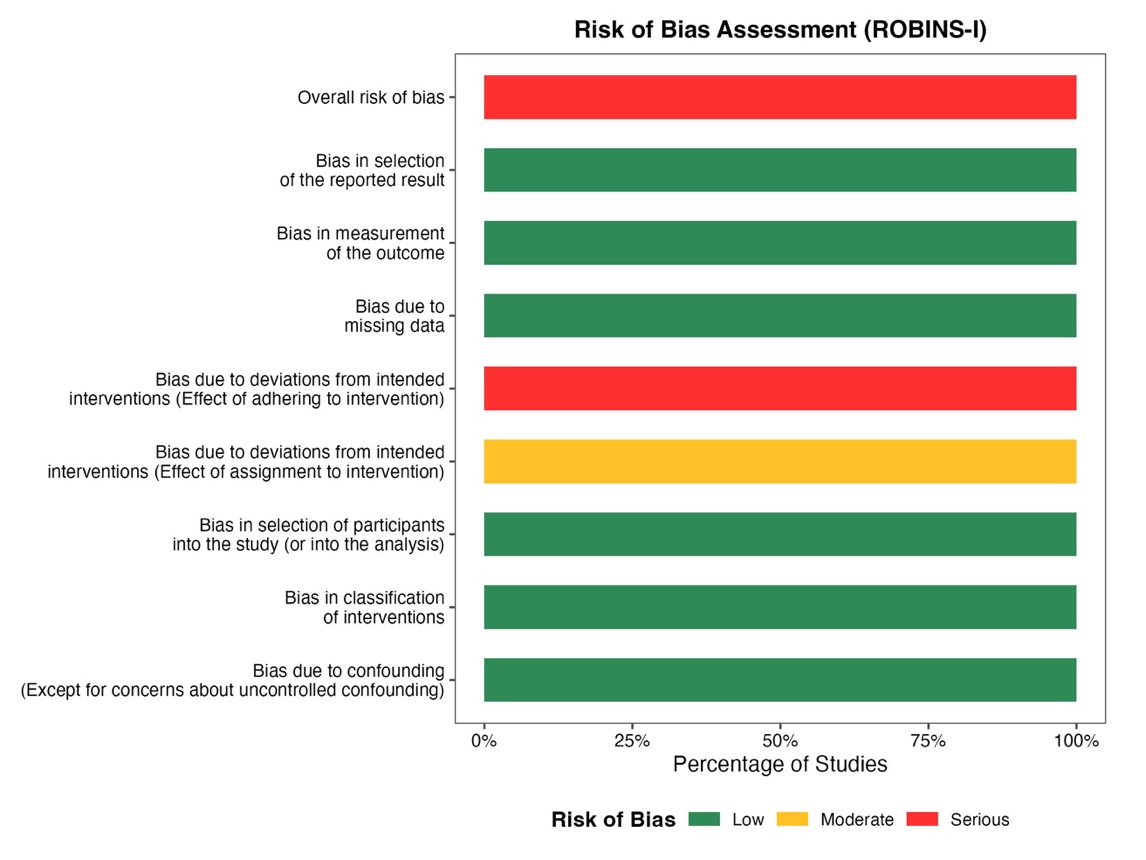


Figure S2: Quality assessment of non-randomised trials using the Risk of Bias in Non-randomised Intervention Studies (ROBINS-I) tool.


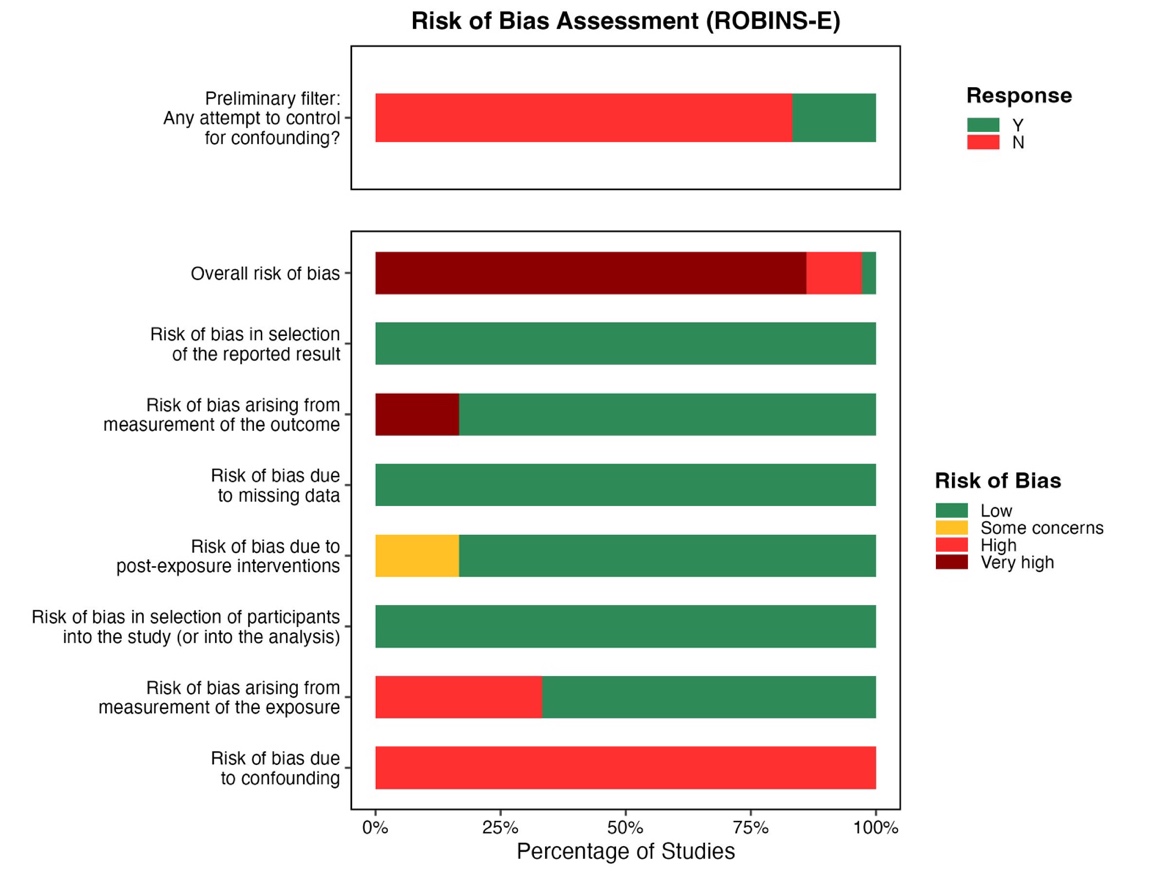


Figure S3: Quality assessment of observational or exposure studies using the Risk of Bias in Non-Interventional Studies – Exposure (ROBINS-E) tool.

Table S1 – Baseline demographics of cohorts reporting a clinical cure outcome.

| **Cohort** | **Patient Group** | **n** | **Administration** | **Male n (%)** | **Age** | **Weight (kg)** | **BMI (kg/m^2)^** | **APACHE II** | **SOFA** | **CrCl (mL/min)** | **eGFR (mL/min)** | **Serum Creatinine (mg/dL)** | **MIC source** | **Surrogate MIC (mg/L)** | **Micro MIC (mg/L)** | **Conc (mg/L)** | **100% ƒT>MIC (%)** | **Cure** |
| --- | --- | --- | --- | --- | --- | --- | --- | --- | --- | --- | --- | --- | --- | --- | --- | --- | --- | --- |
| Ariano 2005 a | Febrile neutropenia | 44 | Intermittent bolus | 24 (55) | 32.0 (13.0) | 62.5 (12.6) | - | - | - | - | 111.0 (32.0) | 0.74 (0.20) | Surrogate | 0.13 | 0.5 | 0.02 |  | 42 (95.5) |
| Ariano 2005 b | Febrile neutropenia | 22 | Intermittent bolus | 10 (45) | 46.0 (16.0) | 62.5 (9.8) | - | - | - | - | 102.0 (37.0) | 0.70 (0.18) | Surrogate | 0.5 | 3.2 | 0.02 |  | 0 (0) |
| Berrino 2023 | G- BSIs | 6 | Continuous infusion | 3 (50) | 73.5 (3.5) | - | - | - | - | - | 90.5 (38.2) | - | Microbiological | - | 0.125 | - |  | 5 (83.3) |
| Binder 2013 a | Critically ill | 15 | Intermittent bolus | 10 (66) | 59.0 (38.5) | 82.3 (61.9) | - | - | - | - | - | - | Surrogate | 2 | - | 5.2 |  | 14 (93.3) |
| Binder 2013 b | Cystic fibrosis children | 10 | Intermittent bolus | 5 (50) | 52.0 (29.6) | 72.0 (27.4) | - | - | - | - | - | - | Surrogate | 2 | - | 1.1 |  | 4 (40.0) |
| Boonpeng 2022 | Critically ill | 20 | Intermittent bolus | 12 (60) | 63.0 (19.3) | 61.5 (12.1) | 22.9 (3.6) | 20.0 (6.7) | 8.0 (3.7) | 44.6 (41.9) | - | - | Microbiological | - | 0.023 | - | 55 | 13 (65.0) |
| Cojutti 2015 | Paediatric patients | 21 | Continuous infusion | 13 (62) | 9.6 (5.4) | 36.1 (20.5) | - | - | - | - | 189.5 (109.0) | - | Surrogate | 2 | - | 29.8 |  | 17 (81.0) |
| Cojutti 2020 | Febrile neutropenia | 75 | Continuous infusion | 47 (63) | 58.0 (1.1) | 77.0 (17.4) | - | - | - | - | 113.3 (43.4) | - | Surrogate | 2 | - | 12.7 |  | 67 (89.3) |
| Cojutti 2021 | Critically ill | 74 | Continuous infusion | 52 (70) | 60.1 (15.0) | 79.0 (21.0) | 26.0 (4.3) | - | 7.0 (1.5) | - | 91.5 (63.7) | - | Combination | 2 | - | 14.1 |  | 42 (56.8) |
| Del Bono 2016 | Critically ill | 19 | Extended Infusion | 12 (63) | 62.0 (13.3) | - | 26 (10.0) | 11 (3.7) |  | - | - | 0.9 (0.52) | Microbiological | - | 512 | - |  | 14 (73.7) |
| Gatti 2023 | Critically ill | 24 | Continuous infusion | 15 (62.5) | 68.0 (13.0) | 67.5 (14.8) | 24 (4.2) | - | 14 (4.6) | - | - | - | Microbiological | - | 0.12 | 19.9 |  | 13 (54.2) |
| Gatti 2024 | Critically ill with BSIs or VAP | 32 | Continuous infusion | 24 (75) | 71.5 (11.1) | 80 (14.8) | 27.6 (6.2) | 19.5 (6.4) | 9.0 (5.4) | - | - | - | Microbiological | - | 0.12 | 14.9 |  | 23 (71.9) |
| Heil 2018 | Critically ill | 20 | Extended Infusion | 10 (50) | 55.5 (16.7) | 89.7 (38.2) | 33.4 (14) | 15 (6.0) | 4 (2.0) | 72.9 (36.2) | - | 0.74 (1.0) | Microbiological | - | 0.25 | 5.5 | 100 | 18 (90) |
| Luque 2021 a | Critically ill | 18 | Continuous infusion | 10 (56) | 61.5 (22.8) | 45.0 (9.2) | 17.3 (1.6) | 15.5 (10.0) | - | - | 92.0 (77.0) | - | Combination | 2 | - | 19.9 |  | 16 (88.9) |
| Luque 2021 b | Critically ill | 18 | Continuous infusion | 10 (56) | 56.5 (11.8) | 84.0 (37.0) | 29.8 (17.2) | 19.0 (17.0) | - | - | 92.0 (72.0) | - | Combination | 2 | - | 22.4 |  | 15 (83.3) |
| McDonald 2016 a | Critically ill | 22 | Intermittent bolus | 10 (46) | 49.2 (14.2) | - | 27.2 (11.2) | - | 6.3 (3.6) | 128.4 (19.9) | - | - | Combination | - | - | - | 45.5 | 18 (81.8) |
| McDonald 2016 b | Critically ill | 25 | Intermittent bolus | 12 (48) | 44.0 (15.3) | - | 27.6 (6.7) | - | 5.0 (3.6) | 234.2 (94.6) | - | - | Combination | - | - | - | 53.6 | 21 (84.0) |
| Morita 2014 | General hospital | 5 | Intermittent bolus | 2 (40) | 64.0 (7.0) | - | - | - | - | - | - | - | Microbiological | - | 0.06 | 2.23 | 100 | 5 (100.0) |
| Nurlu Temel 2023 | Elderly patients | 59 | Extended Infusion | 40 (68) | 73.2 (8.8) | - | - | - | - | - | - | - | Surrogate | 8 | - | - | 20.3 | 34 (57.6) |
| Pea 2017 | Critically ill | 30 | Continuous infusion | 21 (70) | 62.5 (14.6) | 69.5 (13.7) | - | - | - | - | - | - | Microbiological | - | 32 | 47.75 |  | 22 (73.3) |
| Venugopalan 2018 | General hospital | 22 | Continuous infusion | 16 (73) | 44.0 (26.7) | 69.0 (12.6) | 24.0 (7.4) | - | - | - | - | 0.70 (0.22) | Combination | 2 | 2 | 17.8 | 95 | 16 (72.7) |
| Wang 2016 a | Critically ill | 104 | Continuous infusion | 63 (61) | 60.4 (14.9) | - | - | 19.5 (6.4) | 5.9 (2.7) | - | - | - | Combination | 2 | - | 9 |  | 81 (77.9) |
| Wang 2016 b | Critically ill | 108 | Intermittent bolus | 70 (65) | 57.8 (15.3) | - | - | 18.8 (6.7) | 6.2 (2.9) | - | - | - | Surrogate | 2 | - | 1 |  | 58 (53.7) |
| Wang 2022 a | Critical care paediatric | 14 | Intermittent bolus | 10 (71) | 2.2 (3.2) | 12.7 (11.1) | - | - | - | - | 129.6 (75.7) | - | Surrogate | 1 | - | - |  | 11 (78.6) |
| Wang 2022 b | Critical care paediatric | 39 | Intermittent bolus | 21 (54) | 2.2 (3.2) | 12.7 (11.1) | - | - | - | - | 177.3 (66.1) | - | Surrogate | 1 | - | - |  | 14 (35.9) |
| You 2024 | Critically ill | 99 | Intermittent bolus | 65 (66) | 56.0 (18.0) | - | - | 27.5 (8.9) | - | - | 57.1 (56.6) | 1.05 (0.89) | Combination | 1 | 14.8 | 7 | 38.4 | 42 (42.4) |
| Zhang 2017 a | Post-neurosurgery meningitis | 42 | Intermittent bolus | 29 (69) | 42.4 (13.2) | 64.8 (10.2) | 22.7 (3.2) | - | - | 117.1 (40.1) | - | - | - | - | - | 0.3 |  | 37 (88.1) |
| Zhang 2017 b | Post-neurosurgery meningitis | 19 | Intermittent bolus | 8 (42) | 44.5 (13.8) | 63.4 (12.9) | 22.7 (3.2) | - | - | 125.1 (51.9) | - | - | - | - | - | 0.4 |  | 18 (94.7) |
| Zhang 2017 c | Post-neurosurgery meningitis | 21 | Extended Infusion | 12 (57) | 44.3 (12.4) | 67.7 (13.0) | 24.3 (4.0) | - | - | 182.8 (103.0) | - | - | - | - | - | 0.5 |  | 16 (76.2) |
| Zhao 2017 a | Critically ill | 25 | Intermittent bolus | 11 (44) | 67.0 (12.2) | 63.8 (11.8) | - | 19.7 (5.9) | - | 91.1 (34.0) | - | - | Microbiological | - | 0.25 | 0.6 |  | 14 (56.0) |
| Zhao 2017 b | Critically ill | 25 | Continuous infusion | 10 (40) | 68.0 (15.4) | 60.5 (10.2) | - | 19.4 (5.0) | - | 97.5 (43.4) | - | - | Microbiological | - | 0.25 | 11.4 |  | 16 (64.0) |
| Zhao 2022 a | Critically ill | 39 | Extended Infusion | 30 (77) | 64.9 (15.2) | 61.1 (18.0) | - | 17.0 (8.2) | - | - | - | 0.67 (0.74) | Microbiological | - | 2 | 8.53 |  | 39 (100.0) |
| Zhao 2022 b | Critically ill | 25 | Extended Infusion | 17 (68) | 64.0 (14.8) | 64.0 (9.6) | - | 16.0 (7.7) | - | - | - | 0.81 (0.83) | Microbiological | - | 8 | 10.07 |  | 0 (0) |
| Zhou 2011 | General hospital | 45 | Intermittent bolus | 29 (64) | 76.2 (8.3) | 62.2 (11.0) | - | 14.6 (4.5) | - | 50.3 (72.8) | - | 0.89 (3.00) | Microbiological | - | 4 | - |  | 25 (55.6) |

Abbreviations: APACHE II = Acute Physiology and Chronic Health Evaluation II score; BMI = body mass index; BSI(s) = bloodstream infection(s); Conc = meropenem concentration (trough [Cmin] for intermittent or extended dosing, or steady-state [Css] for continuous infusion); CrCl = creatinine clearance; eGFR = estimated glomerular filtration rate; ƒT>MIC = proportion of the dosing interval that free (unbound) meropenem concentration exceeds the MIC; G− = Gram-negative; MIC = minimum inhibitory concentration; Micro MIC = microbiologically determined MIC; n = number of patients; SOFA = Sequential Organ Failure Assessment score; Surrogate MIC = assumed MIC based on a clinical breakpoint; VAP = ventilator-associated pneumonia.

Figure S4 - a) Leave-one-out sensitivity analysis of clinical cure (effective treatment) meta-analysis and impact on pooled proportion. Red dotted line – pooled proportion when all studies included. Blue points and bars - Point estimates and 95% CI. b) Funnel plot with 95% pseudo-confidence intervals. Dots represent individual cohorts.


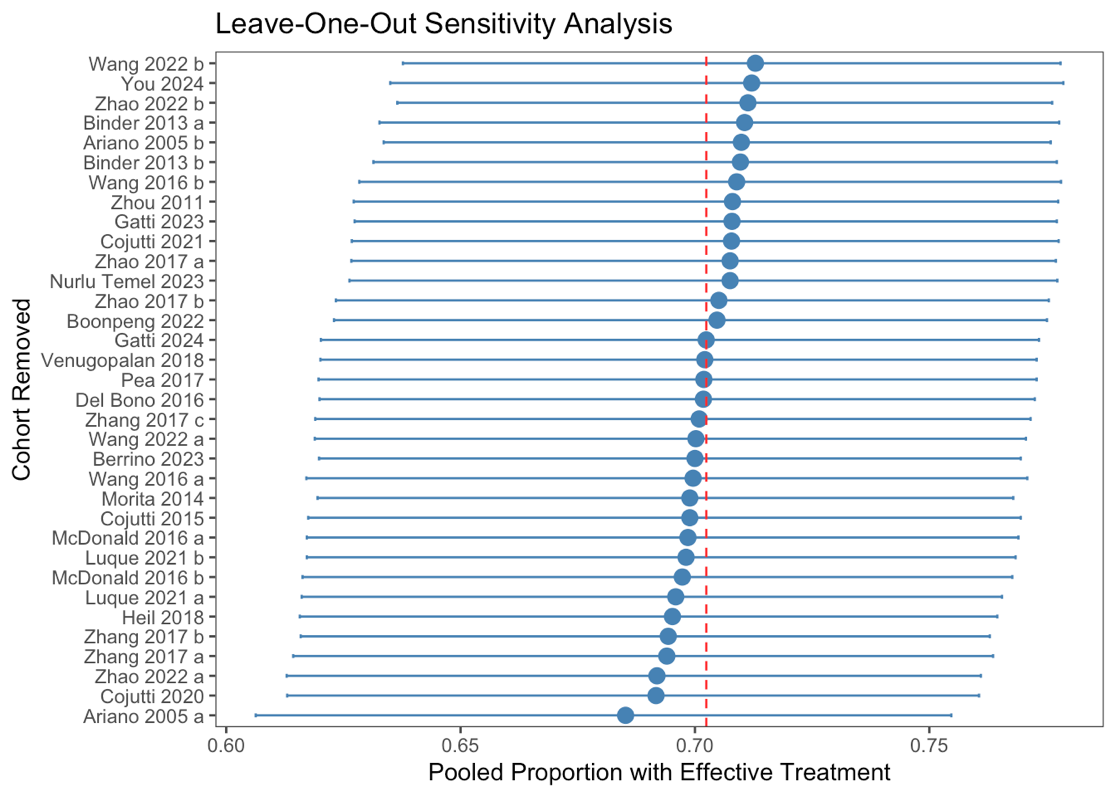

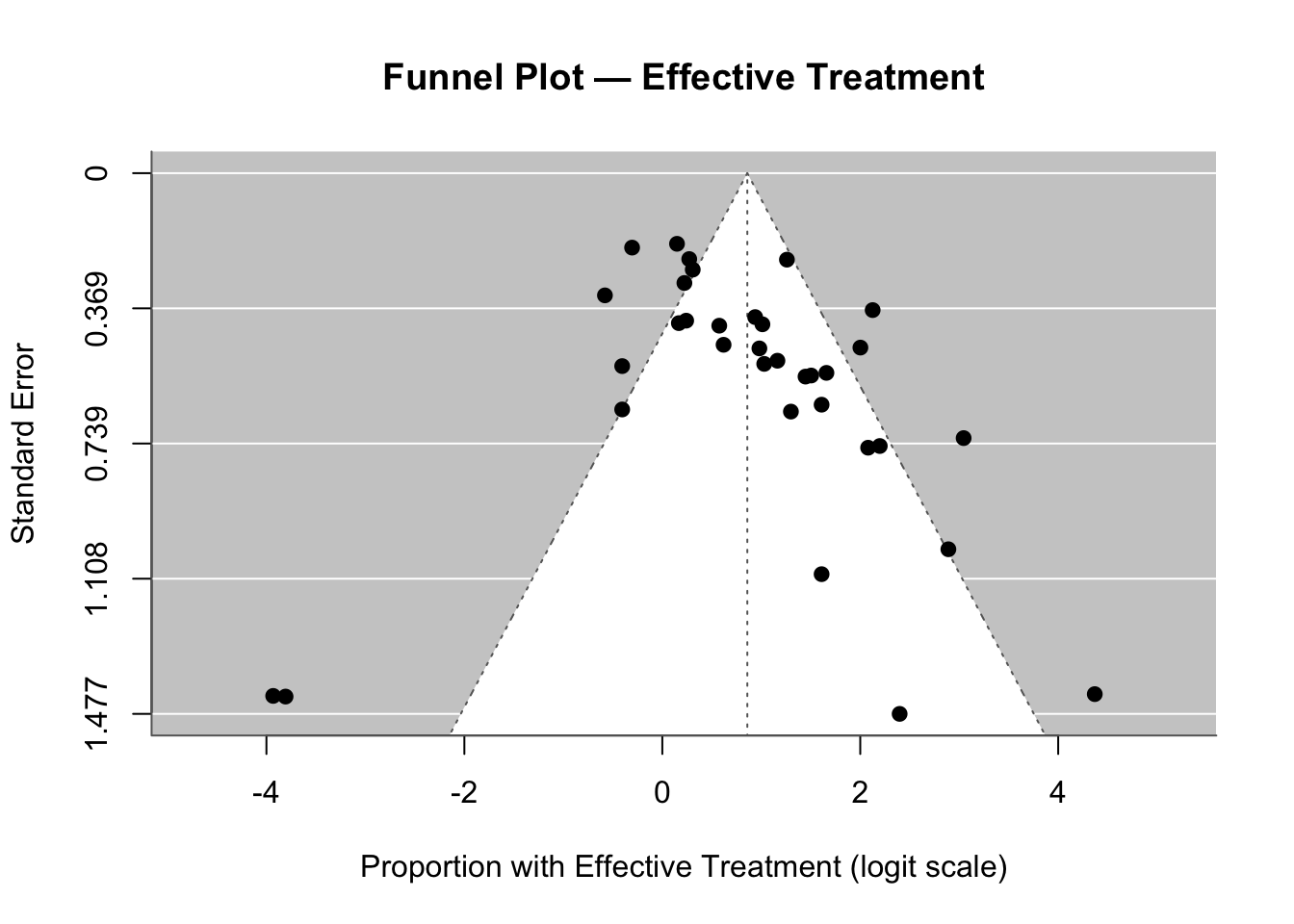


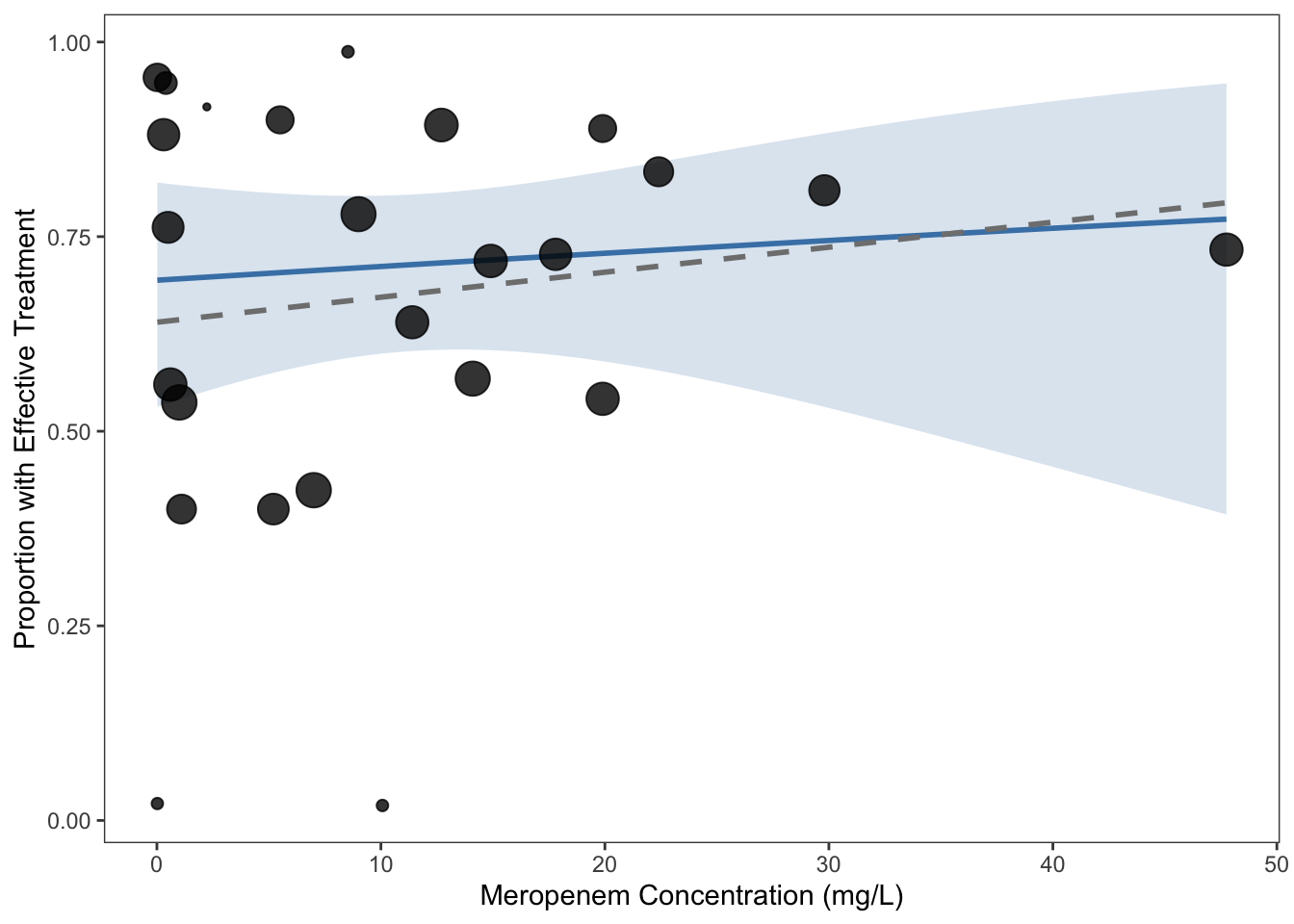
Figure S5 - meta-regression of clinical cure meta-analysis moderated by meropenem exposure. Bubbles represent individual cohorts. Size of bubbles indicates weighting of each cohort, adjusted for clustering within studies. Blue solid line and light blue ribbon: Linear meta-regression curve and 95% CI. Grey dotted line: unweighted ordinary least squares (OLS) fit, not accounting for study weights or random effects, shown for reference only.

Figure S6 – Forest plot of meta-regression of clinical cure meta-analysis moderated by stratified percentage of patients achieving 100% ƒT>MIC. 100% ƒT>MIC stratified into low achievement (< 48.2%), moderate achievement (48.2 – 81.7%), and high achievement (>81.7%).


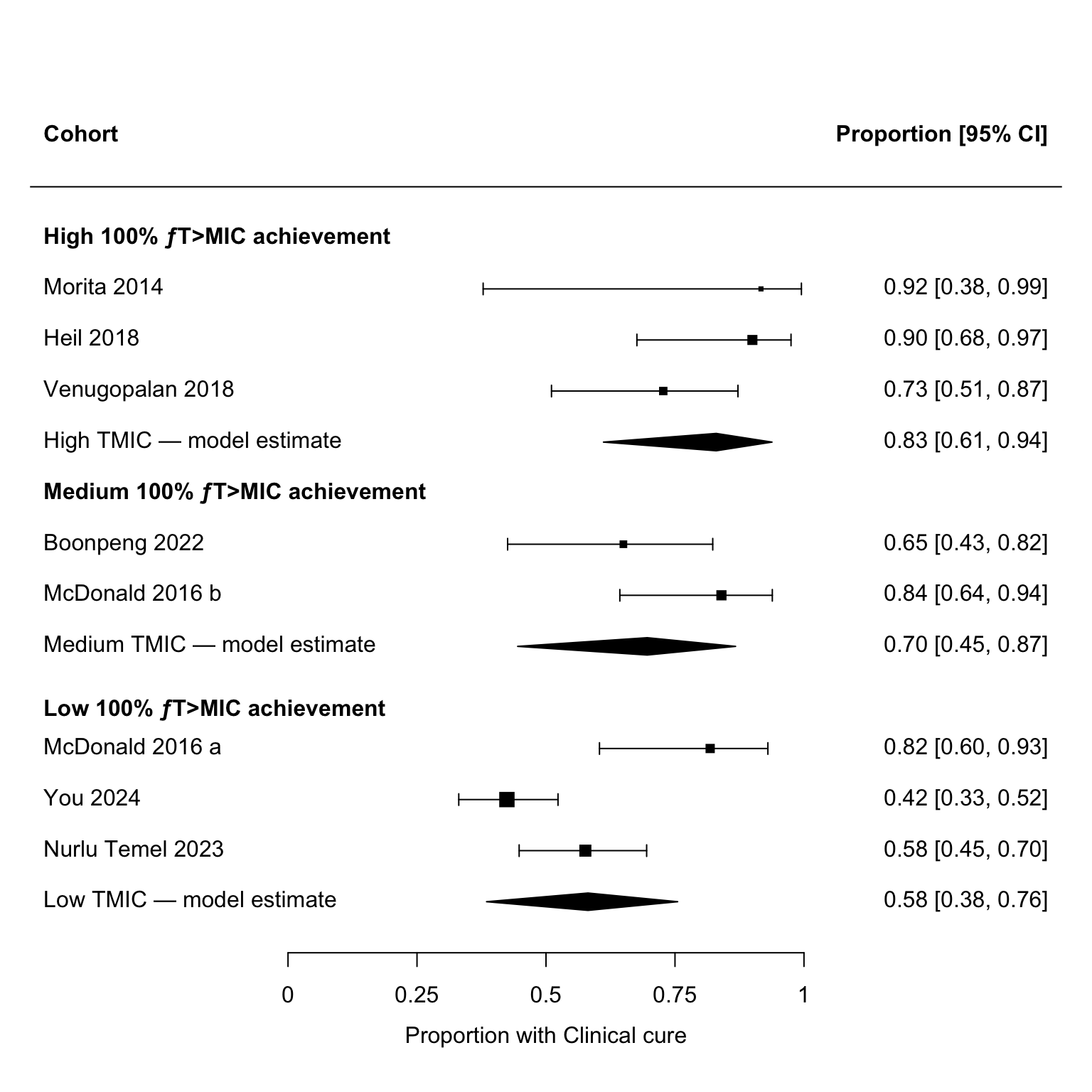


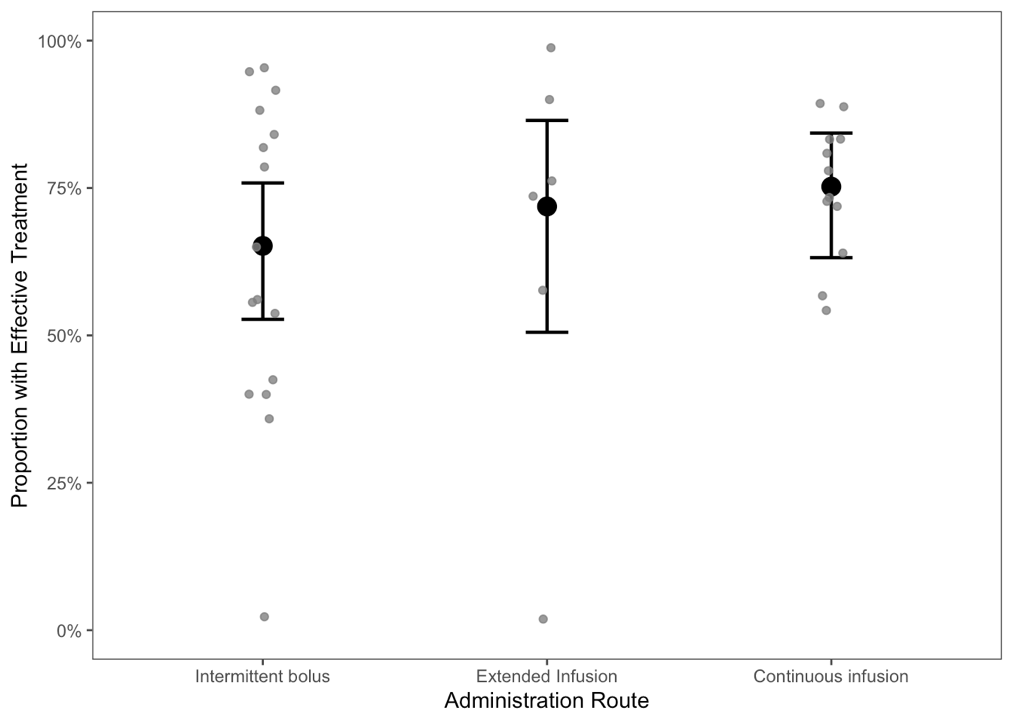


Figure S7 - Proportion of patients achieving clinical cure (effective treatment) by meropenem administration route. Points represent individual cohort estimates; large, filled circles indicate pooled estimates with 95% confidence intervals.

Table S2 – Baseline demographics of cohorts reporting a 30-day mortality outcome.

| **Cohort** | **Patient Group** | **n** | **Administration** | **Male n (%)** | **Age** | **Weight (kg)** | **BMI (kg/m²)** | **APACHE II** | **SOFA** | **CrCl (mL/min)** | **eGFR (mL/min)** | **Serum Creatinine (mg/dL)** | **MIC source** | **Surrogate MIC (mg/L)** | **Micro MIC (mg/L)** | **Conc (mg/L)** | **Mortality n (%)** |
| --- | --- | --- | --- | --- | --- | --- | --- | --- | --- | --- | --- | --- | --- | --- | --- | --- | --- |
| Chiriac 2023 | Critically ill | 91 | Continuous infusion | 60 (65.9) | 73 (14.6) | 80 (21.1) | 27.9 (6.1) | 22 (9.2) | 6 (5) | 45.3 (53.7) | NA | 1.60 (1.60) | Surrogate | 2 | NA | 14.1 | 30 (33.0) |
| Del Bono 2016 | Critically ill | 19 | Extended Infusion | 12 (63) | 62 (13) | NA | 26.0 (10.0) | 11.0 (3.7) | NA | NA | NA | 0.90 (0.52) | Microbiological | NA | 512 | NA | 3 (15.8) |
| Drager 2023 | Critically ill | 186 | Continuous infusion | 136 (73.1) | 66 (13.3) | 80.0 (16.3) | 26 (5.19) | NA | NA | NA | 63 (47.4) | 1.16 (1.01) | Surrogate | 2 | NA | 21 | 74 (39.8) |
| Gatti 2023 | Critically ill | 24 | Continuous infusion | 15 (62.5) | 68 (13) | 67.5 (14.8) | 24 (4.15) | NA | 14.0 (4.6) | NA | NA | NA | Microbiological | NA | 0.12 | 19.9 | 12 (50) |
| Gatti 2024 | Critically ill | 32 | Continuous infusion | 24 (75) | 71.5 (11.1) | 80.0 (14.8) | 27.6 (6.15) | 19.4 (6.3) | 9.0 (5.4) | NA | NA | NA | Microbiological | NA | 0.12 | 14.9 | 7 (21.9) |
| Guilhaumou 2023 | Critically ill | 30 | Continuous infusion | 18 (60) | 61 (16.1) | 74.0 (13.3) | 25.4 (4.3) | NA | 5.0 (1.5) | NA | 24.8 (33.8) | 1.13 (0.60) | Surrogate | 2 | NA | 18 | 8 (26.7) |
| Hatti 2018 | Elderly Patients | 12 | Extended Infusion | 7 (58) | 75 (7.5) | NA | 22.9 (4.7) | NA | NA | NA | 60.5 (38.1) | NA | Surrogate | 2 | NA | 2.38 | 1 (8.3) |
| Heil 2018 | Critically ill | 20 | Extended Infusion | 10 (50) | 55.5 (16.7) | 89.7 (38.2) | 33.4 (14.0) | 15.0 (6.0) | 4 (2.0) | NA | NA | 0.74 (1.00) | Microbiological | NA | 0.25 | 5.5 | 4 (20) |
| Maimongkol 2022 a | Critical care paediatrics | 54 | Extended Infusion | 25 (46) | 1.0 (2.0) | 8.8 (7.6) | NA | NA | NA | 72.9 (36.2) | 127.9 (46.1) | NA | Microbiological | 2 | NA | 2.3 | 2 (3.7) |
| Maimongkol 2022 b | Critical care paediatrics | 18 | Intermittent bolus | 7 (39) | 1.0 (2.3) | 5.2 (7.9) | NA | NA | NA | NA | 119.0 (52.3) | NA | Surrogate | 2 | NA | 0.8 | 3 (16.7) |
| Paice 2024 | Critical care paediatrics | 29 | Intermittent bolus | NA | 4 (8.9) | 16.3 (20.2) | NA | 11.0 (3.7) | NA | NA | 133.0 (48.1) | NA | Microbiological | 1 | NA | NA | 4 (13.8) |
| Sanz Codina 2022 | Critically ill | 43 | Continuous infusion | 26 (60.5) | 59 (13.8) | NA | 30.7 (14.9) | NA | 9 (1.2) | NA | 84.4 (39.3) | NA | Microbiological | NA | NA | 22.4 | 20 (46.5) |
| Tseng 2025 | Patients with low body weight | 20 | Intermittent bolus | 11 (55) | 65 (14.8) | 41.8 (7.0) | NA | NA | NA | 50.5 (16.9) | 115.4 (36.8) | NA | Surrogate | 2 | NA | 8.76 | 0 (0) |
| Venugopalan 2018 | General hospital patients | 22 | Continuous infusion | 16 (73) | 44 (26.7) | 69.0 (12.6) | 24.0 (7.4) | NA | NA | NA | NA | NA | Surrogate | 2 | 2 | 17.8 | 1 (4.5) |
| Wang 2016 a | Critically ill | 104 | Continuous infusion | 63 (61) | 60.4 (14.9) | NA | NA | 19.5 (6.4) | 5.9 (2.7) | NA | NA | 0.70 (0.22) | Combination | 2 | NA | 9 | 14 (13.5) |
| Wang 2016 b | Critically ill | 108 | Intermittent bolus | 70 (65) | 57.8 (15.3) | NA | NA | 18.8 (6.7) | 6.2 (2.9) | NA | NA | NA | Surrogate | 2 | NA | 1 | 38 (35.2) |

Abbreviations: BMI = body mass index; APACHE II = Acute Physiology and Chronic Health Evaluation II score; SOFA = Sequential Organ Failure Assessment score; CrCl = creatinine clearance; eGFR = estimated glomerular filtration rate; MIC = minimum inhibitory concentration; Conc = measured meropenem concentration (mg/L); NA = not available or not reported; n = number of patients.

Figure S8 - a) Leave-one-out sensitivity analysis of 30-day mortality meta-analysis and impact on pooled proportion. Red dotted line – pooled proportion when all studies included. Blue points and bars - Point estimates and 95% CI. b) Funnel plot with 95% pseudo-confidence intervals. Dots represent individual cohorts.


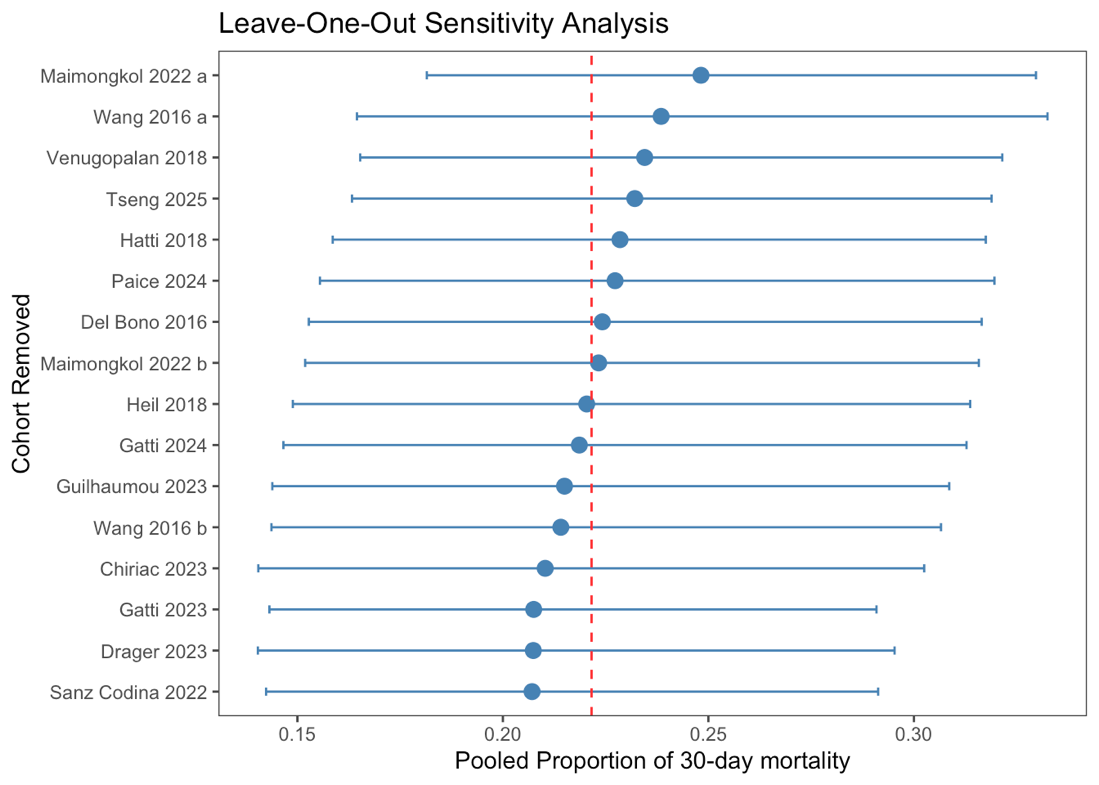

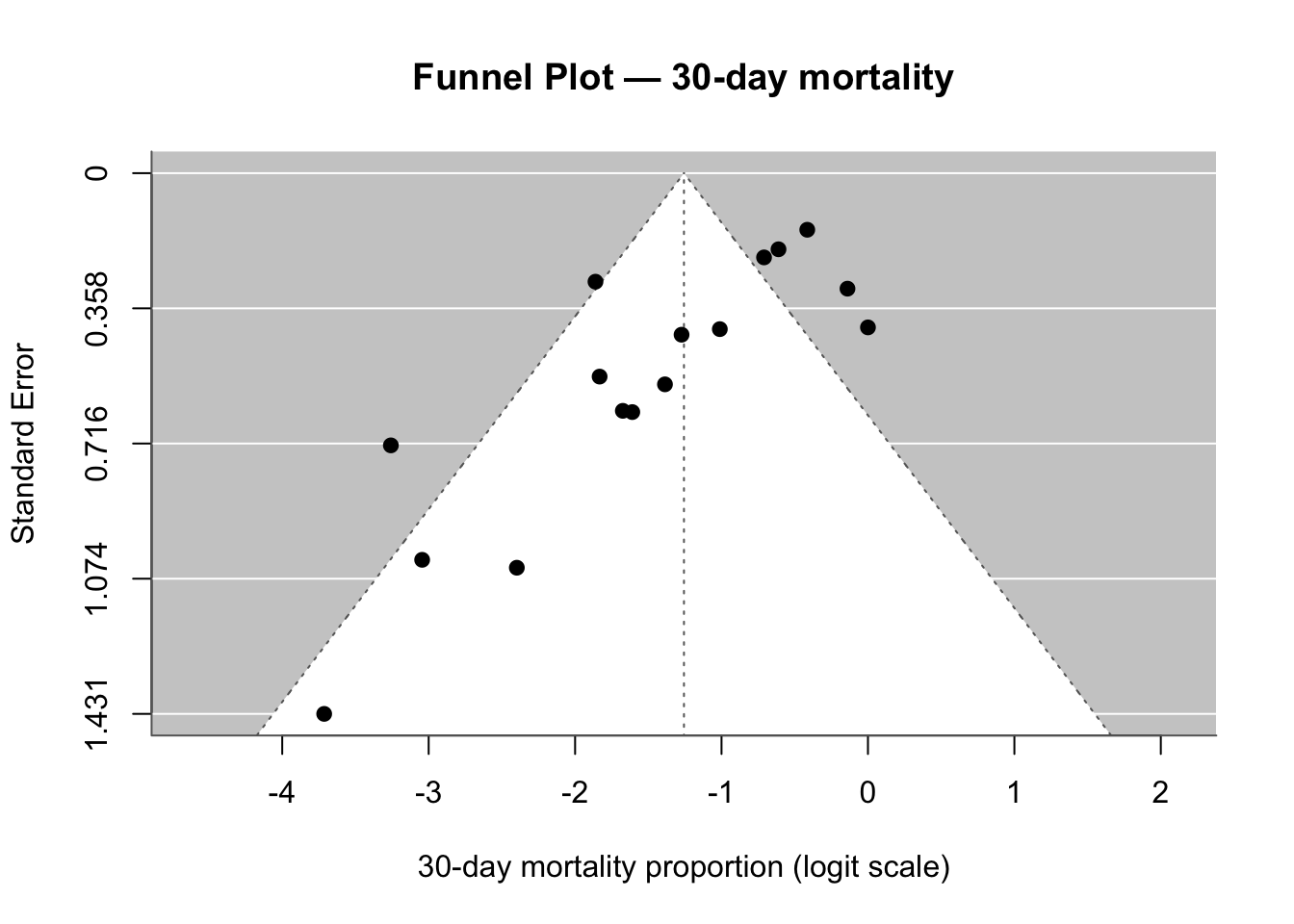


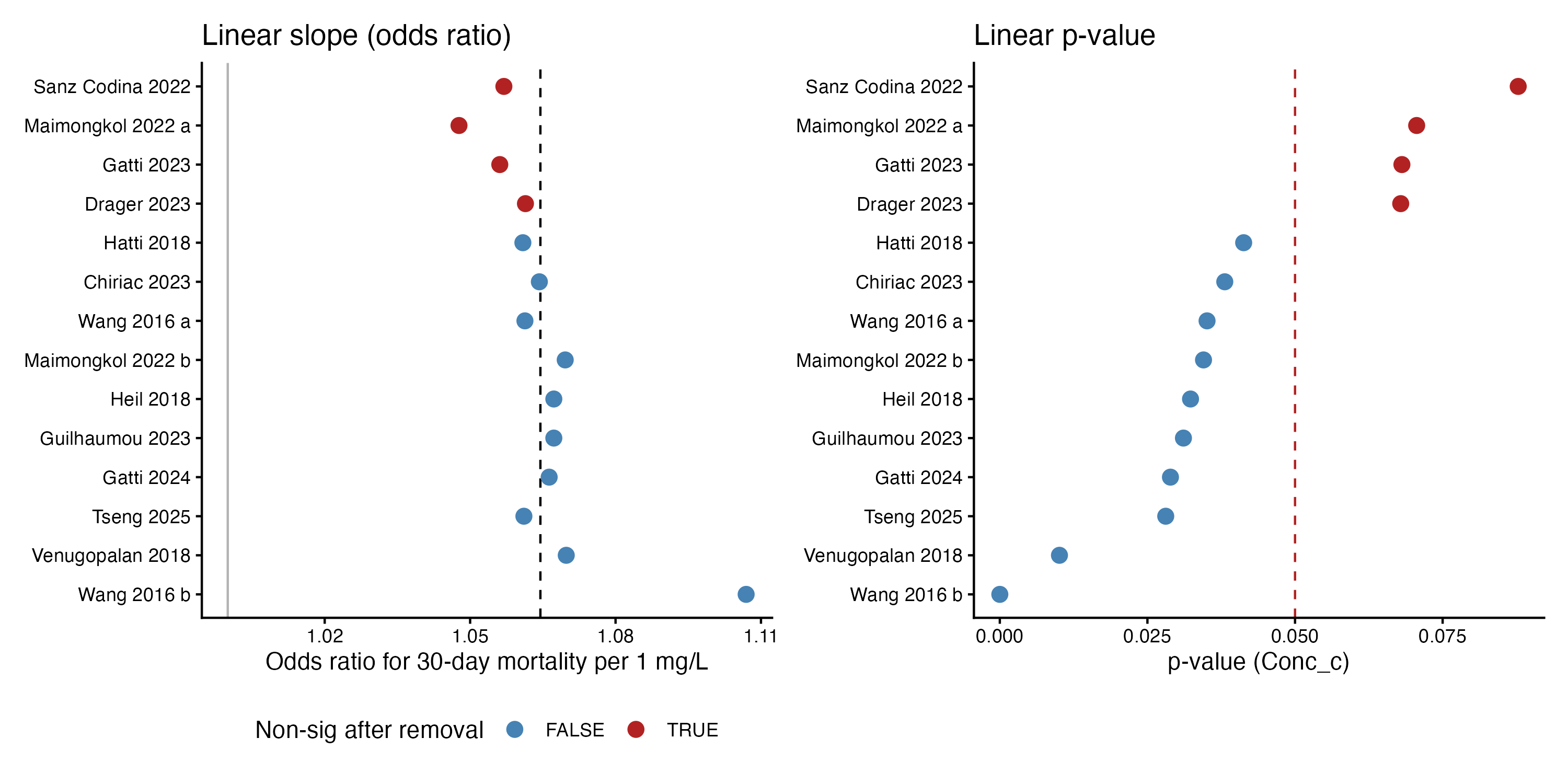


Figure S10 - a) Leave-one-out sensitivity analysis of the quadratic concentration-mortality slope. b) Leave-one-out sensitivity analysis of the quadratic concentration-mortality slope p-value.

Figure S9 - a) Leave-one-out sensitivity analysis of the linear concentration-mortality slope odds ratio. b) Leave-one-out sensitivity analysis of the linear concentration-mortality slope p-value.


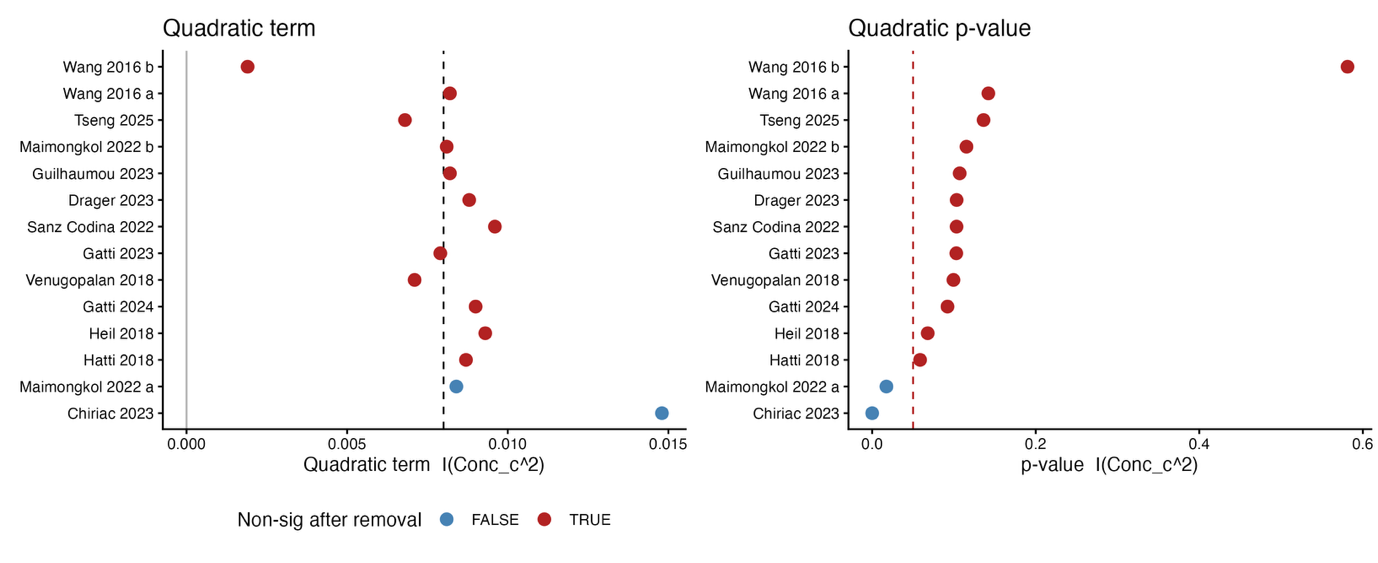


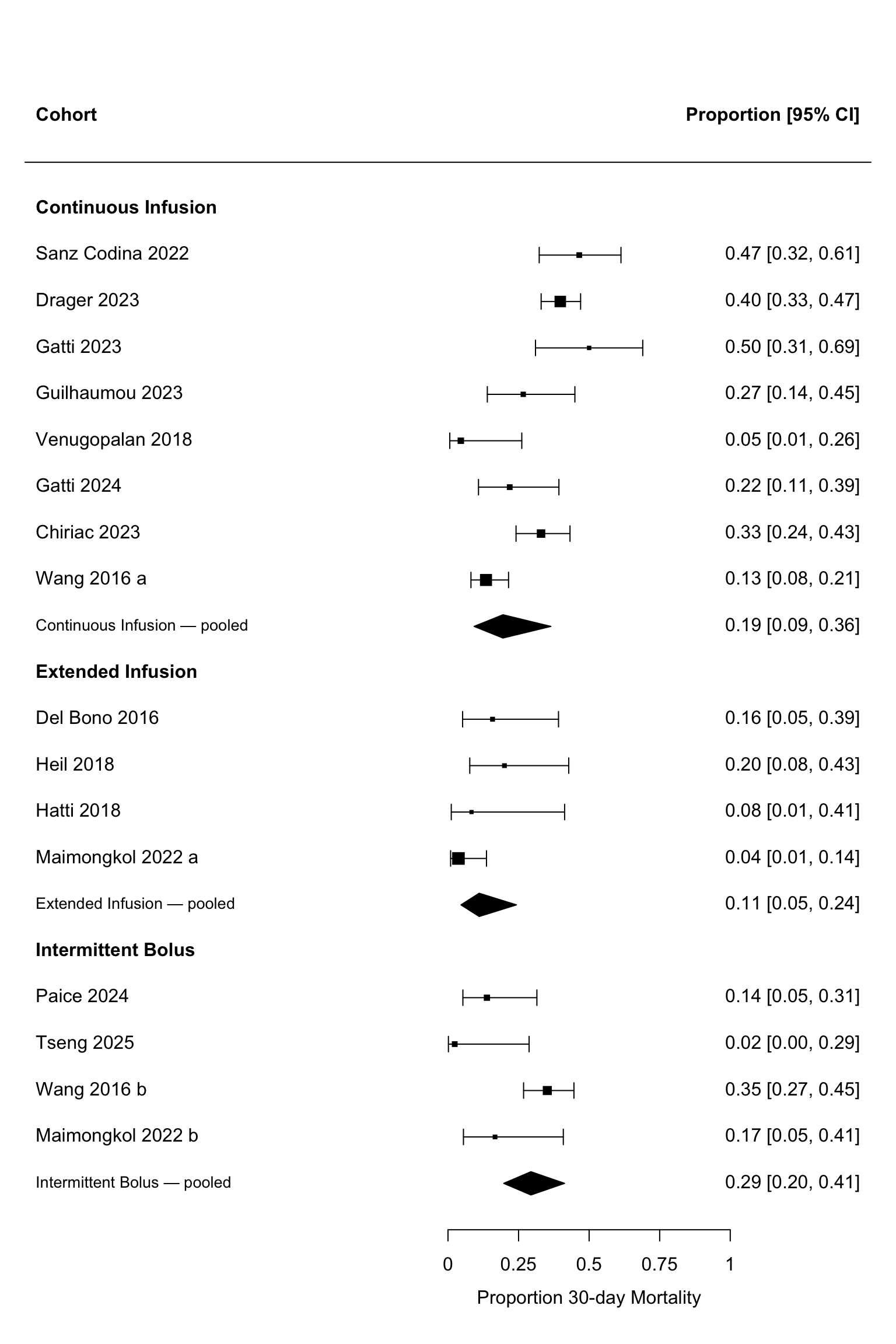
Figure S11 – Forest plot of cohorts and proportion of 30-day mortality in patients, stratified by meropenem administration route.


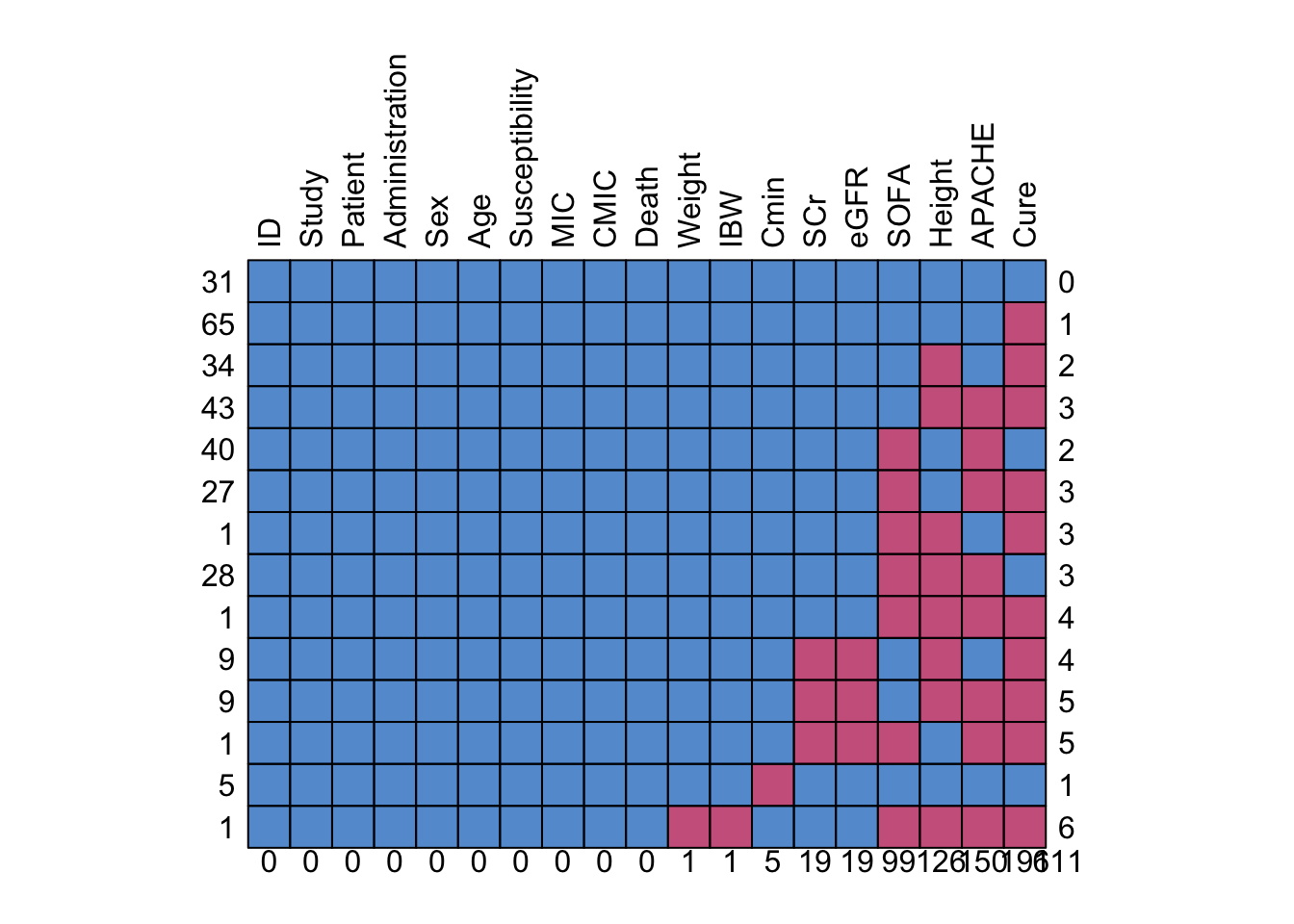
Figure S12 - Patient level analysis: Missing data pattern prior to multiple imputation. Blue cells indicate observed data; pink cells indicate missing values. Numbers on the right indicate the count of missing variables per row pattern; numbers at the bottom indicate the total number of missing observations per variable.


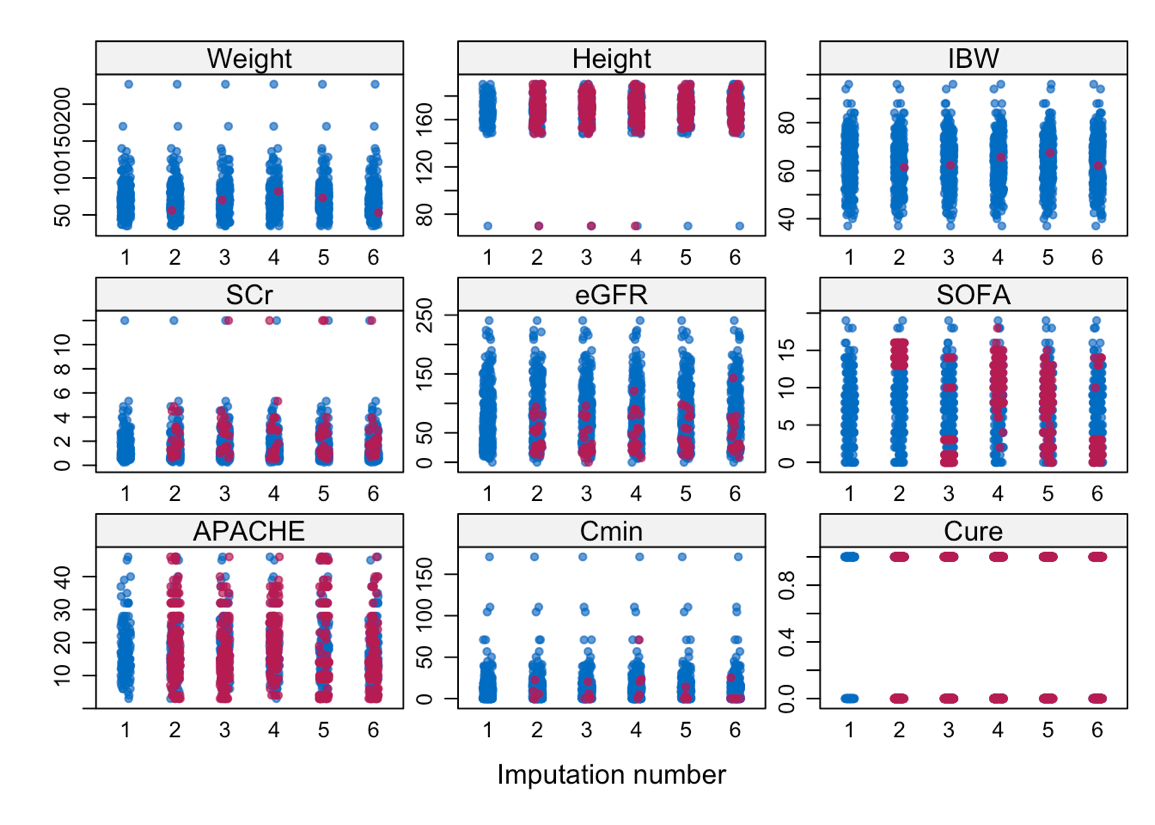


Figure S13 - Patient level analysis: Stripplot of observed and imputed values across five imputed datasets. Blue points represent observed values; pink points represent imputed values. Imputation number 1–5 reflects each of the five imputed datasets; column 0 represents the original observed data. Distributional similarity between observed and imputed values supports the plausibility of the imputation.

| **Variable** | **OR** | **95% CI** | **p-value** | **Degrees of Freedom** |
| --- | --- | --- | --- | --- |
| **Cmin or Css (mg/L)** | **1.04** | **1.02–1.06** | **<0.001 *** | **290.5** |
| C/MIC Ratio | 1.02 | 1–1.03 | 0.051 | 291.0 |
| **Measured vs Surrogate MIC** | **3.60** | **1.74–7.45** | **<0.001 *** | **291.0** |
| Extended Infusion | 1.12 | 0.38–3.28 | 0.834 | 290.0 |
| **Continuous Infusion** | **1.97** | **1.11–3.53** | **0.022 *** | **290.0** |
| **Serum Creatinine (mg/dL)** | **1.26** | **1–1.58** | **0.048 *** | **99.1** |
| SOFA Score | 1.18 | 0.93–1.49 | 0.138 | 4.9 |
| **Age (years)** | **1.03** | **1.01–1.05** | **<0.001 *** | **291.0** |
| *OR = odds ratio. CI = confidence interval. Degrees of freedom reflect Rubin's rules pooling across m = 5 imputed datasets. Reference categories: Intermittent Bolus (Extended Infusion), Intermittent Bolus (Continuous Infusion), Male (Sex), Measured MIC (Measured vs Surrogate MIC)* p < 0.05.* | | | | |

Table S3 – Patient level analysis: Unadjusted associations with 30-day mortality (multiply imputed data, m=5).

Figure S Patient level analysis: a) ROC curve of multivariate logistic regression. b) Predicted Probability plot of mortality.


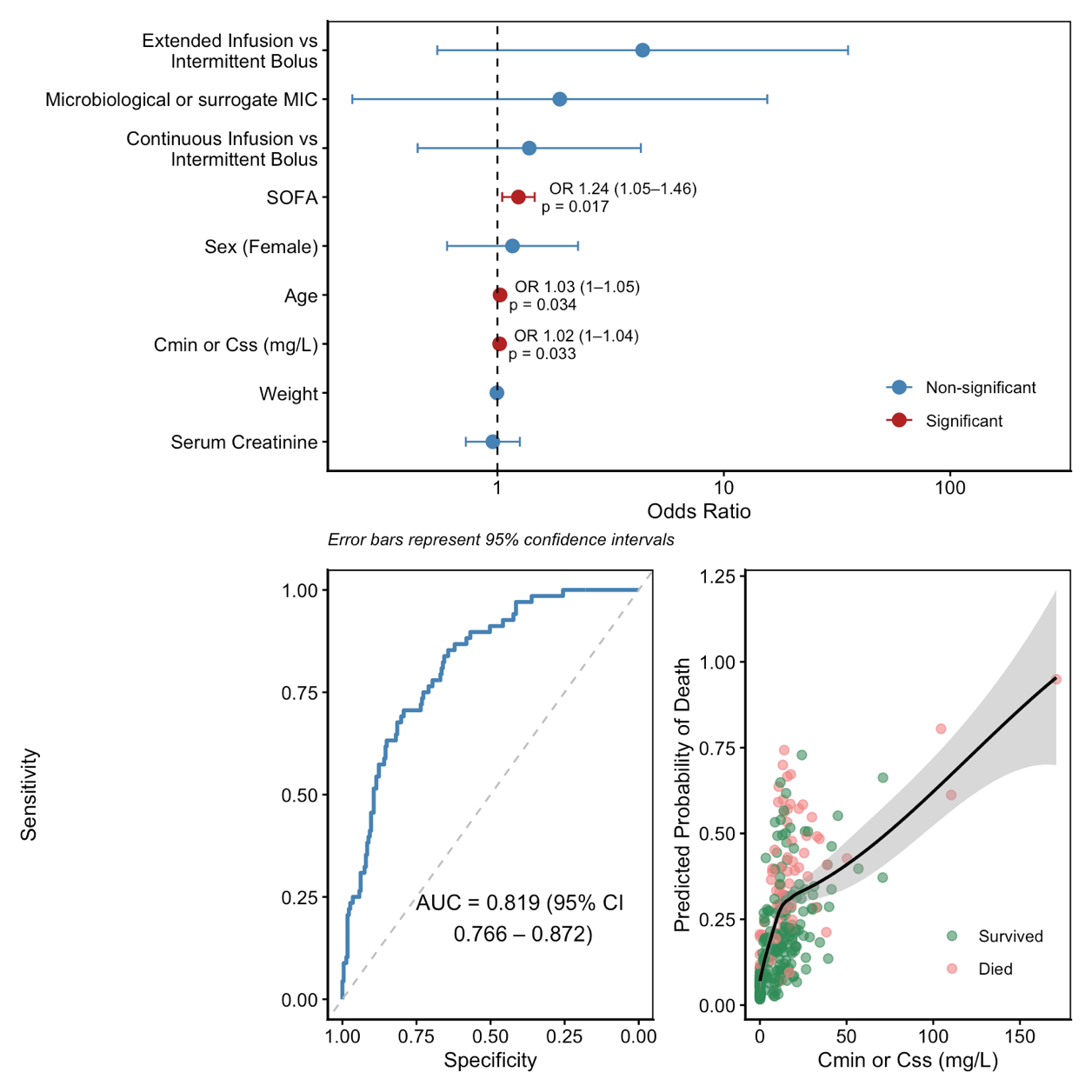

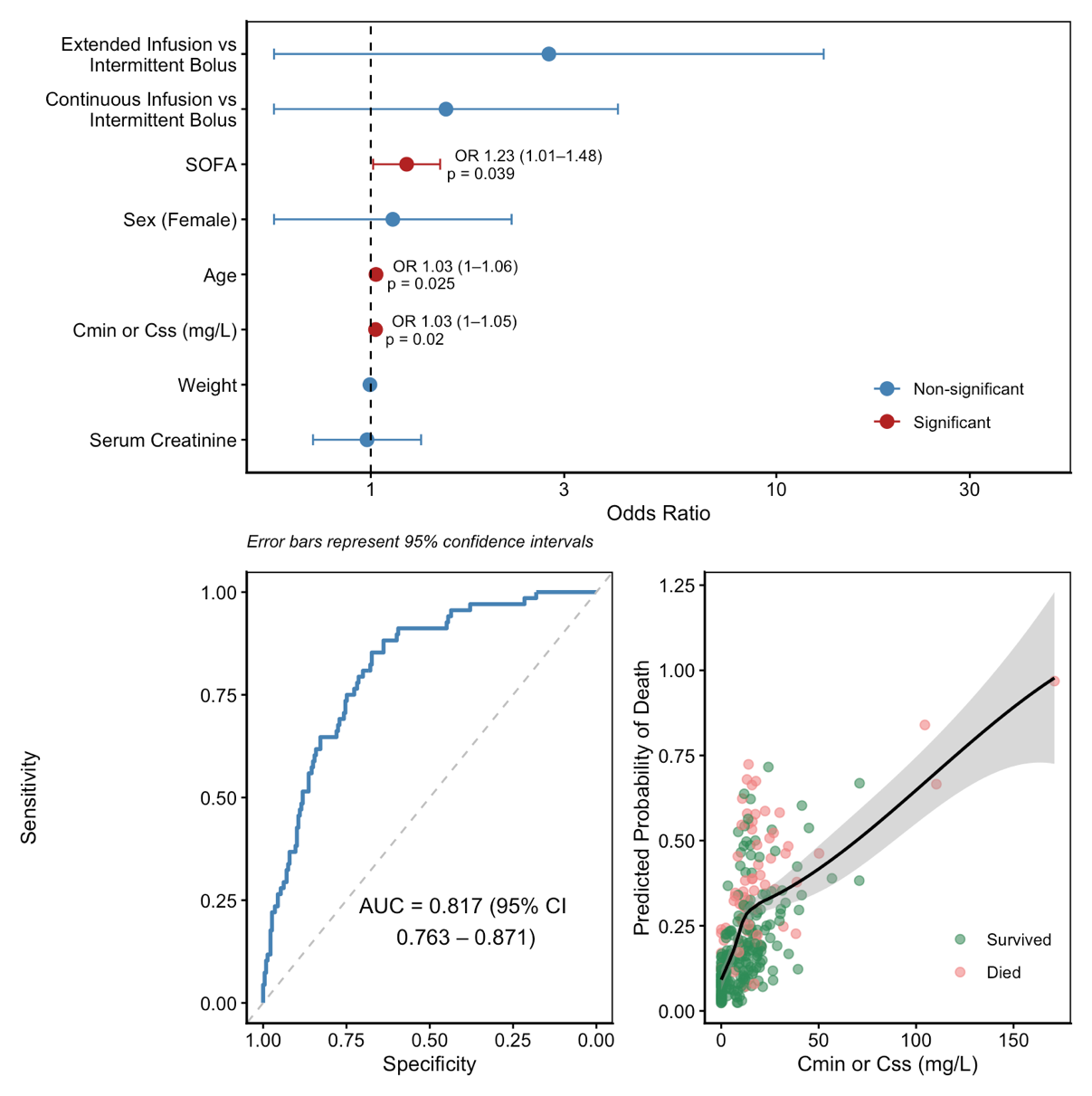


a)

b)

Figure S14 – Patient level analysis: a) plot of unadjusted logistic regression and 95% CI of the association between meropenem exposure (Cmin or Css) and probability of mortality. b) and c) box plot of survivors and non-survivors plotted by meropenem concentration. d) and e) density plot of meropenem exposure by probability of mortality.


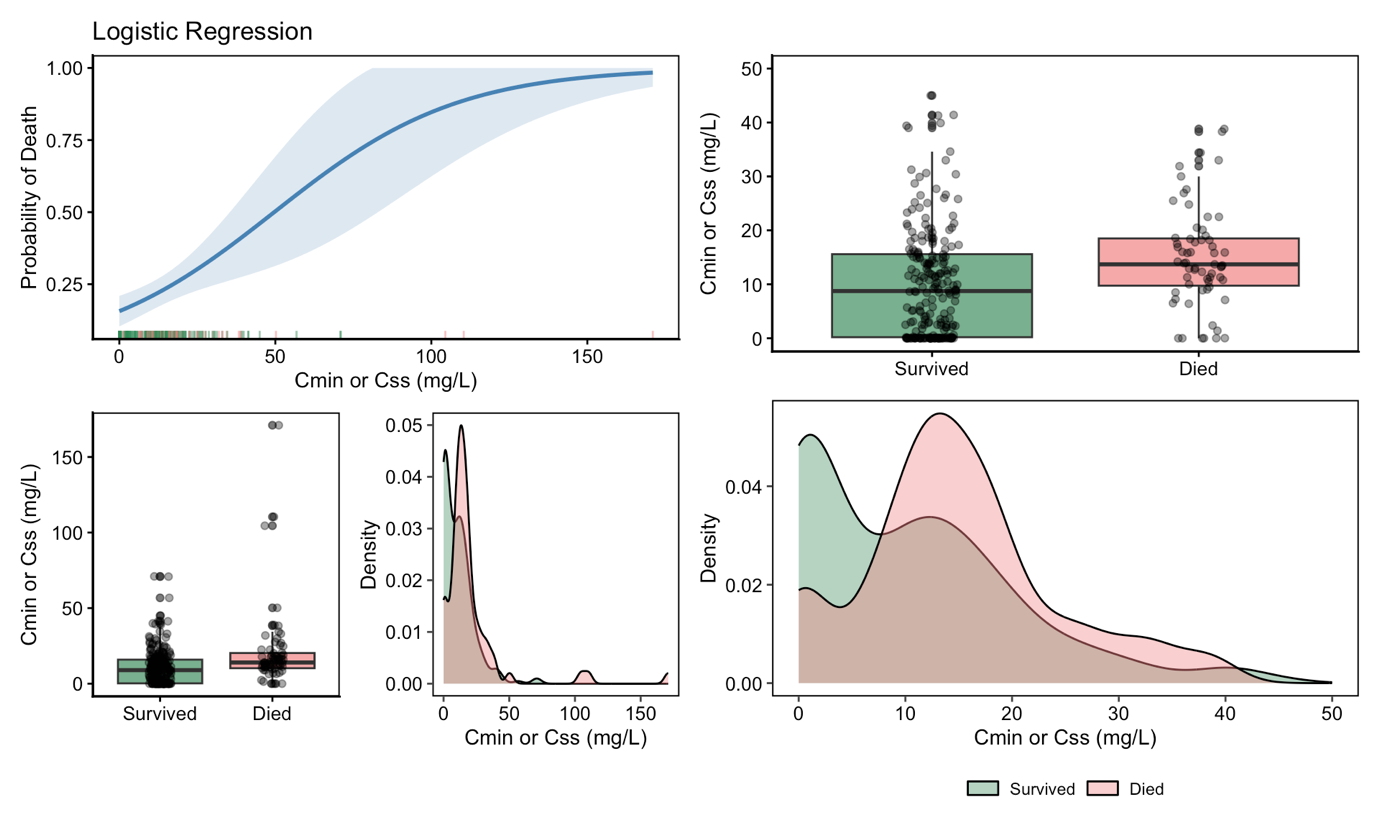


a)

b)

c)

d)

e)


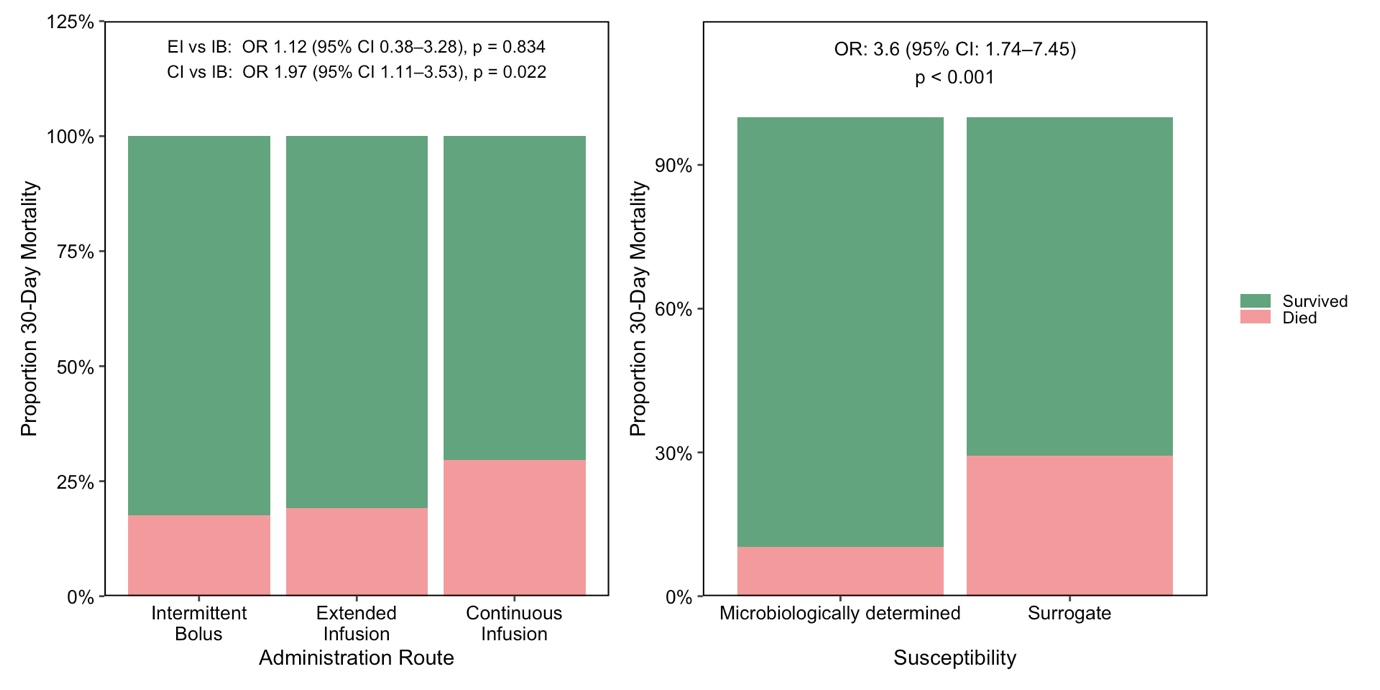


Figure S15 - Patient level analysis: Unadjusted logistic regression of a) administration route and b) whether a microbiologically determined or a surrogate MIC was used for treatment.

Figure S16 a) ROC curve of multivariate logistic regression. b) Predicted Probability plot of mortality.


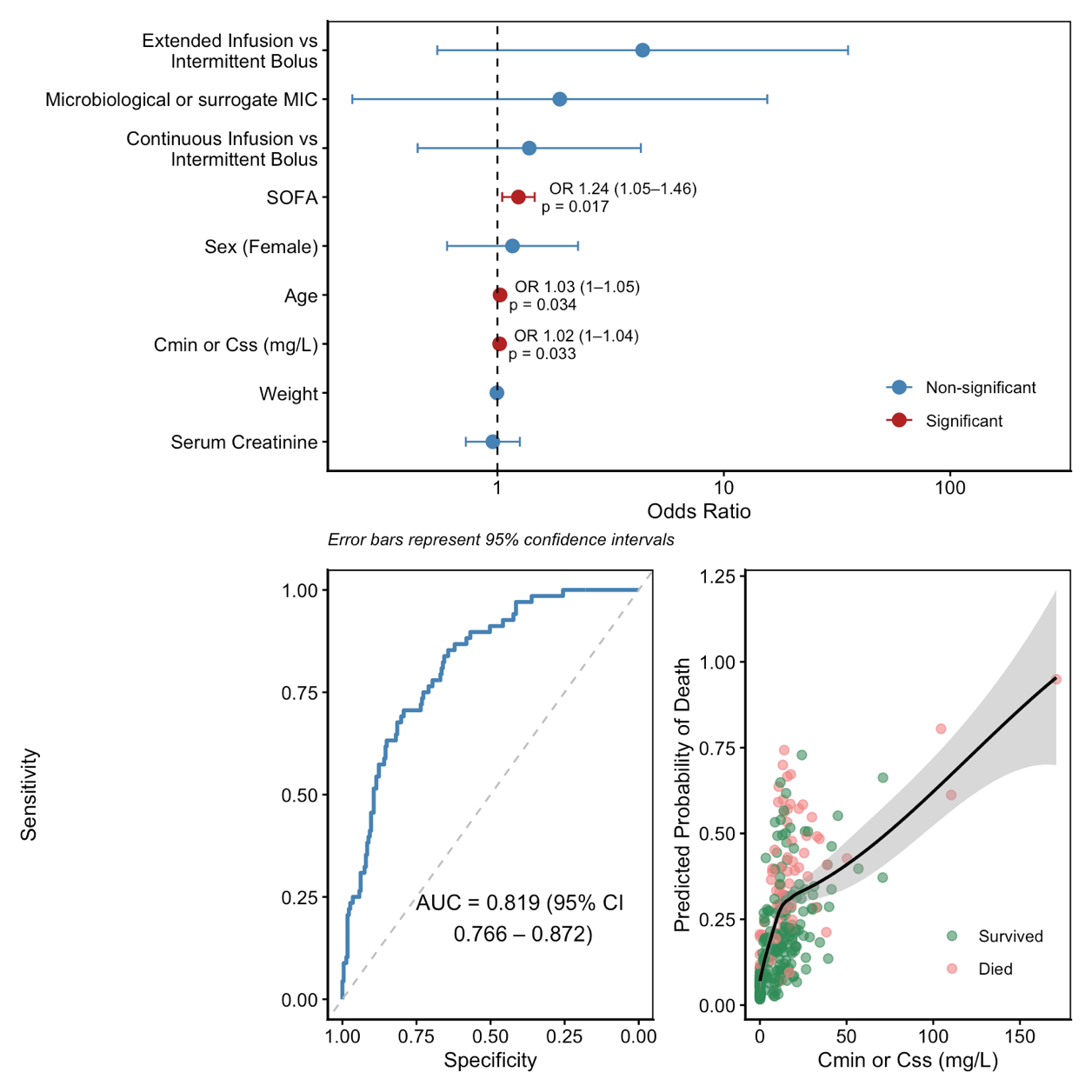

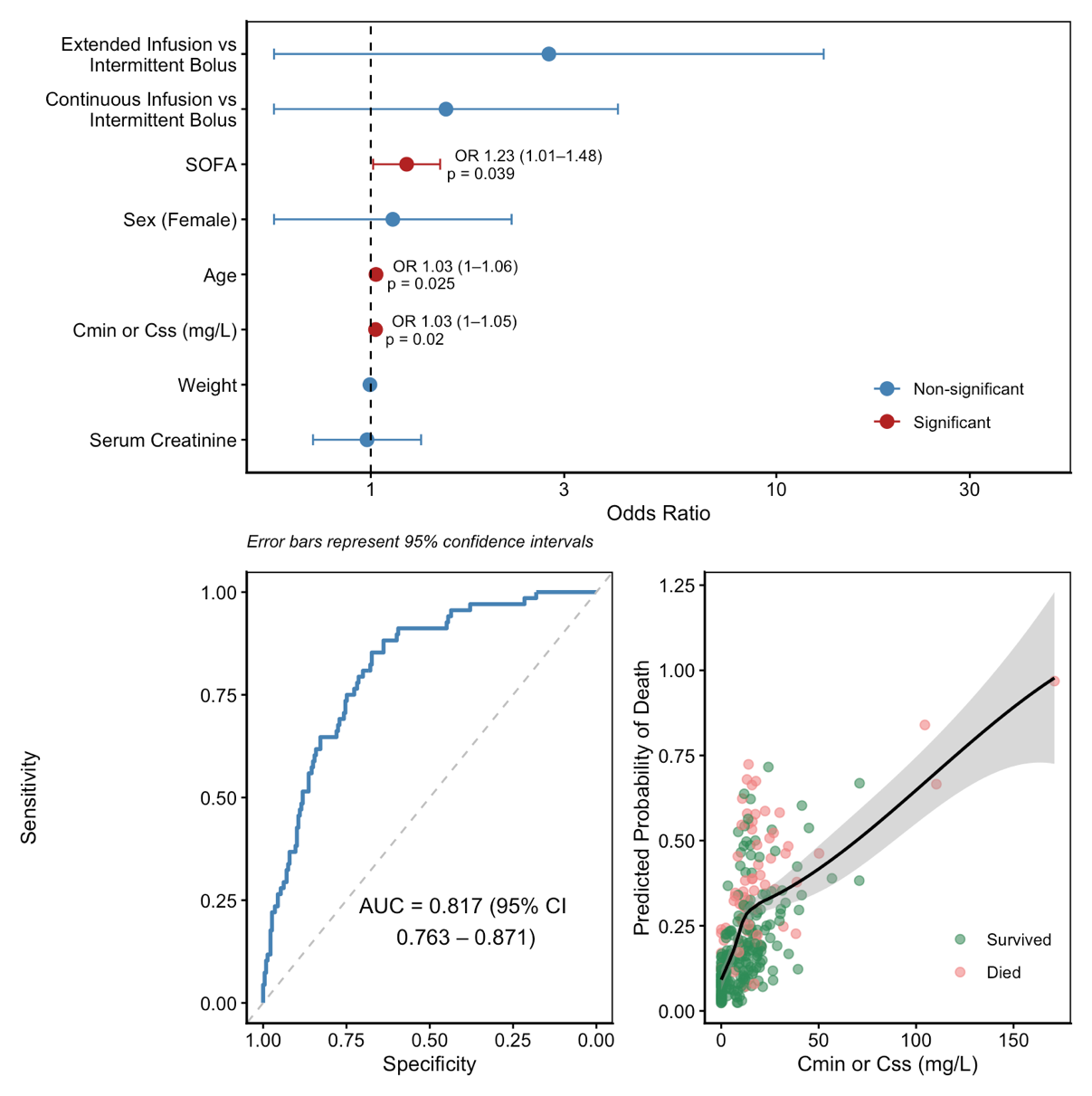


a)

b)

### Supplementary material 3 – Search strategy

### Methods

Searches originally conducted in April 2024 and rerun in full on 25^th^ March 2025 in the following academic databases:

- Cochrane Database of Systematic Reviews (Wiley): 3 of 12, March 2025
- Cochrane Central Register of Controlled Trials (Wiley): Issue 3 of 12, March 2025
- Dimensions <https://app.dimensions.ai/discover/publication>
- Embase Classic+Embase (Ovid) 1947 to 2025 March 24
- International Pharmaceutical Abstracts (Ovid) 1970 to March 2025
- MEDLINE (Ovid)) ALL 1946 to March 24, 2025
- Scopus
- Core Collection (Web of Science) SCI-EXPANDED 1900+, SSCI 1900+, A&HCI 1975+, CPCI-S 1900+, CPCI-SSH 1900+, and ESCI 2015+ searched simultaneously.

For studies examining therapeutic drug monitoring of meropenem.

Searches were developed for the concepts: meropenem and drug monitoring. Subject headings and free text words were identified for use in the search concepts by text analysis tools the Information Specialist and project team members. Further terms were identified and tested from known relevant papers.

Limits for language and publication date were not used but where the databases permitted conference abstracts and animal only studies were removed. The search was peer-reviewed by a second Information Specialist using the PRESS checklist.

Search results were managed in an EndNote library where duplicates were removed automatically and manually using University of Leeds AUHE guidance.

### Results

The database searches identified 15 738 records. Once duplicates were removed there were 6870 records to screen.

Breakdown of database result for PRISMA diagram:

- Cochrane (n= 354)
- Dimensions (n=358)
- EMBASE (n= 4522)
- IPA (n = 515)
- MEDLINE (n= 2082)
- Scopus (n= 5112)
- Web of Science (n= 2795)

## Cochrane library

Date Run: 25 March 2025

Simultaneous search of the following:

- Cochrane Database of Systematic Reviews: Issue 3 of 12, March 2025 (n=2)
- Cochrane Central Register of Controlled Trials: Issue 3 of 12, March 2025 (n=352)

ID Search Hits

#1 MeSH descriptor: [Meropenem] explode all trees 362

#2 meropenem*:ti,ab,kw 862

#3 (mepem or meronem or meropen or merrem or ronem):ti,ab,kw 42

#4 #1 OR #2 OR #3 867

#5 MeSH descriptor: [Drug Monitoring] explode all trees 2286

#6 ((drug or antibiotic) NEAR/2 (level or measur* or concentration*)):ti,ab,kw 22428

#7 ((blood or serum or plasma) near/2 (level or measur* or concentration*)):ti,ab,kw 159320

#8 (pharmacokinetic* or pharmacodynamic*):ti,ab,kw 101482

#9 MeSH descriptor: [Pharmacokinetics] explode all trees 20373

#10 (pkpd or "pk/pd" or tdm):ti,ab,kw 3018

#11 ((drug or antibiotic*) near/2 (Dosing or dosage* or regime* or schedul* or monitor*)):ti,ab,kw 42686

#12 ((drug or antibiotic*) near/2 (adjust* or personali* or optimi*)):ti,ab,kw 1308

#13 (dos* near/2 (adjust* or alter* or escalat* or optimi* or personali*)):ti,ab,kw 24114

#14 #5 OR #6 OR #7 OR #8 OR #9 OR #10 OR #11 OR #12 OR #13 283621

#15 #4 AND #14 316

#16 ((meropenem* or mepem or meronem or meropen or merrem or ronem) near/2 (level or measur* or concentration*)):ti,ab,kw 69

#17 ((meropenem* or mepem or meronem or meropen or merrem or ronem) near/2 (Dosing or dosage* or regime* or schedul* or monitor*)):ti,ab,kw 62

#18 ((meropenem* or mepem or meronem or meropen or merrem or ronem) near/2 (adjust* or personali* or optimi*)):ti,ab,kw 7

#19 #15 OR #16 OR #17 OR #18 354

## Dimensions https://app.dimensions.ai/

Search date: 25 March 2025

- Searched using Advanced interface
- search in: Title and abstract

(meropenem* OR mepem OR meronem OR meropen OR merrem OR ronem) AND "drug monitoring"

- PUBLICATIONS 358
- DATASETS 3
- GRANTS 7
- PATENTS 2
- CLINICAL TRIALS 15
- POLICY DOCUMENTS 0

## Embase Classic+Embase <1947 to 2025 March 24>

Search date: 25 March 2025

1 meropenem/ or meropenem plus vaborbactam/ 57561

2 meropenem*.mp. 58979

3 (mepem or meronem or meropen or merrem or ronem).mp. 499

4 or/1-3 [meropenem] 59023

5 drug monitoring/ 62886

6 ((drug or antibiotic) adj2 (level? or measur* or concentration*)).tw,kf. 75830

7 ((blood or serum or plasma) adj2 (level? or measur* or concentration*)).tw,kf. 1303456

8 (pharmacokinetic* or pharmacodynamic*).tw,kf. 324913

9 pharmacokinetics/ 273560

10 pharmacodynamics/ 42092

11 (pkpd or "pk/pd" or tdm).tw,kf. 20878

12 ((drug or antibiotic*) adj2 (Dosing or dosage* or regime* or schedul* or monitor*)).tw,kf. 75400

13 ((drug or antibiotic*) adj2 (adjust* or personali* or optimi*)).tw,kf. 13090

14 (dos* adj2 (adjust* or alter* or escalat* or optimi* or personali*)).tw,kf. 112519

15 or/5-14 [drug monitoring terms] 1925296

16 4 and 15 6000

17 ((meropenem* or mepem or meronem or meropen or merrem or ronem) adj2 (level? or measur* or concentration*)).tw,kf. 605

18 ((meropenem* or mepem or meronem or meropen or merrem or ronem) adj2 (Dosing or dosage* or regime* or schedul* or monitor*)).tw,kf. 394

19 ((meropenem* or mepem or meronem or meropen or merrem or ronem) adj2 (adjust* or personali* or optimi*)).tw,kf. 69

20 or/16-19 [meropenem drug levels] 6277

21 exp animals/ not exp human/ 6647454

22 exp nonhuman/ not exp human/ 5654064

23 exp experimental animal/ 918144

24 exp veterinary medicine/ 111557

25 animal experiment/ 3298458

26 or/21-25 9375412

27 20 not 26 [ tdm & meropenems] 5344

28 limit 27 to conference abstract 822

29 27 not 28 4522

## International Pharmaceutical Abstracts <1970 to March 2025>

Search date: 25 March 2025

1 meropenem*.mp. 987

2 (mepem or meronem or meropen or merrem or ronem).mp. 27

3 or/1-2 [meropenems] 988

4 ((drug or antibiotic) adj2 (level? or measur* or concentration*)).tw,hw. 10369

5 ((blood or serum or plasma) adj2 (level? or measur* or concentration*)).tw,hw. 77956

6 (pharmacokinetic* or pharmacodynamic*).tw,hw. 84397

7 (pkpd or "pk/pd" or tdm).tw,hw. 1771

8 ((drug or antibiotic*) adj2 (Dosing or dosage* or regime* or schedul* or monitor*)).tw,hw. 11108

9 or/4-8 [drug monitoring] 129108

10 3 and 9 450

11 ((meropenem* or mepem or meronem or meropen or merrem or ronem) adj2 (level? or measur* or concentration*)).tw,hw. 297

12 ((meropenem* or mepem or meronem or meropen or merrem or ronem) adj2 (Dosing or dosage* or regime* or schedul* or monitor*)).tw,hw. 204

13 ((meropenem* or mepem or meronem or meropen or merrem or ronem) adj2 (adjust* or personali* or optimi*)).tw,hw. 14

14 or/10-13 [meropenem TDM] 528

15 limit 14 to "abstracts of meeting presentations" 13

16 14 not 15 515

## Ovid MEDLINE(R) ALL <1946 to March 24, 2025>

Search date: 25 March 2025

1 Meropenem/ 4065

2 meropenem*.mp. 12008

3 (mepem or meronem or meropen or merrem or ronem).mp. 71

4 or/1-3 [meropenem] 12046

5 Drug Monitoring/ 24467

6 ((drug or antibiotic) adj2 (level? or measur* or concentration*)).tw,kf. 54211

7 ((blood or serum or plasma) adj2 (level? or measur* or concentration*)).tw,kf. 915745

8 (pharmacokinetic* or pharmacodynamic*).tw,kf. 227270

9 Pharmacokinetics/ 9349

10 (pkpd or "pk/pd" or tdm).tw,kf. 11154

11 ((drug or antibiotic*) adj2 (Dosing or dosage* or regime* or schedul* or monitor*)).tw,kf. 50143

12 ((drug or antibiotic*) adj2 (adjust* or personali* or optimi*)).tw,kf. 8816

13 (dos* adj2 (adjust* or alter* or escalat* or optimi* or personali*)).tw,kf. 58550

14 or/5-13 [drug monitoring terms] 1210931

15 4 and 14 1976

16 ((meropenem* or mepem or meronem or meropen or merrem or ronem) adj2 (level? or measur* or concentration*)).tw,kf. 447

17 ((meropenem* or mepem or meronem or meropen or merrem or ronem) adj2 (Dosing or dosage* or regime* or schedul* or monitor*)).tw,kf. 290

18 ((meropenem* or mepem or meronem or meropen or merrem or ronem) adj2 (adjust* or personali* or optimi*)).tw,kf. 51

19 or/15-18 [meropenem drug levels] 2186

20 exp Animals/ not exp Humans/ 5320597

21 19 not 20 2082

## Web of Science Search Strategy

Search date: 25 March 2025

Simultaneous search of the Web of Science Core Collection

# Entitlements:

- WOS.SCI: 1900 to 2025

- WOS.AHCI: 1975 to 2025

- WOS.ESCI: 2015 to 2025

- WOS.ISTP: 1990 to 2025

- WOS.SSCI: 1900 to 2025

- WOS.ISSHP: 1990 to 2025

*Data updated 2025-03-23*

1: meropenem* (Topic) 12507

2: mepem or meronem or meropen or merrem or ronem (Topic) 116

3: #2 OR #1 12611

4: (drug or antibiotic) NEAR/2 (level$ or measur* or concentration*) (Topic) 87247

5: (blood or serum or plasma) NEAR/2 (level$ or measur* or concentration*) (Topic) 1122344

6: pharmacokinetic* or pharmacodynamic (Topic) 320117

7: pkpd or "pk/pd" or tdm (Topic) 19899

8: (drug or antibiotic*) NEAR/2 (Dosing or dosage* or regime* or schedul* or monitor*) (Topic) 95774

9: (drug or antibiotic*) NEAR/2 (adjust* or personali* or optimi*) (Topic) 16632

10: dos* near/2 (adjust* or alter* or escalat* or optimi* or personali*) (Topic) 79953

11: #10 OR #9 OR #8 OR #7 OR #6 OR #5 OR #4 1564273

12: #11 AND #3 2753

13: (meropenem* or mepem or meronem or meropen or merrem or ronem) near/2 (level$ or measur* or concentration*) (Topic) 619

14: (meropenem* or mepem or meronem or meropen or merrem or ronem) near/2 (Dosing or dosage* or regime* or schedul* or monitor*) (Topic) 577

15: (meropenem* or mepem or meronem or meropen or merrem or ronem) near/2 (adjust* or personali* or optimi*) (Topic) 71

16: #15 OR #14 OR #13 OR #12 3172

17: rat or rats or mice or mouse or swine or porcine or murine or sheep or lamb$ or pig$ or piglet$ or rabbit$ or cat$ or dog$ or cattle or bovine or monkey$ or trout or marmoset$ (Topic) 6147090

18: #16 NOT #17 2869

19: #16 NOT #17 2869

20: #16 NOT #17 and Meeting Abstract (Exclude – Document Types) 2795

## Scopus

Search date: 25 March 2025

23 ( ( TITLE-ABS-KEY ( ( meropenem* OR mepem OR meronem OR meropen OR merrem OR ronem ) W/2 ( adjust* OR personali* OR optimi* ) ) ) OR ( TITLE-ABS-KEY ( ( meropenem* OR mepem OR meronem OR meropen OR merrem OR ronem ) W/2 ( dosing OR dosage* OR regime* OR schedul* OR monitor* ) ) ) OR ( TITLE-ABS-KEY ( ( meropenem* OR mepem OR meronem OR meropen OR merrem OR ronem ) W/2 ( level* OR measur* OR concentration* ) ) ) OR ( ( ( INDEXTERMS ( "drug monitoring" ) ) OR ( INDEXTERMS ( "pharmacodynamics" OR "pharmacokinetics" ) ) OR ( TITLE-ABS ( ( drug OR antibiotic ) W/2 ( level* OR measur* OR concentration* ) ) ) OR ( TITLE-ABS ( ( blood OR serum OR plasma ) W/2 ( level* OR measur* OR concentration* ) ) ) OR ( TITLE-ABS ( pharmacokinetic* OR pharmacodynamic* ) ) OR ( TITLE-ABS-KEY ( pkpd OR pk/pd OR tdm ) ) OR ( TITLE-ABS ( ( drug OR antibiotic* ) PRE/2 ( dosing OR dosage* OR regime* OR schedul* OR monitor* ) ) ) OR ( TITLE-ABS ( ( drug OR antibiotic* ) PRE/2 ( adjust* OR personali* OR optimi* ) ) ) OR ( TITLE-ABS ( dose PRE/2 ( adjust* OR alter* OR escalat* OR optimi* OR personali* ) ) ) OR ( TITLE-ABS ( ( drug OR antibiotic ) W/2 ( level* OR measur* OR concentration* ) ) ) ) AND ( TITLE-ABS-KEY ( meropenem* OR mepem OR meronem OR meropen OR merrem OR ronem ) ) ) ) AND NOT ( ( INDEXTERMS ( nonhuman ) AND NOT INDEXTERMS ( human ) ) OR ( INDEXTERMS ( "animal experiment" ) ) OR ( INDEXTERMS ( "experimental animal" ) ) OR ( INDEXTERMS ( animals OR animal ) AND NOT INDEXTERMS ( humans OR human ) ) ) Show more 5,112 results

22 ( INDEXTERMS ( nonhuman ) AND NOT INDEXTERMS ( human ) ) OR ( INDEXTERMS ( "animal experiment" ) ) OR ( INDEXTERMS ( "experimental animal" ) ) OR ( INDEXTERMS ( animals OR animal ) AND NOT INDEXTERMS ( humans OR human ) ) Show more 7,443,989 results

21 INDEXTERMS ( nonhuman ) AND NOT INDEXTERMS ( human ) 4,579,055 results

20 INDEXTERMS ( "animal experiment" ) 2,794,347 results

19 INDEXTERMS ( "experimental animal" ) 20,508 results

18 INDEXTERMS ( animals OR animal ) AND NOT INDEXTERMS ( humans OR human ) 5,439,530 results

17 ( TITLE-ABS-KEY ( ( meropenem* OR mepem OR meronem OR meropen OR merrem OR ronem ) W/2 ( adjust* OR personali* OR optimi* ) ) ) OR ( TITLE-ABS-KEY ( ( meropenem* OR mepem OR meronem OR meropen OR merrem OR ronem ) W/2 ( dosing OR dosage* OR regime* OR schedul* OR monitor* ) ) ) OR ( TITLE-ABS-KEY ( ( meropenem* OR mepem OR meronem OR meropen OR merrem OR ronem ) W/2 ( level* OR measur* OR concentration* ) ) ) OR ( ( ( INDEXTERMS ( "drug monitoring" ) ) OR ( INDEXTERMS ( "pharmacodynamics" OR "pharmacokinetics" ) ) OR ( TITLE-ABS ( ( drug OR antibiotic ) W/2 ( level* OR measur* OR concentration* ) ) ) OR ( TITLE-ABS ( ( blood OR serum OR plasma ) W/2 ( level* OR measur* OR concentration* ) ) ) OR ( TITLE-ABS ( pharmacokinetic* OR pharmacodynamic* ) ) OR ( TITLE-ABS-KEY ( pkpd OR pk/pd OR tdm ) ) OR ( TITLE-ABS ( ( drug OR antibiotic* ) PRE/2 ( dosing OR dosage* OR regime* OR schedul* OR monitor* ) ) ) OR ( TITLE-ABS ( ( drug OR antibiotic* ) PRE/2 ( adjust* OR personali* OR optimi* ) ) ) OR ( TITLE-ABS ( dose PRE/2 ( adjust* OR alter* OR escalat* OR optimi* OR personali* ) ) ) OR ( TITLE-ABS ( ( drug OR antibiotic ) W/2 ( level* OR measur* OR concentration* ) ) ) ) AND ( TITLE-ABS-KEY ( meropenem* OR mepem OR meronem OR meropen OR merrem OR ronem ) ) ) Show more 5,850 results

16 TITLE-ABS-KEY ( ( meropenem* OR mepem OR meronem OR meropen OR merrem OR ronem ) W/2 ( adjust* OR personali* OR optimi* ) ) Show more 76 results

15 TITLE-ABS-KEY ( ( meropenem* OR mepem OR meronem OR meropen OR merrem OR ronem ) W/2 ( dosing OR dosage* OR regime* OR schedul* OR monitor* ) ) Show more 401 results

14 TITLE-ABS-KEY ( ( meropenem* OR mepem OR meronem OR meropen OR merrem OR ronem ) W/2 ( level* OR measur* OR concentration* ) ) Show more 716 results

13 ( ( INDEXTERMS ( "drug monitoring" ) ) OR ( INDEXTERMS ( "pharmacodynamics" OR "pharmacokinetics" ) ) OR ( TITLE-ABS ( ( drug OR antibiotic ) W/2 ( level* OR measur* OR concentration* ) ) ) OR ( TITLE-ABS ( ( blood OR serum OR plasma ) W/2 ( level* OR measur* OR concentration* ) ) ) OR ( TITLE-ABS ( pharmacokinetic* OR pharmacodynamic* ) ) OR ( TITLE-ABS-KEY ( pkpd OR pk/pd OR tdm ) ) OR ( TITLE-ABS ( ( drug OR antibiotic* ) PRE/2 ( dosing OR dosage* OR regime* OR schedul* OR monitor* ) ) ) OR ( TITLE-ABS ( ( drug OR antibiotic* ) PRE/2 ( adjust* OR personali* OR optimi* ) ) ) OR ( TITLE-ABS ( dose PRE/2 ( adjust* OR alter* OR escalat* OR optimi* OR personali* ) ) ) OR ( TITLE-ABS ( ( drug OR antibiotic ) W/2 ( level* OR measur* OR concentration* ) ) ) ) AND ( TITLE-ABS-KEY ( meropenem* OR mepem OR meronem OR meropen OR merrem OR ronem ) ) Show more 5,416 results

12 ( INDEXTERMS ( "drug monitoring" ) ) OR ( INDEXTERMS ( "pharmacodynamics" OR "pharmacokinetics" ) ) OR ( TITLE-ABS ( ( drug OR antibiotic ) W/2 ( level* OR measur* OR concentration* ) ) ) OR ( TITLE-ABS ( ( blood OR serum OR plasma ) W/2 ( level* OR measur* OR concentration* ) ) ) OR ( TITLE-ABS ( pharmacokinetic* OR pharmacodynamic* ) ) OR ( TITLE-ABS-KEY ( pkpd OR pk/pd OR tdm ) ) OR ( TITLE-ABS ( ( drug OR antibiotic* ) PRE/2 ( dosing OR dosage* OR regime* OR schedul* OR monitor* ) ) ) OR ( TITLE-ABS ( ( drug OR antibiotic* ) PRE/2 ( adjust* OR personali* OR optimi* ) ) ) OR ( TITLE-ABS ( dose PRE/2 ( adjust* OR alter* OR escalat* OR optimi* OR personali* ) ) ) OR ( TITLE-ABS ( ( drug OR antibiotic ) W/2 ( level* OR measur* OR concentration* ) ) ) Show more 1,813,232 results

11 TITLE-ABS ( ( drug OR antibiotic ) W/2 ( level* OR measur* OR concentration* ) ) 109,864 results

10 TITLE-ABS ( dose PRE/2 ( adjust* OR alter* OR escalat* OR optimi* OR personali* ) ) 43,605 results

9 TITLE-ABS ( ( drug OR antibiotic* ) PRE/2 ( adjust* OR personali* OR optimi* ) ) 8,704 results

8 TITLE-ABS ( ( drug OR antibiotic* ) PRE/2 ( dosing OR dosage* OR regime* OR schedul* OR monitor* ) ) 60,060 results

7 TITLE-ABS-KEY ( pkpd OR pk/pd OR tdm ) 9,497 results

6 TITLE-ABS ( pharmacokinetic* OR pharmacodynamic* ) 268,233 results

5 TITLE-ABS ( ( blood OR serum OR plasma ) W/2 ( level* OR measur* OR concentration* ) ) 1,310,427 results

4 TITLE-ABS ( ( drug OR antibiotic ) W/2 ( level* OR measur* OR concentration* ) ) 109,864 results

3 INDEXTERMS ( "pharmacodynamics" OR "pharmacokinetics" ) 221,189 results

2 INDEXTERMS ( "drug monitoring" ) 68,007 results

1 TITLE-ABS-KEY ( meropenem* OR mepem OR meronem OR meropen OR merrem OR ronem ) 52,591 results
